# Nature-based outdoor activities for mental and physical health: Systematic review and meta-analysis

**DOI:** 10.1016/j.ssmph.2021.100934

**Published:** 2021-10-01

**Authors:** Peter A. Coventry, JenniferV.E. Brown, Jodi Pervin, Sally Brabyn, Rachel Pateman, Josefien Breedvelt, Simon Gilbody, Rachel Stancliffe, Rosemary McEachan, PiranC.L. White

**Affiliations:** a Department of Health Sciences, University of York, York, YO10 5DD, UK; b York Environmental Sustainability Institute, University of York, York, YO10 5NG, UK; c Stockholm Environment Institute, Department of Environment and Geography, University of York, York, YO10 5NG, UK; d National Centre for Social Research, London, United Kingdom; e Hull York Medical School, University of York, York, YO10 5DD, UK; f Centre for Sustainable Healthcare, Oxford, OX2 7JQ, UK; g Bradford Institute for Health Research, Bradford, BD9 6RJ, UK; h Department of Environment and Geography, University of York, York, YO10 5NG, UK

**Keywords:** Greenspace, Bluespace, Nature-based intervention, Mental health, Physical health

## Abstract

Mental health problems are associated with lower quality of life, increased unscheduled care, high economic and social cost, and increased mortality. Nature-based interventions (NBIs) that support people to engage with nature in a structured way are asset-based solutions to improve mental health for community based adults. However, it is unclear which NBIs are most effective, or what format and dose is most efficacious. We systematically reviewed the controlled and uncontrolled evidence for outdoor NBIs. The protocol was registered at PROSPERO (CRD42020163103). Studies that included adults (aged ≥18 years) in community-based settings with or without mental and/or physical health problems were eligible for inclusion. Eligible interventions were structured outdoor activities in green and/or blue space for health and wellbeing. We searched ASSIA, CENTRAL, Embase, Greenfile, MEDLINE, PsycINFO, and Web of Science in October 2019; the search was updated in September 2020. We screened 14,321 records and included 50 studies. Sixteen studies were randomised controlled trials (RCTs); 18 were controlled studies; and 16 were uncontrolled before and after studies. Risk of bias for RCTs was low to moderate; and moderate to high for controlled and uncontrolled studies. Random effects meta-analysis of RCTs showed that NBIs were effective for improving depressive mood −0.64 (95% CI: 1.05 to −0.23), reducing anxiety −0.94 (95% CI: 0.94 to −0.01), improving positive affect 0.95 (95% CI: 0.59 to 1.31), and reducing negative affect −0.52 (95% CI: 0.77 to −0.26). Results from controlled and uncontrolled studies largely reflected findings from RCTs. There was less evidence that NBIs improved physical health. The most effective interventions were offered for between 8 and 12 weeks, and the optimal dose ranged from 20 to 90 min. NBIs, specifically gardening, green exercise and nature-based therapy, are effective for improving mental health outcomes in adults, including those with pre-existing mental health problems.

## 1 Introduction

Mental health disorders are the third leading cause of years lived with disability, with a global prevalence of greater than 10% (James et al., 2018). The lifetime prevalence for major depressive disorders in the general population in the United States has increased during the past 25 years and is estimated to be 20.6% (Hasin et al., 2018). Generalised anxiety disorder is also a relatively common mental health problem with a lifetime prevalence of 5.7% (Kessler et al., 2005). In England less severe and common mental health problems affect about 1 in 6 people (Bebbington et al., 2020). Globally, lost productivity associated with common mental health problems such as depression and anxiety is estimated at US $1 trillion each year (Chisholm et al., 2016). In the UK, mental health problems are among the main reasons for sickness absence (Office for National Statistics, 2021b) and the social and economic cost of mental health in England has grown in the last decade to £119 billion a year (Centre for Mental Health, 2020b).

Over the past two decades the global burden of disease has significantly shifted away from communicable to non-communicable long-term conditions with more years lost to disability from cancers, cardiovascular diseases, musculoskeletal disorders, chronic respiratory diseases, digestive diseases, and diabetes, especially among older adults (Murray et al., 2012). People with long-term conditions are two to three times more likely to experience depression than the general population. The combination of depression and long-term conditions is associated with the largest decrements in quality of life (Moussavi et al., 2007), greater use of unscheduled care (Guthrie et al., 2016), and increases the cost of care for patients by at least 45% (Naylor et al., 2012). In people with serious mental illness such as schizophrenia and bipolar disorder, life expectancy is shortened by 10–20 years compared with the general population (Walker et al., 2015), and this mortality gap is widening (Hayes et al., 2017). It is estimated that two thirds of deaths among people with serious mental illness are attributable to preventable long-term conditions such as cardiovascular disease, respiratory disease, diabetes, and hypertension (Correll et al., 2017).

During the Covid-19 pandemic the prevalence of mental health problems has increased. In England 1 in 5 adults experienced some form of depression in the first quarter of 2021 – more than double the rate observed before the pandemic (Office for National Statistics, 2021a). Forecasts modelled on community-based surveys suggest that in England 10 million people (8 million adults) will need new or additional mental health support. About two-thirds of these have pre-existing mental health needs, but at least 3.1 million people who have not previously had mental health problems will need support for depression, anxiety, or both (Centre for Mental Health, 2020a). Traditional health service models and interventions are unlikely to meet this demand and whole system approaches that strengthen community resilience and capacity to improve population health are therefore needed.

Salutogenic or asset-based approaches are critical to this shift away from pathogenic models of health and healthcare (Lindström et al., 2005). Green environments or green spaces have high salutogenic potential. Green spaces are purported to be health protective because they can function as restorative spaces; or spaces to support social interaction and physical activity; and they can mitigate the negative impacts of air, noise, and heat pollution (Sarkar et al., 2018). In the UK there has been considerable investment in nature-based social prescribing to increase use of and access to green spaces, with a focus on targeting communities whose mental health has been disproportionately affected by Covid-19 (HM Government, 2021). Nature-based social prescribing aims to link people with defined health needs to specifically designed, structured and facilitated nature-based interventions delivered in the community. While there is accumulating evidence that exposure to green (publicly accessible areas with natural vegetation) and blue (outdoor water environments) spaces is associated with mental and physical health benefits (Hartig et al., 2014; WHO, 2016), much of this evidence is largely based on understanding the health protective effects of proximity to green space.

Evidence about the health benefits associated with engaging with nature-based interventions is less clear. A scoping review of the impact of gardens and gardening on health and wellbeing showed that viewing gardens, taking part in gardening, or undertaking therapeutic activities were associated with improved wellbeing, increased physical activity and reduced social isolation (Howarth et al., 2020). This review only focused on gardening and included studies drawn from heterogenous populations (e.g. dementia; substance abuse, children, older adults) and settings (e.g. care homes, schools), making it difficult to draw firm conclusions about the utility of a broader suite of nature-based interventions for adults in community settings. A broader review that included quantitative and qualitative studies across diverse populations concluded that social and therapeutic horticulture and wilderness therapy are associated with significant improvements in mental and physical health outcomes, including for people with obesity and schizophrenia (Annerstedt et al., 2011). However, this review did not include a meta-analysis and instead narratively summarised findings from low quality small studies that used a mix of validated and non-validated health outcomes, reducing its relevance to decision making about which nature-based interventions are likely to be most effective. The only meta-analysis of gardening for health included 76 comparisons across 22 studies, with six studies showing significant improvements in depression (Soga et al., 2017). Effect sizes were however not pooled or derived from randomised controlled trials (RCTs), limiting the usefulness of this review for definitively determining the overall effectiveness of gardening for mental health. A recent mixed-methods review of nature-based social prescribing for people with diagnosed mental health included only four RCTs (Garside et al., 2020). Results from this review suggested that nature-based interventions may positively impact on depression, anxiety, mood and feelings of hope. Additionally, existing reviews have not addressed questions related to optimal dose or format of interventions, reducing credibility of claims that there is sufficient evidence to support widescale implementation of nature-based interventions.

If population health approaches such as nature-based social prescribing are to be effective it is critical that candidate interventions most likely to be effective for improving health outcomes in well-defined populations are systematically identified and described. Furthermore, to support the most effective scale up and roll out beyond research contexts it is important that decision makers responsible for commissioning population health interventions are also provided with robust and comprehensive assessments of the optimal delivery, format, and dose of nature-based interventions. To take a first step in addressing these research objectives we have therefore undertaken a systematic review with meta-analysis to summarise the evidence across controlled and uncontrolled studies about the effectiveness of outdoor nature-based interventions for mental and physical health outcomes in adults in community settings.

## 2 Methods

### 2.1 Protocol registration

The protocol for this study was registered on PROSPERO (CRD42020163103). We followed the PRISMA 2020 statement checklist (Page et al., 2021) which is available in the supplementary material.

### 2.2 Eligibility criteria

Population: Adults (aged ≥18 years) in community based (non-hospital) settings with or without mental and/or physical health problems.

Interventions: Nature-based interventions included independent or group based activities that are undertaken in outdoor green and blue spaces. We defined greenspace as publicly accessible areas with natural vegetation, such as grass, plants or trees. These included formal spaces, such as parks and outdoor sports fields, as well as more natural areas including woodlands and nature reserves. Greenspaces were in urban, rural and semi-rural areas that immediately adjoin an urban area (Lachowycz et al., 2013). Excluded greenspaces were working farms that do not permit volunteering, inaccessible wilderness, and agricultural land. Bluespace was defined as either man made (e.g. canals; boating lakes) or naturally occurring fresh water (e.g. rivers; ponds; lakes) or salt water bodies with identifiable potential for promotion of human wellbeing (Foley et al., 2015). Interventions categories included:

• Social and therapeutic horticultural activities such as gardening and food growing to support wellbeing
• Care farming that involves the therapeutic use of agricultural landscape and farming practices
• Environmental conservation that involves activities designed for conservation and management of natural places for health and wellbeing
• Green and blue exercise that involves physical activity, including walking and moderate to vigorous activity such as jogging
• Nature-based therapies that include the therapeutic use of natural spaces to undertake stress relieving and relaxing activities, such as forest bathing, mindfulness, and wilderness therapy
• Nature-based arts and crafts tasks that involve being in nature and using natural materials to construct artefacts.

Activities that are routinely undertaken as part of occupational roles (e.g. park ranger; nature reserve management; farming) and sport activities undertaken outdoors where nature is not an essential component were not eligible.

Comparators: For experimental studies, the comparators included a broad range of controls: attention controls either in outdoor or indoor spaces; non-nature based equivalent activities; or usual care for those studies that recruited participants with identified physical and/or mental health problems from primary or secondary healthcare pathways.

Outcomes: To be included studies had to report at least one of the primary outcomes or one of the candidate secondary outcomes reported in the section on data collection items. The primary outcomes were changes in subjectively measured and self-reported physical health and/or mental health symptoms on continuous scales. Studies that only reported non-patient centric biochemical outcomes (e.g. inflammatory markers such as C-reactive protein) were excluded.

Study design: We included randomised controlled trials (RCTs), controlled studies, and single group before and after studies of outdoor nature-based interventions. Case-controlled studies were not included. Only peer reviewed studies were eligible; case series and case studies, editorials and expert opinion pieces were excluded.

### 2.3 Information sources

We searched ASSIA, Cochrane Central Register of Controlled Trials (CENTRAL), Embase, Greenfile, MEDLINE, PsycINFO, and Web of Science. Records were also selected and downloaded from the University of Exeter website Beyond Greenspace: https://beyondgreenspace.net/. The search strategy from a Cochrane review of participation in environmental enhancement and conservation activities was used with permission (Husk et al., 2016). The search strategies used in the Cochrane review were revised and rerun separately in ASSIA, Cochrane CENTRAL, Greenfile, and Web of Science. For Embase, MEDLINE and PsycINFO the revised Cochrane Review search was run alongside the newly developed search. The searches were originally run on October 19, 2019 and updated in Medline, PsycInfo, EMBASE and Greenfile on September 24, 2020.

### 2.4 Search strategy

The full search strategies for each database are presented in Appendix A. Searches were restricted to studies in high income countries because we wanted to maximise the chances to translate findings from the review to inform health policy in comparable high-income health service and policy contexts. We anticipated that the provision and availability of nature-based interventions in low and middle income countries would likely be very different, given the marked differences in publicly accessible green and bluespaces for health and wellbeing, limiting relevance of these studies for our context. There has been an increase in the last decade of studies about green space or greenspace and we therefore limited our database searches from January 1, 2010 (Taylor et al., 2017). We did not restrict on language and translated studies where feasible, but we did not search Chinese databases or translate this language.

### 2.5 Selection process

Records from database searches were imported into EndNote and de-duplicated. Priority screening was facilitated by excluding studies that included key words associated with non-green and blue space topic areas associated with atmospheric chemistry and the physical and life sciences: bioresource; atmospheric; chemosphere; s oil; toxicology; pollution; and microbiology. We also excluded studies published in the journal Science of the Total Environment which focuses on topics related to the atmosphere, lithosphere, hydrosphere, biosphere, and anthroposphere. The remaining EndNote library was then uploaded to Covidence (Covidence), which is a cloud based systematic review production tool for title/abstract and full text screening, allowing for multiple researchers to undertake screening tasks at distance. Five researchers (PC, JP, SB, JB, JBreedvelt) independently reviewed titles and abstracts of the first 100 records and discussed inconsistencies until consensus was obtained. Then, in pairs, the researchers independently screened titles and abstracts of all articles retrieved. Disagreements were flagged in Covidence and a third reviewer was invited to reach consensus about inclusion. Five researchers (PC, JP, SB, JB, RP) then screened the full texts in pairs, with a third reviewer used to reach consensus about inclusion in the presence of conflicts. The same process was repeated for the updated search, but without the need for an initial check on a proportion of the records.

### 2.6 Data collection process

All eligible studies were saved as a PDF and uploaded to a shared Google drive. We designed a Google form for five researchers (PC, JB, JP, SB, RP) to extract data from eligible studies. Data items extracted using this form were: study author name; year of publication; full study title; country of origin; study design; sample population age (18–25; 26–35; 46–55; 56–55; over 65; not specified); population characteristics (university students, non-student healthy volunteers, clinical or population based samples with serious mental illness, common mental health problems, physical health problem/long-term condition); sample size; mean age (standard deviation [SD]); % female; % ethnicity; type of intervention (green/blue space); type of activity (physical activity; creative tasks; conservation; horticulture; ecotherapy; other); comparator; mental health outcomes (wellbeing, common mental health problems, serious mental illness, cognitive function; loneliness, stress, other); and physical health outcomes (quality of life, activities of daily living, functioning, disability, acute stress, cardiovascular outcomes, other). This Google form auto-populated a Google spreadsheet with extracted data related to these data items. One researcher (PC) independently exacted data into a bespoke data extraction form for outcomes by study type (RCT, controlled study, single group before and after study); risk of bias; outcomes (mean, SD; median, inter-quartile range).

### 2.7 Data collection items

Eligible mental health outcomes were defined as:

• Anxiety symptoms: measured using validated self-reported scales [e.g. Generalised Anxiety Disorder-7 (Spitzer et al., 2006)];
• Depressive symptoms: measured using validated self-reported scales [e.g. PHQ-9 (Kroenke et al., 2001)];
• Positive and negative affect measured using validated scales [e.g. Positive and Negative Affect Schedule (Watson et al., 1988)];
• Variability in mood across a number of emotional states, including tension or anxiety and depression or dejection, measured using validated scales [e.g. Profile of Mood States (Shacham, 1983)]
• Eudaimonic and/or hedonic wellbeing measured using validated scales [e.g. Warwick-Edinburgh Mental Wellbeing Scale (Stewart-Brown et al., 2009)]; and
• Loneliness defined as the subjective psychological expression of social isolation owing to dissatisfaction with the frequency and quality of social contacts measured using validated self-reported scales [e.g. De Jong Gierveld Loneliness scale (De Jong Gierveld et al., 2010)];

Eligible physical health outcomes were defined as:

• Activities of daily living (functional competence in everyday higher level tasks such as shopping or preparing a meal) measured using validated self-report scales [e.g. Nottingham Extended Activities of Daily Living Scale (Gladman et al., 1993)];
• Functioning and disability (impairments in work/school, social and family life) measured using validated self-reported scales [e.g. Sheehan Disability Scale (Sheehan et al., 1996)]
• Objectively measured risk factors for cardiovascular health (e.g. BMI, blood pressures, lipids);
• Physical activity measured using subjectively assessed self-report scales and/or pedometers/accelerometers.

Data items that were collected using self-reported scales were extracted at baseline and at follow-up at a time point most common across the studies (i.e. within six months of intervention end) to maximise opportunities for comparison across studies. Data items that were collected using instruments (e.g. accelerometers) or blood tests (e.g. lipids) were extracted at baseline and at intervention end as these outcomes recorded the acute impact of interventions on health outcomes.

### 2.8 Risk of bias

Risk of bias for RCTs was assessed with the Cochrane Risk of Bias tool (Higgins et al., 2011). This tool assesses each study against domains known to be associated with bias in randomised controlled trials: selection, performance, detection, attrition, reporting, and other biases. We did not assess blinding of participants to interventions given that this approach is not feasible in this context. Studies were assessed as being at either ‘low’, ‘unclear’ or ‘high’ risk of bias across each of these domains. Using methods previously applied in meta-analyses RCTs were classified as having low risk of bias if none of the domains were rated as high risk of bias and three or less were rated as unclear risk; moderate if one was rated as high risk of bias or none was rated as high risk of bias but four or more were rated as unclear risk (Furukawa et al., 2016). All other cases were assumed to be at high risk of bias.

Controlled and single group before and after studies were assessed for risk of bias using a modified version of the NICE (2012) quality appraisal checklist (National Institute for Health and Care Excellence, 2012). This checklist was originally developed based on the ‘Graphical Appraisal Tool for Epidemiological studies’ (GATE) tool, and includes domains of population bias, allocation, outcomes and analyses, as well as summary judgements for internal and external validity (Jackson et al., 2006). We rated risk of bias of controlled and single group before and after studies by using cumulative ratings of internal and external validity. Risk of bias was rated as low (all positive ratings), moderate (balance between positive and negative ratings), and high (all negative ratings).

### 2.9 Effect measures and synthesis methods

To maximise opportunities to meta-analyse mental health outcomes across interventions we constructed an omnibus measure of low mood and depressive symptoms. This omnibus measure did not include items related to anxiety. Anxiety and low mood correlate highly but there is evidence that they are distinct constructs. According to the tripartite model of depression and anxiety, negative affect is common to both, but high physiological arousal plus negative affect is present in anxiety alone, whilst low positive affect (anhedonia) plus negative affect is present in depression (Clark et al., 1991). We therefore reported effect measures for anxiety separately to those for the omnibus measure for depressive mood.

Additionally, as we have done in previous systematic reviews of complex interventions (Coventry et al., 2020), we mapped intervention content to three super-ordinate categories to further enhance opportunities to meta-analyse outcomes across studies that tested interventions with similar core components. All interventions that included forms of horticultural activity were categorised as ‘gardening’. Interventions that included forms of physical activity were categorised as ‘green exercise’. And interventions that involved immersive experiences in natural environments with a focus on connections with nature were categorised as ‘nature-based therapies’.

We used random-effects meta-analysis models that estimated between-study variance with the DerSimonian and Laird approach. For each study (*k=*number of studies) that included continuous outcomes, a standardised mean difference (SMD) was calculated by taking the mean of the intervention group minus the mean of the control group, divided by the pooled SD. Effect sizes expressed as SMDs are a useful method to compare the effect of an intervention across studies when different measures (such as different depression scales) are used. Additionally, the SMD is more generalizable and statistically powerful in meta-analyses of continuous outcomes when the same unit is used (Takeshima et al., 2014). We draw on established cut-offs used in behavioural science to effect sizes whereby SMDs of 0.56–1.2 were categorised as large; effect sizes of 0.33–0.55 as moderate, and effect sizes ≤0.32 as small (Lipsey et al., 1993). In this review, negative effect sizes indicated that the intervention improved mental health outcomes; statements about significance refer to statistical significance within 95% confidence intervals. Where exact means and SDs were missing from published reports or not provided by the authors we estimated effect sizes using conventional methods (Lipsey et al., 2001), from exact P values or from a figure shown in the articles reviewed. We converted standard errors of means by multiplying by the square root of the sample size (Higgins et al., 2021). If a SD was missing we imputed SDs from a comparable study in the meta-analysis that used the same measure. Where trials reported two intervention groups and a single control group, separate SMDs were calculated for each intervention group but in the pooled analyses the sample size of the control group was halved to avoid double counting. Methods devised by Hedges and Olkin were used to correct for small sample size bias (Hedges et al., 1985). Separate meta-analyses were undertaken for RCTs and controlled studies. Random-effects pair-wise meta-analyses were conducted using Stata 15 (StataCorp, 2017). For single group before and after studies we constructed SMDs using a meta-analysis add-in for Microsoft Excel (Kontopantelis et al., 2009). Only studies that reported sufficient outcome data or data that could be imputed or converted to formats for constructing SMDs were included in the meta-analyses.

Heterogeneity was analysed with the I^2^ index which represents the percentage of the total variability in a set of effect sizes due to between-study variability, rather than sampling error alone (Higgins et al., 2002); and by using Cochran's Q test, which is calculated as the weighted sum of squared differences between individual study effects and the pooled effect across studies. A Q-value (approximating Χ^2^ distribution) of p<0.1 indicated statistically significant heterogeneity. Effect estimates and confidence intervals of each study were graphically displayed using forest plots. To explore heterogeneity we visually inspected forest plots as well as calculating I^2^ statistic. Forest plots were graphed for each intervention sub-group and included a pooled overall effect for each outcome. It was not possible to assess publication bias and construct funnel plots because of small numbers of studies per intervention sub-group. Typically tests for funnel plot asymmetry should only be used where there are at least 10 studies in each meta-analysis (Sterne et al., 2011).

## 3 Results

### 3.1 Study characteristics

**Fig. 1:**
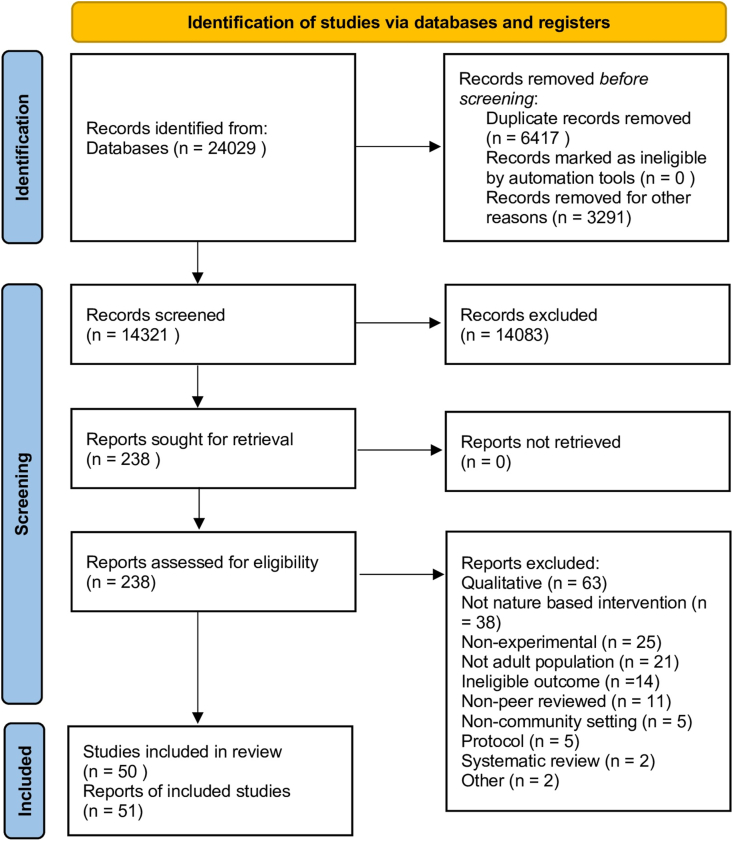
PRISMA flow diagram.

We identified 24,029 records from database searches. After de-duplication and removing records from non-relevant journal sources we screened 14,321 titles and abstracts. Of these we retrieved 238 records to review full texts. We finally included 50 studies reported in 51 articles. The study selection process and reasons for exclusion are presented in the PRISMA flow diagram in Fig. 1.

Sixteen studies were RCTs (Bail et al., 2018; Bay-Richter et al., 2012; Bielinis et al., 2021; Brown et al., 2014, Brown et al., 2020; Calogiuri et al., 2016; Gidlow et al., 2016; Han et al., 2018; Martens et al., 2011; Müller-Riemenschneider et al., 2020; Ng et al., 2018; Olafsdottir et al., 2020; Song et al., 2015, 2018; Van Den Berg et al., 2011; Vujcic et al., 2017); eighteen studies (19 papers) were controlled studies (Bang et al., 2017; Barton et al., 2010; Berman et al., 2012; de Brito et al., 2019; Gerber et al., 2017; Hawkins et al., 2015; Holt et al., 2019; Johansson et al., 2011; Lanki et al., 2017; Lee et al., 2011, Lee et al., 2014; Lucke et al., 2019; Lyu et al., 2019; Marselle et al., 2013; Oh et al., 2018; Sin-Ae et al., 2016, 2017; Yao et al., 2017; Zeng et al., 2020); and sixteen studies were single group before and after designs (Bettmann et al., 2017; Coventry et al., 2019; Furuyashiki et al., 2019; Gonzalez et al., 2011; Hall et al., 2018; Iwata et al., 2016; Kling et al., 2018; Korpela et al., 2016; Mackay et al., 2010; Marselle et al., 2016; McCaffrey et al., 2016; Mourão et al., 2019; Warber et al., 2015; Wilson et al., 2011; Wood et al., 2016; Wyles et al., 2017).

Over half of the studies were conducted in Europe (n=25); fourteen in Asia; ten in North America; and one in Australia. Samples in 24 studies were drawn from healthy adult non-clinical populations (Bielinis et al., 2021; Brown et al., 2014; Calogiuri et al., 2016; de Brito et al., 2019; Furuyashiki et al., 2019; Gerber et al., 2017; Gidlow et al., 2016; Hawkins et al., 2015; Kling et al., 2018; Lanki et al., 2017; Lee et al., 2014; Lucke et al., 2019; Mackay et al., 2010; Marselle et al., 2013, Marselle et al., 2016; McCaffrey et al., 2016; Mourão et al., 2019; Müller-Riemenschneider et al., 2020; Ng et al., 2018; Sin-Ae et al., 2017; Song et al., 2018; Van Den Berg et al., 2011; Warber et al., 2015). Eight studies recruited university student volunteers (Bang et al., 2017; Han et al., 2018; Holt et al., 2019; Lee et al., 2011; Olafsdottir et al., 2020; Zeng et al., 2020). Participants with physical health problems, including long term conditions, were recruited in five studies (Bail et al., 2018; Brown et al., 2020; Hall et al., 2018; Song et al., 2015; Yao et al., 2017). Five studies recruited participants with common mental health problems (Barton et al., 2010; Bay-Richter et al., 2012; Berman et al., 2012; Korpela et al., 2016; Vujcic et al., 2017); four studies recruited participants with a mix of common mental health problems and serious mental illness (Bettmann et al., 2017; Coventry et al., 2019; Gonzalez et al., 2011; Wilson et al., 2011); and two studies exclusively recruited participants with serious mental illness (Iwata et al., 2016; Oh et al., 2018).

The two most commonly tested interventions were gardening (n=16 studies) and green and/or blue exercise (n=25 studies). Other interventions included nature-based therapy (n=8 studies), and conservation (n=1 study). Across all studies the age of participants ranged from 19 to 84 years, with a mean of 44.4 years. The mean age of participants in studies of gardening was 61.8 years; the mean age of participants in studies of green and/or blue exercise was 38.8 years; and the mean age of participants in studies of nature-based therapy was 32.4 years. The majority tested interventions in green space with only four studies testing interventions in a combination of green and blue spaces (Gidlow et al., 2016; Holt et al., 2019; Mackay et al., 2010; Marselle et al., 2013), and only one studied tested an intervention exclusively in a blue space (Wyles et al., 2017). Thirteen studies tested group-based gardening interventions and three tested gardening among individuals. Green and/or blue exercise interventions were fairly evenly split between group formats (n=14 studies) and individual formats (n=11 studies). Six out of eight nature-based therapeutic interventions were delivered in groups, as was the conservation activity tested in one study. Gardening interventions were delivered for an average of 11.6 (SD=6.58) weeks, while green and/or blue exercise interventions were delivered for an average of 6 (SD=6.92) weeks. Nature-based therapy interventions were delivered for an average of 5.2 (SD=4.76) weeks; the conservation activity in one study was run for two weeks. Full study and intervention characteristics are shown in Tables B.1 and B.2 respectively in Appendix B.

### 3.2 Risk of bias

Ten and six RCTs were categorised as being of low and moderate risk of bias respectively. For RCTs the risk of bias from random sequence generation was low in four (25%) studies; and low for allocation concealment in three (19%) studies. For non-RCTs the certainty of evidence based on risk of bias and threats to internal and external validity was moderate in 11 studies and very low in seven studies. The certainty of evidence from single group before and after studies was moderate in nine studies and very low in seven studies. A breakdown of risk of bias by individual domains for RCTs is shown in Table B.3 and for controlled studies and single group before and after studies in Table B.4 in Appendix B.

### 3.3 Results of syntheses

#### 3.3.1 Depressive mood omnibus

**Fig. 2:**
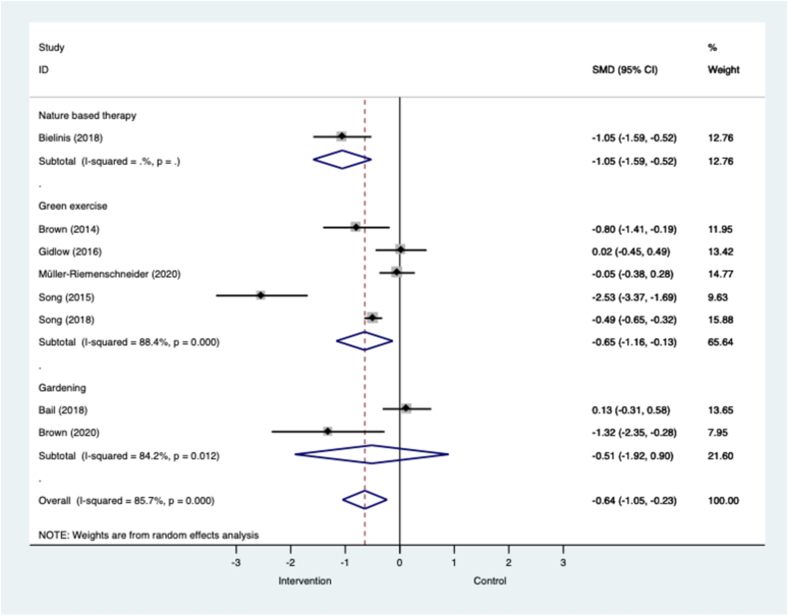
**Meta-analysis of RCTs of nature-based interventions for depressive mood versus control at post-intervention across all populations** The size of the grey box reflects how much weight each study received in the meta-analysis (i.e., the larger the box the more this study contributed to the pooled effect represented by the blue diamond). Black bars represent the 95% CI for the SMD in each study. (For interpretation of the references to colour in this figure legend, the reader is referred to the Web version of this article.) CI = confidence interval; RCT = randomised controlled trial; SMD = standardised mean difference.

Fig. 2 shows that across eight RCTs in all populations, nature-based interventions were associated with a large and significant effect in favour of reductions in symptoms of depressive mood. High levels of heterogeneity were observed in the pooled analysis across all eight RCTs (I^2^=85.7%; p=<0.001). When analysed by intervention sub-group we showed that green exercise interventions in mixed populations, including older adults with long-term conditions, were associated with large and significant effects (k=5; n=886; SMD=−0.65; −1.16 to −0.13; I^2^=88.4%, p=0.000). In the green exercise meta-analysis the most effective interventions were delivered in groups for between 20 and 50 min. There was mixed evidence from two small RCTs that gardening interventions are effective at reducing depressive mood, but the effects were non-significant (k=2; n=96; SMD=−0.51; −1.92 to 0.90; I^2^=84.2%, p=0.012). The one RCT that compared a one-time only group-based forest bathing intervention against an urban control resulted in a large and significant effect in favour of the intervention (k=1; n=62; SMD=−1.05; −1.59 to −0.52).

Similarly, Fig. C.1 shows that across ten controlled studies in all populations, nature-based interventions were associated with a large and significant effect in favour of reductions in symptoms of depressive mood. This result was associated with a high level of heterogeneity (I^2^=90.7%; p=0.000). Unlike the pooled results across RCTs, gardening interventions were associated with a large and significant effect in favour of reducing depressive symptoms in four controlled studies (k=4; n=201; SMD=−1.24, −2.25 to −0.22; I^2^=90.9, p=0.000). The high levels of heterogeneity observed are possibly driven by the difference in populations that included older non-clinical populations (Hawkins et al., 2015) and older adults with long term conditions (Yao et al., 2017), as well differences in controls, including indoor exercise (Hawkins et al., 2015). Results for green exercise were more equivocal in three controlled studies (k=3; n=270; SMD=−0.17, −0.42 to 0.07; I^2^=0.0%, p=0.730). All studies reported results that tended to favour the intervention except one that included people with common mental health problems (Barton et al., 2010). Effect sizes associated with nature-based therapy were large but not-significant in three controlled studies that all included university student volunteers (k=3; n=208; SMD=−1.39, −3.02 to 0.25; I^2^=93.4%, p=0.000). The overall 95% confidence intervals (CIs) for nature-based therapy were large, suggesting substantial imprecision.

Fig. C.2 shows that nature-based interventions across all populations were associated with moderate and significant effects for depressive mood at the end of the intervention in eight single-group before and after studies. The intervention types associated with significant and positive effects on depressive symptoms were green exercise (k=1; n=45; SMD=−0.65) and nature-based therapy (k=4; n=396; SMD=−0.28, −0.49 to −0.08; I^2^=49.5%, p=0.015). Effect sizes were small and non-significant in two studies of nature-based therapy that included populations with a mix of common mental health problems and serious mental illness (Bettmann et al., 2017; Wilson et al., 2011).

#### 3.3.2 Anxiety

**Fig. 3:**
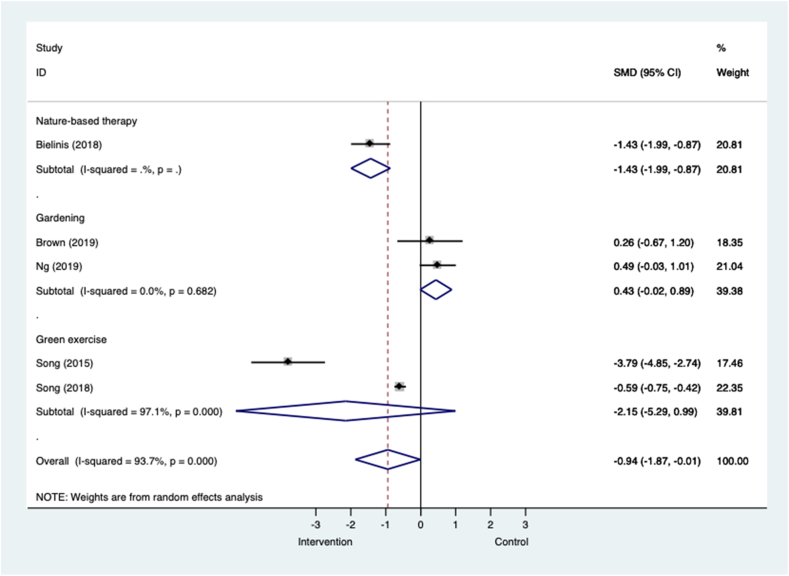
**Meta-analysis of RCTs of nature-based interventions for anxiety versus control at post-intervention across all populations** The size of the grey box reflects how much weight each study received in the meta-analysis (i.e., the larger the box the more this study contributed to the pooled effect represented by the blue diamond). Black bars represent the 95% CI for the SMD in each study. (For interpretation of the references to colour in this figure legend, the reader is referred to the Web version of this article.) CI = confidence interval; RCT = randomised controlled trial; SMD = standardised mean difference.

Fig. 3 shows that across five RCTs in all populations, nature-based interventions were associated with a large and significant effect size in favour of reducing anxiety symptoms. The 95% CIs for this pooled analysis were large and only marginally within the bounds of significance, suggesting substantial imprecision. When analysed by intervention sub-group gardening interventions in two small trials were associated with moderate effect sizes that favoured control, indicating increases in anxiety symptoms (k=2; n=50; SMD=0.43, −0.02 to 0.89; I^2^=0.0%, p=0.682). Green exercise was associated with very large but non-significant effects in favour of the intervention in two trials that compared one-off forest walks with urban walks in people with long term conditions and healthy volunteers (k=2; n=625; SMD=−2.15, −5.29 to 0.99; I^2^=97.1%; p=0.000). Nature-based therapy was associated with large and significant effects in favour of the intervention in one trial of healthy volunteers (k=1; n=62; SMD=−1.43, −1.9 to −0.87).

Fig.C.3 shows that across four controlled studies in all populations nature-based interventions are associated with large and significant effects in favour of reducing anxiety symptoms. This pooled analysis was associated with high levels of heterogeneity and large 95% CIs, suggesting substantial imprecision in the estimate. Effects were moderate to large for green exercise (k=2; n=102; SMD=−0.53, −1.27 to 0.21; I^2^=68.8%, p=0.041) and nature-based therapy (k=2; n=216; SMD=−3.22, −6.60 to 0.17; I^2^=98.0%, p=0.000) respectively, but non-significant. The result for one study that compared an 8-week course of forest therapy with a city walk in student volunteers (Zeng et al., 2020) was associated with very large effects and possibly skewed the pooled analysis in favour of the interventions.

Fig.C.4 shows that nature-based interventions were associated with large and significant effects for reducing anxiety symptoms in three uncontrolled before and after studies. Forest bathing in healthy adult volunteers was associated with a large and significant effect in favour of the intervention (k=1; n=155; SMD=−1.14, −1.38 to −0.90). A 12-week group gardening intervention in people with a mix of common mental health problems and serious mental illness was also associated with a large and significant effect (k=1; n=46; SMD=−0.67, −1.09 to −0.25). And in one study of green exercise, both orienteering and running were associated with a small but non-significant effect (k=1; n=34; SMD=−0.19, −0.66 to 0.29).

#### 3.3.3 Positive affect

**Fig. 4:**
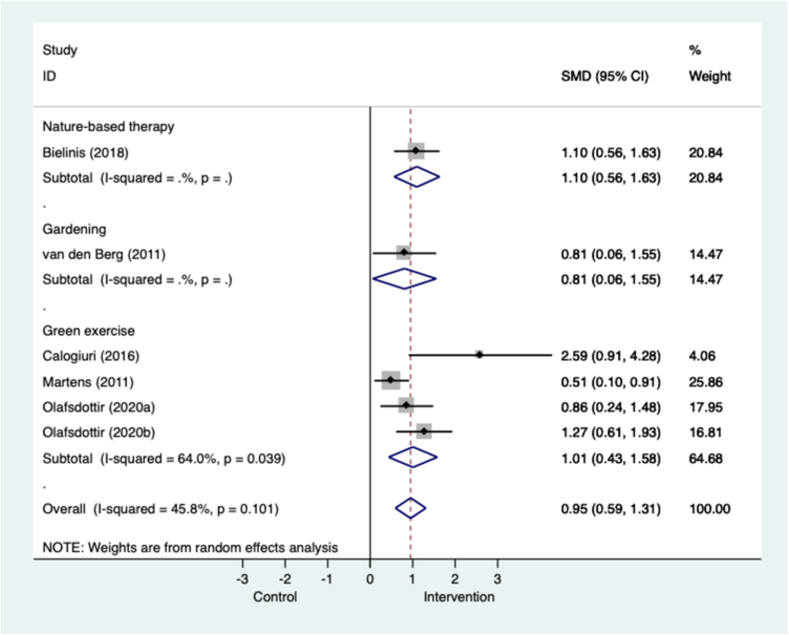
**Meta-analysis of RCTs of nature-based interventions for positive affect versus control at post-intervention across all populations** The size of the grey box reflects how much weight each study received in the meta-analysis (i.e., the larger the box the more this study contributed to the pooled effect represented by the blue diamond). Black bars represent the 95% CI for the SMD in each study. (For interpretation of the references to colour in this figure legend, the reader is referred to the Web version of this article.) CI = confidence interval; RCT = randomised controlled trial; SMD = standardised mean difference.

Fig. 4 shows that across five trials and in all populations, nature-based interventions were associated with large and significant effects in favour of enhancing positive affect, and displayed moderate heterogeneity. The largest effects were seen in one study of nature-based therapy in university student volunteers (k=1; n=62: SMD=1.10, 0.56 to 1.63). A two-week gardening intervention was also associated with large effects in one study of healthy adult volunteers (k=1; n=30; SMD=0.81.0.06 to 1.55). Green exercise interventions were all associated with significant effects in favour of enhancing positive affect in three trials of healthy adult volunteers (k=3; n=197; SMD=1.01, 0.43 to 1.58; I^2^=64.0%, p=0.039). In the green exercise sub-group exercise in a tended forest with footpaths was associated with stronger changes in positive affect than in a wild forest, not modified for walking (Martens et al., 2011). In one study the effects of green walks were attenuated in the comparison with indoor exercise compared to the comparison with viewing nature on television, which is a more inactive control (Olafsdottir et al., 2020). The most effective green exercise interventions were delivered for between 12 and 13 weeks.

Evidence from controlled studies tended to favour nature-based interventions but was non-significant (k=3; n=90; SMD=0.52, −0.12 to 1.15; I^2^=52.5%, p=0.122) (Fig. C.5). A study that compared a gardening intervention with treatment as usual in people with SMI favoured the control group (Oh et al., 2018). And changes in positive effect were less pronounced in a study that compared a green walk with an urban walk in people with common mental health problems (Berman et al., 2012).

Pooled results across four single group before and after studies resembled findings from RCTs. Fig.C.6 shows that all nature-based interventions across all populations were associated with improvements in positive affect (k=4; n=217; SMD=0.81, 0.61 to 1.00; I^2^=0.0%; p=0.788). Effects were smaller but still significant in one study that tested green exercise in groups of people with SMI (Iwata et al., 2016). A 12-week gardening intervention in people with SMI was associated with a large and significant effect (Gonzalez et al., 2011).

#### 3.3.4 Negative affect

**Fig. 5:**
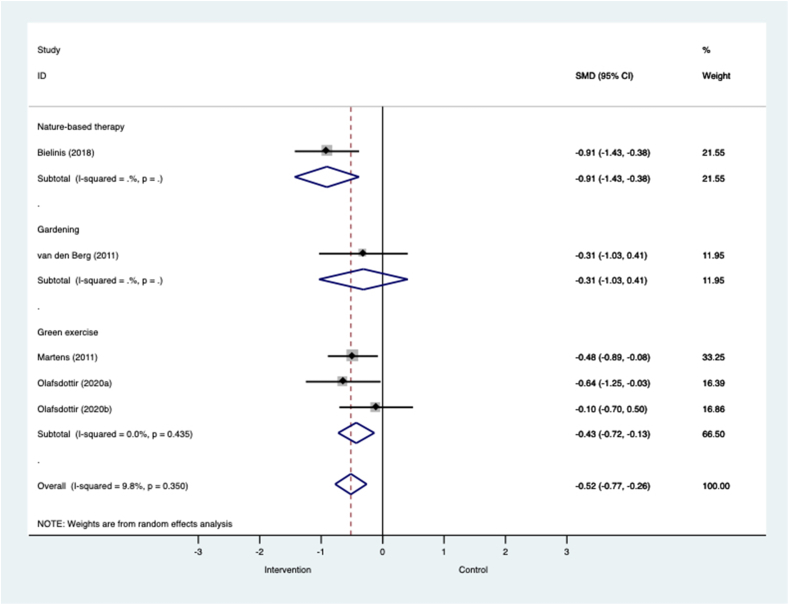
**Meta-analysis of RCTs of nature-based interventions for negative affect versus control at post-intervention across all populations** The size of the grey box reflects how much weight each study received in the meta-analysis (i.e., the larger the box the more this study contributed to the pooled effect represented by the blue diamond). Black bars represent the 95% CI for the SMD in each study. (For interpretation of the references to colour in this figure legend, the reader is referred to the Web version of this article.) CI = confidence interval; RCT = randomised controlled trial; SMD = standardised mean difference.

Fig. 5 shows that across four RCTs in all populations nature-based interventions were associated with moderate effects in favour of reducing negative affect (k=4; n=255; SMD=−0.52, −0.77 to −0.26; I^2^=9.8%, p=0.350). The largest effects were seen in one study of nature-based therapy in university student volunteers (k=1; n=62; SMD=−0.91, −1.43 to −0.38). Effects were small and non-significant in one trial that compared gardening with indoor reading among healthy volunteers (k=1; n=30; SMD=−0.31, −1.03 to 0.41). Green exercise interventions were associated with moderate and significant effects in favour of interventions. Tended forest walks were associated with stronger reductions in negative affect than walks in wild forests among university student volunteers (Martens et al., 2011). Green walks were associated with large effects when compared with indoor exercise, but the effect was small and non-significant when compared with watching nature on television (Olafsdottir et al., 2020).

Fig.C.7 shows that nature-based interventions across three controlled studies in all populations were associated with moderate but non-significant effects in favour of reducing negative affect (k=3; n=90; SMD=−0.60, −1.51 to 0.32; I^2^=76.0%, p=0.016). The most effective intervention in this pooled analysis was green exercise delivered in groups once a week for 3-weeks (de Brito et al., 2019). A 3-month gardening intervention in people with SMI favoured the intervention but effects were non-significant (Oh et al., 2018).

Only three single group before and after studies evaluated negative effect. Fig.C.8 shows that overall nature-based interventions were associated with moderate and significant effects in favour of reducing negative affect (k=3; n=175; SMD=−0.49, −0.70 to −0.27; I^2^=0.0%; p=0.511). While effects for green exercise were moderate and significant overall, a 13-week forest walk intervention in people with SMI was associated with non-significant effects (Iwata et al., 2016).

#### 3.3.5 Blood pressure

Three RCTs of green exercise and one RCT of gardening assessed the impact of interventions on blood pressure. Overall Fig.C.9 and Fig.C.10 respectively show that across all trials and all populations interventions were not associated with significant effects in favour of lowering either systolic (k=4; n=221; SMD=0.06, −0.20 to 0.33; I^2^=0.0%, p=0.424) or diastolic blood pressure (k=4; n=221; SMD=−0.09, −0.92 to 0.74; I^2^=82%, p=0.001. Populations were all healthy volunteers except for the RCT of gardening which included people with long-term conditions (Brown et al., 2020).

Two controlled studies of green exercise and one controlled study of gardening assessed the impact of interventions on blood pressure. Overall Fig.C.11 and Fig.C.12 respectively show that across all studies and all populations interventions were not associated with significant effects in favour of lowering either systolic (k=3; n = 197; SMD=−0.01, −0.45 to 0.42; I^2^=57.7%, p=0.069) or diastolic blood pressure (k=3; n=197; SMD=0.22, −0.03 to 0.48; I^2^=0.0%, p=0.911). One study that tested a 12 week group gardening intervention aimed to improve blood pressure in older adult women through low-to-moderate physical activity; it reported significantly lower systolic but not diastolic blood pressure (Sin-Ae et al., 2017).

#### 3.3.6 Blood lipids

One RCT (Müller-Riemenschneider et al., 2020) that compared a 6-month green exercise intervention with standard physical activity in healthy volunteers showed that the intervention did not significantly lower low-density lipoprotein (LDL) (k=1; n=145; SMD=0.34, 0.01 to 0.67) or improve high-density-lipoprotein (HDL) (k=1; n=145; SMD=0.02, −0.30 to 0.35). Similarly a controlled study of a forest walk compared with daily routine in university student volunteers did not report significant effects on either LDL or HDL (Bang et al., 2017). A controlled trial study compared a 3-month gardening intervention in older adults with treatment as usual also did not improve significantly either LDL or HDL (Sin-Ae et al., 2017).

#### 3.3.7 Physical activity

One RCT that tested a 1-year individual gardening intervention (k=1; N=78; SMD=0.51, 0.06 TO 0.97) and one controlled study that tested a 7.5 week group gardening intervention delivered twice a week for 50 min (k=1; n=50; SMD=1.19, 0.59 to 1.80) reported significant increases in physical activity. By contrast one RCT (k=1; n=145; SMD=0.19, −0.14 to 0.51) and one controlled study (k=1; n=118; SMD=0.12, −0.24 to 0.48) of green exercise interventions did not show significant increases in physical activity. A single group before and after study of nature-based therapy did report significant effects in favour of increased physical activity (k=1; n=77; SMD=0.38, 0.06 to 0.69).

## 4 Discussion

This systematic review and meta-analysis shows that outdoor nature-based interventions improve mental health outcomes across all populations, including older adults with long-term conditions and people with common mental health problems and SMI, as well as healthy adults. In this sense nature-based interventions are efficacious as both a therapeutic response to manage pre-existing mental health problems, and as a preventive approach to keep people well. We observed that nature-based therapies that were delivered in groups were associated with the largest and most consistent effects across mental health outcomes and across all pooled analyses, but there were insufficient studies per sub-group to formally test this. Forest therapies and wilderness therapies with an emphasis on immersion in nature for between four and eight weeks reduced depressive mood, anxiety symptoms, and negative affect, and enhanced positive affect. Large effects were also observed for gardening interventions, especially for interventions delivered in groups and that lasted for 12 weeks or more. Gardening reduced depressive mood in people with long-term health problems and also in people with common mental health problems and SMI. Similarly, gardening reduced anxiety symptoms and enhanced positive affect, but findings were more equivocal for negative affect, especially for people with SMI. Green exercise was also associated with moderate to large effects in favour of reducing depressive symptoms; group or individually delivered interventions that lasted between eight and 12 weeks were the most effective. The same pattern of results was returned for positive affect, although effects were attenuated in people with SMI. Green exercise delivered in various formats was also associated with reductions in anxiety, but findings were more mixed and only of borderline significance. The effect of green exercise on negative affect was less clear, with only one uncontrolled study of a 13-week programme reporting significant effects in favour of the intervention.

There was less evidence that nature-based interventions positively impact physical health, although there was a signal that gardening and nature-based therapies might increase physical activity. One-off experimental green exercise interventions tended to be associated with increased blood pressure, but this might reflect the timing of assessments taken immediately after exercise sessions. We did not identify any studies that assessed activities of daily living, functioning, or disability.

Compared with just exposure to green space, nature-based interventions offer opportunities to variously connect with nature, derive social support, and engage in physical and/or purposeful activity, and these factors are hypothesised to be potential mechanisms that underpin observed health benefits. The majority of gardening interventions included in this review were delivered in groups. Gardening undertaken in community groups is significantly associated with higher levels of nature connection and subjective wellbeing than observed in individual or home gardeners and non-gardeners (Koay et al., 2020). Nature connectedness concerns an individual's trait level of emotional connection with the natural world and individuals with greater nature connectedness have greater sense of eudaimonic wellbeing (Pritchard et al., 2020). It is evident that having an affective relationship with nature through engaging in outdoor activities is an important mechanism on the pathway to wellbeing benefits over and above benefits that might accrue from visiting or being exposed to nature alone (Richardson et al., 2018). Findings from this review tend to support this idea given that mental health gains among those who participated in nature-based interventions exceeded the effects observed in control groups, some of which included inactive exposure to green spaces. Furthermore, eudaimonic wellbeing is associated with personal growth and purposeful behaviour and gardening has been identified as an activity that can confer a sense of purpose and meaning to participants (Siu et al., 2020). Purposeful activity is also critical to social connectedness and is additionally associated with structural and social capital – key resources of wellbeing and lower levels of depression (Forsman et al., 2011). Social contact and personal achievement have been identified as positive experiences among those who take part in conservation activities (Husk et al., 2016), which might account for the positive health gains that we observed for conservation interventions.

The gains in mental health outcomes associated with green exercise and to a lesser extent gardening might also be linked to the antidepressive and anxiolytic effects of physical activity (Kvam et al., 2016; Stubbs et al., 2017). Previously it has been shown that taking exercise outdoors is associated with higher wellbeing, and lower feelings of stress and anxiety compared with similar exercise indoors, suggesting there is added value to outdoor physical activity in nature (Thompson Coon et al., 2011). It has been suggested that the additional mental health gains associated with outdoor green exercise might be partly attributed to the restorative qualities of green and blue spaces. This idea hinges on attention restoration theory (ART) which proposes that directed attention is associated with neurocognitive inhibitory mechanisms that supress distracting stimuli, leading to cognitive fatigue. Attention fatigue has been implicated in poorer decision making, stress and less self-control, leading to physical and mental ill health. ART proposes that directed attention is associated with neurocognitive inhibitory mechanisms that supress distracting stimuli, leading to cognitive fatigue and increased susceptibility to stress. Restoration of attentional capacity can occur in the presence of natural environments that offer intrinsically interesting aspects that promote involuntary attention and recovery from cognitive fatigue (Kaplan, 1995). Time spent ‘being away’ in natural environments that afford opportunities to engage in activities that are ‘softly fascinating’ have also been implicated in the health benefits attributed to more immersive activities such as forest bathing. However there is emerging evidence that forest bathing can improve mental health outcomes through activation of our parasympathetic nervous system that stimulates a psychophysiological stress recovery response (e.g. lowered blood pressure) owing to the hypothesis that humans have an innate preference for nature and natural systems (Kotera et al., 2020). This idea is central to stress reduction theory (Ulrich et al., 1991), but none of the included studies in this review that tested forest bathing or wilderness therapy measured impacts on physiological functions such as blood pressure.

Regardless of the mechanism of action we also identified that the most effective interventions were typically offered for between 8 and 12 weeks, and the optimal dose ranged from 20 to 90 min. This has practical implications given that in the UK nature-based interventions that are offered through social prescribing are most commonly delivered for 12 weeks (Bragg et al., 2017) but could possibly be offered for shorter periods of time. Improvements in mood in people with dementia have been observed following exposure to nature gardens for only 20 min' duration and the greatest benefits are associated with an outdoor exposure to nature of 80–90 min’ duration (White et al., 2018). Spending 120 min a week in nature has also been associated at population level with good health and high wellbeing (White et al., 2019).

### 4.1 Strengths and limitations

A strength of this systematic review is that it attempted to capture and quantitatively synthesise the totality of controlled and uncontrolled evidence of the health benefits of nature-based interventions across all populations, including those with mental health problems and long-term conditions. In this sense our review goes beyond the more narrow conceptual remit of previous reviews, including a recent review by Corazon et al. that included 36 studies of outdoor nature-based interventions, but was focused on stress recovery alone (Corazon et al., 2019). In addition to an inclusive approach to eligibility and outcomes we also adopted a broad approach to meta-analysis. By undertaking meta-analysis across interventions and across populations our review offered increased power to detect effects, reduced the risk of erroneous conclusions, and afforded opportunities for discussion about underlying hypotheses that might explain the findings. In line with this approach we strongly favoured an approach to produce summary estimates of interventions effects, as high heterogeneity alone is insufficient cause to preclude meta-analysis in this context (Gøtzsche, 2000). Furthermore, while we detected high levels of heterogeneity, these findings are positive as heterogeneity is consistently underestimated in meta-analyses (Kontopantelis et al., 2013). We were able to assess intervention effects across super-ordinate categories and in some instances the total I^2^ was reduced in these sub-group analyses. In these ways our review included the means to make meaningful summary estimates of intervention effects that could be of use to decision makers with responsibilities for commissioning nature-based interventions. However there were insufficient studies to permit analysis by population subgroup to determine which populations benefited the most from participation in nature-based interventions. Additionally, there were few too studies in each meta-analysis to undertake an assessment of publication bias and as such we cannot discount so-called ‘small study effects’. There is some evidence that ecological quality, in terms of biodiversity, is associated with the restorative benefits of urban greenspace, but we were unable to extract data about the type and quality of greenspace. Despite using an extensive and inclusive search strategy our review still under-represented studies that assessed nature-based interventions undertaken in blue spaces. There is emerging evidence that connectedness to blue space is advantageous for health and wellbeing and there is a case for a separate assessment of the health benefits of activities undertaken in blue spaces, especially in relation to the coast (White et al., 2014).

From a population health perspective we would ideally want to understand the health benefits of nature-based interventions over longer time horizons but the majority of studies included in this review only measured short term benefits at intervention end. Finally, while overall risk of bias ratings were low to moderate for RCTs the risk of bias was generally unclear for random sequence generation which can potentially lead to over estimates of treatment effects (Savović et al., 2017). The risk of bias for controlled studies and single group before and after studies was moderate to high and in this sense the findings across these studies should be judged with some caution. There is scope for future RCTs of nature-based interventions that include the use of validated mental and physical health outcomes and test codified and well described interventions with appropriate controls in defined populations with or without pre-existing health problems.

## 5 Conclusion

This broad and inclusive systematic review aimed to identify and synthesise evidence from controlled and uncontrolled studies about the effectiveness of nature-based interventions for mental and physical health among community based adult populations. Our review shows that outdoor nature-based interventions improve mental health outcomes in adult populations in the community, including those with common mental health problems, SMI, and long-term conditions. Nature-based therapies, such as forest bathing, were consistently effective across all mental health outcomes, although evidence from RCTs was limited. Group gardening and green exercise interventions were also effective for improving mental health outcomes, although effects were less strong for negative affect, especially in populations with SMI. We found less evidence that nature-based interventions improved physical health but there is potential for green exercise and gardening to increase physical activity.

The largest treatment effects were observed in studies that tested nature-based interventions for between eight and 12 weeks, with between 20 and 90 min of contact time per session. This has practical implications for scale up of nature-based interventions as delivery can more readily fit with existing provider delivery models and people can gain health benefits from modest amounts of regular engagement with nature. Gains in mental health might be attributed to nature connectedness, social support, physical activity and purposeful behaviour, but further research should address active ingredients of nature-based interventions through process evaluation of experimental controlled studies. In conclusion, nature-based interventions can effectively improve mental health and wellbeing. There is a need for substantial and sustained investment in community and place-based solutions such as nature-based interventions which are likely to play important role in addressing a post-pandemic surge in demand for mental health support.

## Funding

This study was part funded by the 10.13039/100014013UK Research and Innovation Closing the Gap Network+ (ES/S004459/1). UKRI does not necessarily endorse the views expressed by the authors. PAC, SG, and RM are part funded by the 10.13039/501100000272National Institute for Health Research (NIHR) Yorkshire and Humber Applied Research Collaboration https://www.arc-yh.nihr.ac.uk/. The views expressed are those of the author(s), and not necessarily those of the NIHR or the Department of Health and Social Care.

## Ethics statement

Analysis for this systematic review is based on published journal articles, and does not constitute human subjects research. No ethics and research governance approvals were required.

## Declaration of competing interest

We can confirm that no authors have any conflicts of interest to declare.
