# Supplementary material for: Nature-based outdoor activities for mental and physical health: Systematic review and meta-analysis
Source: SSM Popul Health. 2021 Oct 1;16:100934. doi: 10.1016/j.ssmph.2021.100934 (PMC8498096; doi:10.1016/j.ssmph.2021.100934)
Supplement: Multimedia component 1 [file mmc1.docx]

**ASSIA, Via Proquest; search date=9^th^ October 2019**

| Set# | Searched for |
| --- | --- |
| S1 | (ti(((conservation* AND natural AND environment* AND (renewal OR volunteer* OR voluntary OR participat* OR practical OR regenerat* OR restor* OR maintain* OR care OR enhance* OR preserve OR creat* OR activ* OR action* OR involve*)))) OR ab(((conservation* AND natural AND environment* AND (renewal OR volunteer* OR voluntary OR participat* OR practical OR regenerat* OR restor* OR maintain* OR care OR enhance* OR preserve OR creat* OR activ* OR action* OR involve*))))) AND la.exact("English") AND pd(19990101-20191009) |
| S2 | (ti(((Conservation NEAR/3 interventions))) OR ab(((Conservation NEAR/3 interventions)))) AND la.exact("English") AND pd(19990101-20191009) |
| S3 | ((ti((((environmental* NEAR/3 (conservation* OR volunteer* OR steward*)) AND (Regenerat* OR restore OR restoration OR redevelop OR maintain OR enhance OR preserve OR preserving OR create OR creation OR establish OR establishing OR founding OR build* OR cultivat* OR cultivation OR participati* OR practical OR creat* OR activ* OR action* OR involve*)))) OR ab((((environmental* NEAR/3 (conservation* OR volunteer* OR steward*)) AND (Regenerat* OR restore OR restoration OR redevelop OR maintain OR enhance OR preserve OR preserving OR create OR creation OR establish OR establishing OR founding OR build* OR cultivat* OR cultivation OR participati* OR practical OR creat* OR activ* OR action* OR involve*))))) AND la.exact("English") AND pd(20100101-20191009)) OR ((ti((((conservation* NEAR/3 (group* OR volunteer* OR voluntary OR association* OR organisation* OR organization* OR participa* OR stakeholder* OR steward* OR trust OR ranger* OR activit*)) AND (Regenerat* OR restore OR restoration OR redevelop OR maintain OR enhance OR preserve OR preserving OR create OR creation OR establish OR establishing OR founding OR build* OR cultivat* OR cultivation OR participati* OR practical OR creat* OR activ* OR action* OR involve*)))) OR ab((((conservation* NEAR/3 (group* OR volunteer* OR voluntary OR association* OR organisation* OR organization* OR participa* OR stakeholder* OR steward* OR trust OR ranger* OR activit*)) AND (Regenerat* OR restore OR restoration OR redevelop OR maintain OR enhance OR preserve OR preserving OR create OR creation OR establish OR establishing OR founding OR build* OR cultivat* OR cultivation OR participati* OR practical OR creat* OR activ* OR action* OR involve*))))) AND la.exact("English") AND pd(20100101-20191009)) |
| S4 | ((ti((nature OR rural OR countryside OR outdoor* OR outside OR backcountry OR hinterland OR outback OR wood* OR park* OR parkland OR garden* OR meadow* OR farm* OR (farm NEAR/1 land) OR horticultural OR floricultural OR botanical OR arboretum OR allotment* OR forest* OR rainforest OR moor* OR dale* OR marsh* OR mountain* OR beach* OR wilderness OR landscape* OR tree* OR copse* OR river* OR lake* OR canal* OR waterway OR wetland* OR (open NEAR/1 space*) OR (protected NEAR/1 area*) OR green* OR planning* OR footpath* OR trail* OR coast* OR cliff* OR dune* OR (bio NEAR/1 diversity) OR (eco NEAR/1 system) OR (protected NEAR/1 area*))) OR ab((nature OR rural OR countryside OR outdoor* OR outside OR backcountry OR hinterland OR outback OR wood* OR park* OR parkland OR garden* OR meadow* OR farm* OR (farm NEAR/1 land) OR horticultural OR floricultural OR botanical OR arboretum OR allotment* OR forest* OR rainforest OR moor* OR dale* OR marsh* OR mountain* OR beach* OR wilderness OR landscape* OR tree* OR copse* OR river* OR lake* OR canal* OR waterway OR wetland* OR (open NEAR/1 space*) OR (protected NEAR/1 area*) OR green* OR planning* OR footpath* OR trail* OR coast* OR cliff* OR dune* OR (bio NEAR/1 diversity) OR (eco NEAR/1 system) OR (protected NEAR/1 area*)))) AND pd(20100101-20191009)) AND (mainsubject("Conservation") AND pd(20100101-20191009)) |
| S5 | (ti((((((voluntary OR volunteer*) NEAR/5 (group* OR association OR stakeholder* OR steward* OR ranger*)) AND (environment* OR nature OR rural OR outdoor* OR outside OR (open NEAR/1 space*) OR conservation* OR wood* OR park* OR parkland OR garden* OR backcountry OR hinterland OR horticultural OR allotment* OR landscape OR scenic OR Botanical OR Arboretum OR forest* OR moor OR dale OR marsh* OR mountain* OR beach* OR wilderness OR wild OR tree* OR river* OR lake* OR canal* OR water OR waterway OR wetland* OR (open NEAR/1 space*) OR green* OR footpath OR trail)) AND (Regenerat* OR restore OR restoration OR redevelop OR maintain OR enhance OR preserve OR preserving OR create OR creation OR establish OR establishing OR founding OR build* OR cultivat* OR cultivation OR participati* OR practical OR creat* OR activ* OR action* OR involve*)))) OR ab((((((voluntary OR volunteer*) NEAR/5 (group* OR association OR stakeholder* OR steward* OR ranger*)) AND (environment* OR nature OR rural OR outdoor* OR outside OR (open NEAR/1 space*) OR conservation* OR wood* OR park* OR parkland OR garden* OR backcountry OR hinterland OR horticultural OR allotment* OR landscape OR scenic OR Botanical OR Arboretum OR forest* OR moor OR dale OR marsh* OR mountain* OR beach* OR wilderness OR wild OR tree* OR river* OR lake* OR canal* OR water OR waterway OR wetland* OR (open NEAR/1 space*) OR green* OR footpath OR trail)) AND (Regenerat* OR restore OR restoration OR redevelop OR maintain OR enhance OR preserve OR preserving OR create OR creation OR establish OR establishing OR founding OR build* OR cultivat* OR cultivation OR participati* OR practical OR creat* OR activ* OR action* OR involve*))))) AND la.exact("English") AND pd(20100101-20191009) |
| S6 | (ti(((Green* NEAR/3 (space* OR gym OR exercise OR volunteer* OR voluntary OR conservation OR infrastructure OR care OR streets OR communal OR Guerrilla)))) OR ab(((Green* NEAR/3 (space* OR gym OR exercise OR volunteer* OR voluntary OR conservation OR infrastructure OR care OR streets OR communal OR Guerrilla))))) AND la.exact("English") AND pd(20100101-20191009) |
| S7 | (ti((((conservation* NEAR/3 (group* OR volunteer* OR voluntary OR association* OR organisation* OR organization* OR participa* OR stakeholder* OR steward* OR trust OR ranger* OR activit*)) AND (Regenerat* OR restore OR restoration OR redevelop OR maintain OR enhance OR preserve OR preserving OR create OR creation OR establish OR establishing OR founding OR build* OR cultivat* OR cultivation OR participati* OR practical OR creat* OR activ* OR action* OR involve*)))) OR ab((((conservation* NEAR/3 (group* OR volunteer* OR voluntary OR association* OR organisation* OR organization* OR participa* OR stakeholder* OR steward* OR trust OR ranger* OR activit*)) AND (Regenerat* OR restore OR restoration OR redevelop OR maintain OR enhance OR preserve OR preserving OR create OR creation OR establish OR establishing OR founding OR build* OR cultivat* OR cultivation OR participati* OR practical OR creat* OR activ* OR action* OR involve*))))) AND la.exact("English") AND pd(201001-20191009) |
| S8 | (ti((((urban NEAR/3 (green* OR park* OR parkland OR garden* OR horticultur* OR wood* OR forest* OR botanical OR arboretum OR allotment* OR (open NEAR/1 space))) AND (Regenerat* OR restore OR restoration OR redevelop OR maintain OR enhance OR preserve OR preserving OR create OR creation OR establish OR establishing OR founding OR build* OR cultivat* OR cultivation OR participati* OR practical OR creat* OR activ* OR action* OR involve*)))) OR ab((((urban NEAR/3 (green* OR park* OR parkland OR garden* OR horticultur* OR wood* OR forest* OR botanical OR arboretum OR allotment* OR (open NEAR/1 space))) AND (Regenerat* OR restore OR restoration OR redevelop OR maintain OR enhance OR preserve OR preserving OR create OR creation OR establish OR establishing OR founding OR build* OR cultivat* OR cultivation OR participati* OR practical OR creat* OR activ* OR action* OR involve*))))) AND la.exact("English") AND pd(20100101-20191009) |
| S9 | (ti((greenspace)) OR ab((greenspace))) AND la.exact("English") AND pd(20100101-20191009) |
| S10 | (ti((((work* OR renewal OR volunteer* OR voluntary OR practical OR regenerat* OR restor* OR maintain* OR care OR enhance OR preserve OR creat*) AND (urban OR city OR metropolis OR town*) AND (garden* OR park* OR parkland OR allotment*)))) OR ab((((work* OR renewal OR volunteer* OR voluntary OR practical OR regenerat* OR restor* OR maintain* OR care OR enhance OR preserve OR creat*) AND (urban OR city OR metropolis OR town*) AND (garden* OR park* OR parkland OR allotment*))))) AND la.exact("English") AND pd(20100101-20191009) |
| S11 | ((ti((((garden* OR horticulture OR allotment* OR botanical OR arboretum) NEAR/5 (kitchen OR school* OR college* OR university OR campus OR hospital* OR prison* OR penitentiary OR institution OR urban OR green* OR communit* OR communal OR group* OR guerrilla OR (bio NEAR/1 diver*) OR eco)))) OR ab((((garden* OR horticulture OR allotment* OR botanical OR arboretum) NEAR/5 (kitchen OR school* OR college* OR university OR campus OR hospital* OR prison* OR penitentiary OR institution OR urban OR green* OR communit* OR communal OR group* OR guerrilla OR (bio NEAR/1 diver*) OR eco))))) AND la.exact("English") AND pd(20100101-20191009)) OR ((ti((((garden* OR horticulture OR allotment* OR botanical OR arboretum) AND (grow AND (your own))))) OR ab((((garden* OR horticulture OR allotment* OR botanical OR arboretum) AND (grow AND (your own)))))) AND la.exact("English") AND pd(20100101-20191009)) OR ((ti((((garden* OR horticulture OR allotment* OR botanical OR arboretum) NEAR/5 (renew* OR maintain* OR creat* OR culivat* OR enhance* OR restore OR regenerat* OR activ* OR preserve OR voluntary OR volunteer OR conservation* OR participat*)))) OR ab((((garden* OR horticulture OR allotment* OR botanical OR arboretum) NEAR/5 (renew* OR maintain* OR creat* OR culivat* OR enhance* OR restore OR regenerat* OR activ* OR preserve OR voluntary OR volunteer OR conservation* OR participat*))))) AND la.exact("English") AND pd(20100101-20191009)) OR ((ti((((garden* OR horticulture OR allotment* OR botanical OR arboretum) AND (pick AND (your own))))) OR ab((((garden* OR horticulture OR allotment* OR botanical OR arboretum) AND (pick AND (your own)))))) AND la.exact("English") AND pd(20100101-20191009)) AND pd(20100101-20191009) |
| S12 | ((ti(((((communit* NEAR/5 (group* OR team* OR association* OR organisation OR organization OR participa* OR stakeholder* OR steward* OR trust* OR ranger* OR activit*)) AND (garden* OR allotment* OR forest OR (natural AND environment) OR conservation*)) AND (Regenerat* OR restore OR restoration OR redevelop OR maintain OR enhance OR preserve OR preserving OR create OR creation OR establish OR establishing OR founding OR build* OR cultivat* OR cultivation OR participati* OR practical OR creat* OR activ* OR action* OR involve*)))) OR ab(((((communit* NEAR/5 (group* OR team* OR association* OR organisation OR organization OR participa* OR stakeholder* OR steward* OR trust* OR ranger* OR activit*)) AND (garden* OR allotment* OR forest OR (natural AND environment) OR conservation*)) AND (Regenerat* OR restore OR restoration OR redevelop OR maintain OR enhance OR preserve OR preserving OR create OR creation OR establish OR establishing OR founding OR build* OR cultivat* OR cultivation OR participati* OR practical OR creat* OR activ* OR action* OR involve*))))) AND la.exact("English") AND pd(20100101-20191009)) OR ((ti((((communit* AND (work* OR renewal OR volunteer* OR voluntary OR practical OR regenerat* OR restor* OR maintain* OR care OR enhance* OR preserve OR creat* OR activ* OR action* OR involve*) AND ((natur* adj3 environment*) OR (environmental* AND conservation*))) AND (Regenerat* OR restore OR restoration OR redevelop OR maintain OR enhance OR preserve OR preserving OR create OR creation OR establish OR establishing OR founding OR build* OR cultivat* OR cultivation OR participati* OR practical OR creat* OR activ* OR action* OR involve*)))) OR ab((((communit* AND (work* OR renewal OR volunteer* OR voluntary OR practical OR regenerat* OR restor* OR maintain* OR care OR enhance* OR preserve OR creat* OR activ* OR action* OR involve*) AND ((natur* adj3 environment*) OR (environmental* AND conservation*))) AND (Regenerat* OR restore OR restoration OR redevelop OR maintain OR enhance OR preserve OR preserving OR create OR creation OR establish OR establishing OR founding OR build* OR cultivat* OR cultivation OR participati* OR practical OR creat* OR activ* OR action* OR involve*))))) AND la.exact("English") AND pd(20100101-20191009)) OR ((ti(((((communit* OR local) NEAR/5 (garden* OR park* OR green* OR greenspace OR outdoor* OR outside* OR pavement* OR sidewalk* OR wood* OR allotment* OR lake* OR canal* OR river*)) AND (work* OR renewal OR volunteer* OR voluntary OR practical OR participat* OR regenerat* OR restor* OR maintain* OR enhance OR preserve OR creat*)))) OR ab(((((communit* OR local) NEAR/5 (garden* OR park* OR green* OR greenspace OR outdoor* OR outside* OR pavement* OR sidewalk* OR wood* OR allotment* OR lake* OR canal* OR river*)) AND (work* OR renewal OR volunteer* OR voluntary OR practical OR participat* OR regenerat* OR restor* OR maintain* OR enhance OR preserve OR creat*))))) AND la.exact("English") AND pd(20100101-20191009)) AND pd(20100101-20191009) |
| S13 | (ti(((((Volunteer* OR voluntary) NEAR/5 (environment* OR nature OR rural OR countryside OR outdoor* OR outside OR backcountry OR hinterland OR outback OR wood* OR park* OR parkland OR garden* OR meadow* OR horticultural OR floricultural OR botanical OR arboretum OR allotment* OR forest* OR moor* OR dale* OR marsh* OR mountain* OR beach* OR wilderness OR landscape* OR tree* OR copse* OR river* OR lake* OR canal* OR waterway OR wetland* OR (open NEAR/1 space*) OR green* OR planning* OR footpath OR trail OR (bio NEAR/1 diversity))) AND (Regenerat* OR restore OR restoration OR redevelop OR maintain OR enhance OR preserve OR preserving OR create OR creation OR establish OR establishing OR founding OR build* OR cultivat* OR cultivation OR participati* OR practical OR creat* OR activ* OR action* OR involve*)))) OR ab(((((Volunteer* OR voluntary) NEAR/5 (environment* OR nature OR rural OR countryside OR outdoor* OR outside OR backcountry OR hinterland OR outback OR wood* OR park* OR parkland OR garden* OR meadow* OR horticultural OR floricultural OR botanical OR arboretum OR allotment* OR forest* OR moor* OR dale* OR marsh* OR mountain* OR beach* OR wilderness OR landscape* OR tree* OR copse* OR river* OR lake* OR canal* OR waterway OR wetland* OR (open NEAR/1 space*) OR green* OR planning* OR footpath OR trail OR (bio NEAR/1 diversity))) AND (Regenerat* OR restore OR restoration OR redevelop OR maintain OR enhance OR preserve OR preserving OR create OR creation OR establish OR establishing OR founding OR build* OR cultivat* OR cultivation OR participati* OR practical OR creat* OR activ* OR action* OR involve*))))) AND pd(20100101-20191009) |
| S14 | ((ti((((conservation* NEAR/5 (nature OR rural OR countryside OR outdoor* OR outside OR backcountry OR hinterland OR outback OR wood* OR park* OR parkland OR garden* OR meadow* OR farm* OR (farm NEAR/1 land) OR horticultural OR floricultural OR botanical OR arboretum OR allotment* OR forest* OR rainforest OR moor* OR dale*1 OR marsh* OR mountain* OR beach* OR wilderness OR landscape* OR tree* OR copse* OR river* OR lake* OR canal* OR waterway OR wetland* OR (open NEAR/1 space*) OR (protected NEAR/1 area*) OR green* OR planning* OR footpath* OR trail* OR coast* OR cliff* OR dune* OR (bio NEAR/1 diversity) OR (eco NEAR/1 system) OR (protected NEAR/1 area*))) AND (Regenerat* OR restore OR restoration OR redevelop OR maintain OR enhance OR preserve OR preserving OR create OR creation OR establish OR establishing OR founding OR build* OR cultivat* OR cultivation OR participati* OR practical OR creat* OR activ* OR action* OR involve*)))) OR ab((((conservation* NEAR/5 (nature OR rural OR countryside OR outdoor* OR outside OR backcountry OR hinterland OR outback OR wood* OR park* OR parkland OR garden* OR meadow* OR farm* OR (farm NEAR/1 land) OR horticultural OR floricultural OR botanical OR arboretum OR allotment* OR forest* OR rainforest OR moor* OR dale*1 OR marsh* OR mountain* OR beach* OR wilderness OR landscape* OR tree* OR copse* OR river* OR lake* OR canal* OR waterway OR wetland* OR (open NEAR/1 space*) OR (protected NEAR/1 area*) OR green* OR planning* OR footpath* OR trail* OR coast* OR cliff* OR dune* OR (bio NEAR/1 diversity) OR (eco NEAR/1 system) OR (protected NEAR/1 area*))) AND (Regenerat* OR restore OR restoration OR redevelop OR maintain OR enhance OR preserve OR preserving OR create OR creation OR establish OR establishing OR founding OR build* OR cultivat* OR cultivation OR participati* OR practical OR creat* OR activ* OR action* OR involve*))))) AND la.exact("English") AND pd(2010101-20191009)) OR ((ti(((geoconservation OR (geo NEAR/3 conservation)))) OR ab(((geoconservation OR (geo NEAR/3 conservation))))) AND la.exact("English") AND pd(20100101-20191009)) OR ((ti((((activ* OR practical OR participat*) NEAR/3 conservation*))) OR ab((((activ* OR practical OR participat*) NEAR/3 conservation*)))) AND la.exact("English") AND pd(20100101-20191009)) AND pd(20100101-20191009) |

**Cochrane Central Register of Controlled Trials (CENTRAL), Via John Wiley’s Cochrane Library**

ID Search

#1 MeSH descriptor: [Environment] this term only

#2 MeSH descriptor: [Residence Characteristics] explode all trees

#3 MeSH descriptor: [Parks, Recreational] explode all trees

#4 MeSH descriptor: [Environment Design] explode all trees

#5 (green NEAR/2 (area* or cover or environment* or gym* or neighbourhood* or neighborhood* or roadside* or space*))

#6 ((city or cities or environment* or neighbourhood or neighborhood or urban) NEAR/2 greening)

#7 ((ambient or city or cities or environment* or neighbourhood or neighborhood or residential or surrounding or urban) NEAR/2 greenness)

#8 greenery or greenspace* or garden* or park or parks or parkland* or "sports field" or "wilderness area*" or "public open space*" or "neighbourhood open space*" or "neighborhood open space*"

#9 #1 or #2 or #3 or #4 or #5 or #6 or #7 or #8

#10 MeSH descriptor: [Water] this term only

#11 MeSH descriptor: [Rivers] this term only

#12 MeSH descriptor: [Oceans and Seas] this term only

#13 bluespace* or "blue space*" or bluehealth or "blue water*" or "blue gym*"

#14 #10 or #11 or #12 or #13

#15 MeSH descriptor: [Nature] this term only

#16 "health promot*" NEAR/2 environment*

#17 (natural or outdoor* or salutogenic) NEAR/2 environment*

#18 (nature or natural) NEAR/2 space*

#19 #15 or #16 or #17 or #18

#20 #9 or #14 or #19

#21 MeSH descriptor: [Mental Health] explode all trees

#22 mental near/2 (health or illness*)

#23 MeSH descriptor: [Depressive Disorder] explode all trees

#24 MeSH descriptor: [Depression] this term only

#25 depression or depressive

#26 (depressed or low) NEAR/2 mood

#27 MeSH descriptor: [Mood Disorders] this term only

#28 "affective disorder*"

#29 MeSH descriptor: [Anxiety Disorders] explode all trees

#30 anxiety or anxious

#31 MeSH descriptor: [Stress, Psychological] explode all trees

#32 "stressful life event*" or resilien*

#33 wellbeing or well-being or "well being"

#34 #21 or #22 or #23 or #24 or #25 or #26 or #27 or #28 or #29 or #30 or #31 or #32 or #33

#35 #20 and 34

#36 MeSH descriptor: [Cardiovascular Diseases] this term only

#37 MeSH descriptor: [Sleep] this term only

#38 MeSH descriptor: [Quality of Life] this term only

#39 MeSH descriptor: [Activities of Daily Living] this term only

#40 MeSH descriptor: [Hypertension] this term only

#41 MeSH descriptor: [Pain] this term only

#42 (enhanc* or improv* or better) NEAR/1 sleep

#43 (alleviat* or decreas* or lessen or manag* or reduc*) NEAR/2 pain

#44 (alleviat* or lessen or lower* or manag* or reduc*) NEAR/2 "blood pressure"

#45 (better or enhanc* or improv* or increas*) near/1 "quality of life"

#46 (alleviat* or lessen or manag* or reduc*) NEAR/2 disabilit*

#47 #36 or #37 or #38 or #39 or #40 or #41 or #42 or #43 or #44 or #45 or #46

#48 #20 and #47

#49 (greenspace* or green space* or greenery or greenness) NEAR/3 (health or wellbeing or stress or depress* or anxiety or mental or wellness)

#50 (allotment* or garden or gardens or park or parks or parkland*) NEAR/3 (health or wellbeing or stress or depress* or anxiety or mental or wellness)

#51 (bushland* or countryside or forest* or woodland*) NEAR/3 (health or wellbeing or stress or depress* or anxiety or mental or wellness)

#52 (bluespace* or blue space*) NEAR/3 (health or wellbeing or stress or depress* or anxiety or mental or wellness)

#53 (beach or beaches or coastal or coastline* or seaside) NEAR/3 (health or wellbeing or stress or depress* or anxiety)

#54 "marine area*" NEAR/3 (health or wellbeing or stress or depress* or anxiety or mental or wellness)

#55 "marine environment*" NEAR/3 (health or wellbeing or stress or depress* or anxiety or mental or wellness)

#56 "aquatic area*" NEAR/3 (health or wellbeing or stress or depress* or anxiety or mental or wellness)

#57 "acquatic environment*" NEAR/3 (health or wellbeing or stress or depress* or anxiety or mental or wellness)

#58 (canal or fountain* or lake or lakes or lakeside or riparian or river or stream) NEAR/3 (health or wellbeing or stress or depress* or anxiety or mental or wellness)

#59 ("water feature*" or waterfront* or waterscape* or waterway* or "inland water" or "inland surface water*" or "outdoor water environment*") NEAR/3 (health or wellbeing or stress or depress* or anxiety or mental or wellness)

#60 #49 or #50 or #51 or #52 or #53 or #54 or #55 or #56 or #57 or #58 or #59

#61 #35 or #48 or #60

#62 MeSH descriptor: [Physical Fitness] this term only

#63 MeSH descriptor: [Recreation] explode all trees

#64 MeSH descriptor: [Leisure Activities] this term only

#65 MeSH descriptor: [Exercise] explode all trees

#66 MeSH descriptor: [Exercise Therapy] explode all trees

#67 physical NEAR/3 (fit* or train* or activ* or endur*)

#68 exercis* NEAR/3 (fit* or train* or activ* or endur*)

#69 (promot* or uptak* or encourag* or increas* or start* or adher*) NEAR/3 (exercis* or sport* or fitness)

#70 MeSH descriptor: [Running] this term only

#71 MeSH descriptor: [Jogging] this term only

#72 MeSH descriptor: [Walking] this term only

#73 sport* or walk* or running or jogging or bicycling or biking or trekking or rambling or "forest bathing"

#74 MeSH descriptor: [Water Sports] explode all trees

#75 MeSH descriptor: [Swimming] this term only

#76 MeSH descriptor: [Diving] this term only

#77 boating or canoeing or diving or kayaking or rowing or surfboarding or swimming or "water skiing" or "water sport*"

#78 (craft or creative or leisure) NEAR/2 (activit* or task*)

#79 MeSH descriptor: [Gardening] explode all trees

#80 gardening

#81 #62 or #63 or #64 or #65 or #66 or #67 or #68 or #69 or #70 or #71 or #72 or #73 or #74 or #75 or #76 or #77 or #78 or #79 or #80

#82 #61 and #81 with Publication Year from 2010 to 2019, in Trials

#83 protocol:ti

#84 #82 not #83

**CENTRAL Search 2**

**Last Saved: 07/10/2019 16:44:03**

ID Search

#1 (conservation* and natural and environment* and (renewal or volunteer* or voluntary or participate* or practical or regenerate* or restor* or maintain* or care or enhance* or preserve or great* or activ* or action* or involve*))

#2 (Conservation near/3 interventions)

#3 ((environmental* near/3 (conservation* or volunteer* or steward*)) and (Regenerat* or restore or restoration or redevelop or maintain or enhance or preserve or preserving or create or creation or establish or establishing or founding or build* or cultivat* or cultivation or participate or participation))

#4 (conservation* near/3 (group* or volunteer* or voluntary or association* or organisation* or organization* or participa* or stakeholder* or steward* or trust or ranger* or activit*))

#5 (conservation* near/5 (nature or rural or countryside or outdoor* or outside or backcountry or hinterland or outback or wood* or park* or parkland or garden* or meadow* or farm* or (farm near/1 land) or horticultural or botanical or arboretum or allotment* or forest* or rainforest or moor* or dale* or marsh* or mountain* or beach* or wilderness or landscape* or tree* or copse* or river* or lake* or canal* or waterway or wetland* or (open near/1 space*) or (protected near/1 area*) or green* or planning* or footpath* or trail* or coast* or cliff* or dune* or (bio near/1 diversity) or (eco near/1 system) or (protected near/1 area*)))

#6 (geoconservation or (geo near/3 conservation))

#7 ((activ* or practical or participat*) near/3 conservation*)

#8 MeSH descriptor: [Conservation of Natural Resources] explode all trees

#9 #1 or #2 or #3 or #4 or #5 or #6 or #7 or #8

#10 ((Volunteer* or voluntary) near/5 (environment* or nature or rural or countryside or outdoor* or outside or backcountry or hinterland or outback or wood* or park* or parkland or garden* or meadow* or farm* or (farm near/1 land) or horticultural or botanical or arboretum or allotment* or forest* or rainforest or moor* or dale* or marsh* or mountain* or beach* or wilderness or landscape* or tree* or copse* or river* or lake* or canal* or waterway or wetland* or (open near/1 space*) or (protected near/1 area*) or green* or planning* or footpath* or trail* or coast* or cliff* or dune* or (bio near/1 diversity) or (eco near/1 system) or (protected near/1 area*)))

#11 (((voluntary or volunteer*) near/5 (group* or association or stakeholder* or steward* or ranger*)) and (environment* or nature or rural or countryside or outdoor* or outside or backcountry or hinterland or outback or wood* or park* or parkland or garden* or meadow* or farm* or (farm near/1 land) or horticultural or botanical or arboretum or allotment* or forest* or rainforest or moor* or dale* or marsh* or mountain* or beach* or wilderness or landscape* or tree* or copse* or river* or lake* or canal* or waterway or wetland* or (open near/1 space*) or (protected near/1 area*) or green* or planning* or footpath* or trail* or coast* or cliff* or dune* or (bio near/1 diversity) or (eco near/1 system) or (protected near/1 area*)))

#12 MeSH descriptor: [Volunteers] explode all trees

#13 (environment* or nature or rural or countryside or outdoor* or outside or backcountry or hinterland or outback or wood* or park* or parkland or garden* or meadow* or farm* or (farm near/1 land) or horticultural or botanical or arboretum or allotment* or forest* or rainforest or moor* or dale* or marsh* or mountain* or beach* or wilderness or landscape* or tree* or copse* or river* or lake* or canal* or waterway or wetland* or (open near/1 space*) or (protected near/1 area*) or green* or planning* or footpath* or trail* or coast* or cliff* or dune* or (bio near/1 diversity) or (eco near/1 system) or (protected near/1 area*))

#14 #12 and #13

#15 #10 or #11 or #14

#16 (Green* near/3 (space* or gym or exercise or volunteer* or voluntary or conservation or infrastructure or care or streets or communal or Guerrilla))

#17 (greenspace)

#18 #16 or #17

#19 (urban near/3 (green* or park* or parkland or garden* or horticultur* or wood* or forest* or botanical or arboretum or allotment* or (open near/1 space)))

#20 ((work* or renewal or volunteer* or voluntary or practical or regenerat* or restor* or maintain* or care or enhance or preserve or creat*) and (urban or city or metropolis or town*) and (garden* or park* or parkland or allotment*))

#21 MeSH descriptor: [Cities] explode all trees

#22 ((work* or renewal or volunteer* or voluntary or practical or regenerat* or restor* or maintain* or care or enhance or preserve or creat*) and (garden* or park* or parkland or allotment*))

#23 #21 or #22

#24 ((garden* or horticulture or allotment* or botanical or arboretum) near/5 (kitchen or school* or college* or university or campus or hospital* or prison* or penitentiary or institution or urban or green* or communit* or communal or group* or guerrilla or (bio near/1 diver*) or eco or ((grow or pick) near/3 your own)))

#25 ((garden* or horticulture or allotment* or botanical or arboretum) near/5 (maintain* or creat* or culivat* or enhance* or preserve or voluntary or volunteer or conservation* or participat*))

#26 MeSH descriptor: [Gardening] explode all trees

#27 #19 or #20 or #23 or #24 or #25 or #26

#28 ((communit* near/5 (group* or team* or association* or organisation or organization or participa* or stakeholder* or steward* or trust* or ranger* or activit*)) and (garden* or allotment* or forest or (natural and environment) or conservation*))

#29 (communit* and (work* or renewal or volunteer* or voluntary or practical or regenerat* or restor* or maintain* or care or enhance* or preserve or creat* or activ* or action* or involve*) and ((natur* near/3 environment*) or (environmental* and conservation*)))

#30 (((communit* or local) near/5 (garden* or park* or green* or greenspace or outdoor* or outside* or pavement* or sidewalk* or wood* or allotment* or lake* or canal* or river*)) and (work* or renewal or volunteer* or voluntary or practical or participat* or regenerat* or restor* or maintain* or enhance or preserve or creat*))

#31 #28 or #29 or #30

#32 #9 or #15 or #18 or #27 or #31

#33 (clinical or surgery or surgical or cell or cells or laboratory or placebo or bladder or uterus or breast or gene or genes or genetic or bowel or liver or enzymes or viral or lymph or molecular)

#34 #32 not #33 with Publication Year from 2010 to 2019, in Trials

#35 protocol:ti

#36 #34 not #35

**Embase, Via OVID; Search date=7^th^ October 2019**

**Database: Embase <1996 to 2019 Week 40>**

1 *Environment/ (15086)

2 *Landscape/ (4389)

3 *Rural Area/ or *Urban Area/ (8926)

4 *Recreational Park/ or *National Park/ (810)

5 *Wilderness/ (166)

6 *Agricultural Land/ (1626)

7 *"Land Use"/ (3262)

8 (green adj2 (area$ or cover or environment$ or gym$ or neighbourhood$ or neighborhood$ or roadside$ or space$)).ti,ab,kw. (2368)

9 ((city or cities or environment$ or neighbourhood or neighborhood or urban) adj2 greening).ti,ab,kw. (111)

10 ((ambient or city or cities or environment$ or neighbourhood or neighborhood or residential or surrounding or urban) adj2 greenness).ti,ab,kw. (166)

11 (greenery or greenspace$).ti,ab,kw. (373)

12 (garden$ or park$1 or parkland$).ti,ab,kw. (34953)

13 sports field$.ti,ab,kw. (113)

14 wilderness.ti,ab,kw. (1260)

15 public open space$.ti,ab,kw. (102)

16 neighbourhood open space$.ti,ab,kw. (1)

17 neighborhood open space$.ti,ab,kw. (2)

18 1 or 2 or 3 or 4 or 5 or 6 or 7 or 8 or 9 or 10 or 11 or 12 or 13 or 14 or 15 or 16 or 17 (70248)

19 *River/ (6431)

20 *Seashore/ (3238)

21 *Lake/ (2959)

22 *Aquatic Environment/ (2622)

23 *Ocean Environment/ (400)

24 *Freshwater Environment/ (796)

25 *Marine Environment/ (3851)

26 (bluespace$ or blue space$).ti,ab,kw. (93)

27 bluehealth.ti,ab,kw. (1)

28 blue water$.ti,ab,kw. (179)

29 blue gym$.ti,ab,kw. (3)

30 19 or 20 or 21 or 22 or 23 or 24 or 25 or 26 or 27 or 28 or 29 (20246)

31 (health promot$ adj2 environment$).ti,ab,kw. (237)

32 ((natural or outdoor$ or salutogenic) adj2 environment$).ti,ab,kw. (13651)

33 ((nature or natural) adj2 space$).ti,ab,kw. (338)

34 31 or 32 or 33 (14201)

35 18 or 30 or 34 (102894)

36 *Mental Health/ (27927)

37 (mental$ adj2 (health or ill$)).ti,ab,kw. (182458)

38 exp *Depression/ (154856)

39 (depression or depressive).ti,ab,kw. (422982)

40 ((depressed or low) adj2 mood).ti,ab,kw. (7111)

41 *Mood Disorder/ (8131)

42 affective disorder$.ti,ab,kw. (16877)

43 exp *Anxiety Disorder/ (85975)

44 (anxiety or anxious).ti,ab,kw. (241436)

45 exp *Psychological Stress/ (23179)

46 (stressful life event$ or resilien$).ti,ab,kw. (38198)

47 (wellbeing or well-being or well being).ti,ab,kw. (101534)

48 36 or 37 or 38 or 39 or 40 or 41 or 42 or 43 or 44 or 45 or 46 or 47 (855586)

49 35 and 48 (4114)

50 *Cardiovascular Disease/ (79192)

51 *Sleep/ (35448)

52 *"Quality of life"/ (93388)

53 *Daily Life Activity/ (10053)

54 *Hypertension/ (115674)

55 *Pain/ (64664)

56 ((enhanc$ or improv$ or better) adj sleep).ti,ab. (6511)

57 ((alleviat$ or decreas$ or lessen or manag$ or reduc$) adj2 pain).ti,ab. (91496)

58 ((alleviat$ or lessen or lower$ or manag$ or reduc$) adj2 blood pressure).ti,ab. (31485)

59 ((better or enhanc$ or improv$ or increas$) adj quality of life).ti,ab. (34672)

60 ((alleviat$ or lessen or manag$ or reduc$) adj2 disabilit$).ti,ab. (4620)

61 50 or 51 or 52 or 53 or 54 or 55 or 56 or 57 or 58 or 59 or 60 (521820)

62 35 and 61 (1215)

63 49 or 62 (5129)

64 *Fitness/ or *Physical Activity/ (44667)

65 exp *Recreation/ or *Sport/ (32374)

66 *Leisure Activities/ or *Leisure/ (5363)

67 exp *Exercise/ (102842)

68 exp *Exercise Therapy/ (24555)

69 (physical adj3 (fit$ or train$ or activ$ or endur$)).ti,ab. (152862)

70 (exercis$ adj3 (fit$ or train$ or activ$ or endur$)).ti,ab. (44607)

71 ((promot$ or uptak$ or encourag$ or increas$ or start$ or adher$) adj3 (exercis$ or sport$ or fitness)).ti,ab. (35709)

72 *Running/ or *Jogging/ or *Walking/ (20264)

73 (sport$ or walk$ or running or jogging or bicycling or biking or trekking or rambling or forest bathing).ti,ab. (268351)

74 *Aquatic sport/ or *Swimming/ or *Diving/ or *Water Skiing/ (7544)

75 (boating or canoeing or diving or kayaking or rowing or surfboarding or swimming or water skiing or water sport$).ti,ab,kw. (33253)

76 ((craft or creative or leisure) adj2 (activit$ or task$)).ti,ab. (6486)

77 *Gardening/ or gardening.ti,ab,kw. (1734)

78 64 or 65 or 66 or 67 or 68 or 69 or 70 or 71 or 72 or 73 or 74 or 75 or 76 or 77 (552981)

79 63 and 78 (1013)

80 (rat or rats or mouse or mice or swine or porcine or murine or sheep or lambs or pigs or piglets or rabbit or rabbits or cat or cats or dog or dogs or cattle or bovine or monkey or monkeys or trout or marmoset$1 or bird or birds or fish or fishes or insect or insects).ti. and animal experiment/ (667370)

81 Animal experiment/ not (human experiment/ or human/) (1383630)

82 80 or 81 (1421925)

83 79 not 82 (995)

84 ((greenspace$ or green space$ or greenery or greenness) adj3 (health or wellbeing or stress or depress$ or anxiety or mental or wellness)).ti,ab,kw. (182)

85 ((allotment$ or garden or gardens or park or parks or parkland$) adj3 (health or wellbeing or stress or depress$ or anxiety or mental or wellness)).ti,ab,kw. (340)

86 ((bushland$ or countryside or forest$ or woodland$) adj3 (health or wellbeing or stress or depress$ or anxiety or mental or wellness)).ti,ab,kw. (612)

87 ((bluespace$ or blue space$) adj3 (health or wellbeing or stress or depress$ or anxiety or mental or wellness)).ti,ab,kw. (23)

88 ((beach or beaches or coastal or coastline$ or seaside) adj3 (health or wellbeing or stress or depress$ or anxiety)).ti,ab,kw. (498)

89 (marine area$ adj3 (health or wellbeing or stress or depress$ or anxiety or mental or wellness)).ti,ab,kw. (0)

90 (marine environment$ adj3 (health or wellbeing or stress or depress$ or anxiety or mental or wellness)).ti,ab,kw. (78)

91 (aquatic area$ adj3 (health or wellbeing or stress or depress$ or anxiety or mental or wellness)).ti,ab,kw. (0)

92 (acquatic environment$ adj3 (health or wellbeing or stress or depress$ or anxiety or mental or wellness)).ti,ab,kw. (0)

93 ((canal or fountain$ or lake or lakes or lakeside or riparian or river or stream) adj3 (health or wellbeing or stress or depress$ or anxiety or mental or wellness)).ti,ab,kw. (787)

94 ((water feature$ or waterfront$ or waterscape$ or waterway$ or inland water or inland surface water$ or outdoor water environment$) adj3 (health or wellbeing or stress or depress$ or anxiety or mental or wellness)).ti,ab,kw. (17)

95 84 or 85 or 86 or 87 or 88 or 89 or 90 or 91 or 92 or 93 or 94 (2519)

96 limit 95 to human (1408)

97 78 and 96 (222)

98 83 or 97 (1116)

99 (conservation$ and natural and environment$ and (renewal or volunteer$ or voluntary or participat$ or practical or regenerat$ or restor$ or maintain$ or care or enhance$ or preserve or creat$ or activ$ or action$ or involve$)).ti,ab. (1076)

100 (Conservation adj3 interventions).ti,ab. (110)

101 ((environmental$ adj3 (conservation$ or volunteer$ or steward$)) and (Regenerat$ or restore or restoration or redevelop or maintain or enhance or preserve or preserving or create or creation or establish or establishing or founding or build$ or cultivat$ or cultivation or participate or participation)).ti,ab. (275)

102 (conservation$ adj3 (group$ or volunteer$ or voluntary or association$ or organisation$ or organization$ or participa$ or stakeholder$ or steward$ or trust or ranger$ or activit$)).ti,ab. (1357)

103 (conservation$ adj3 (nature or rural or countryside or outdoor$ or outside or backcountry or hinterland or outback or wood$ or park$1 or parkland or garden$ or meadow$ or farm$ or (farm adj1 land) or horticultural or botanical or arboretum or allotment$ or forest$ or rainforest or moor$ or dale$1 or marsh$ or mountain$ or beach$ or wilderness or landscape$ or tree$ or copse$ or river$ or lake$ or canal$ or waterway or wetland$ or (open adj1 space$) or (protected adj1 area$) or green$ or planning$ or footpath$ or trail$ or coast$ or cliff$ or dune$ or (bio adj1 diversity) or (eco adj1 system) or (protected adj1 area$))).ti,ab. (3735)

104 (geoconservation or (geo adj3 conservation)).ti,ab. (6)

105 ((activ$ or practical or participat$) adj3 conservation$).ti,ab. (878)

106 environmental protection/ (35502)

107 (volunteer$ or voluntary).ti,ab. or (*voluntary worker/ or *consumer/ or *health status/) (292873)

108 106 and 107 (404)

109 99 or 100 or 101 or 102 or 103 or 104 or 105 or 108 (6712)

110 ((Volunteer$ or voluntary) adj3 (environment$ or nature or rural or countryside or outdoor$ or outside or backcountry or hinterland or outback or wood$ or park$1 or parkland or garden$ or meadow$ or horticultural or botanical or arboretum or allotment$ or forest$ or rainforest or moor$ or dale$1 or marsh$ or mountain$ or beach$ or wilderness or landscape$ or tree$ or copse$ or river$ or lake$ or canal$ or waterway or wetland$ or (open adj1 space$) or (protected adj1 area$) or green$ or planning$ or footpath$ or trail$ or coast$ or cliff$ or dune$ or (bio adj1 diversity) or (eco adj1 system) or (protected adj1 area$))).ti,ab. (1021)

111 (((voluntary or volunteer$) adj3 (group$ or association or stakeholder$ or steward$ or ranger$)) and (environment$ or nature or rural or countryside or outdoor$ or outside or backcountry or hinterland or outback or wood$ or park$1 or parkland or garden$ or meadow$ or horticultural or botanical or arboretum or allotment$ or forest$ or rainforest or moor$ or dale$1 or marsh$ or mountain$ or beach$ or wilderness or landscape$ or tree$ or copse$ or river$ or lake$ or canal$ or waterway or wetland$ or (open adj1 space$) or (protected adj1 area$) or green$ or planning$ or footpath$ or trail$ or coast$ or cliff$ or dune$ or (bio adj1 diversity) or (eco adj1 system) or (protected adj1 area$))).ti,ab. (796)

112 *voluntary worker/ (1791)

113 (environment$ or nature or rural or countryside or outdoor$ or outside or backcountry or hinterland or outback or wood$ or park$1 or parkland or garden$ or meadow$ or horticultural or botanical or arboretum or allotment$ or forest$ or rainforest or moor$ or dale$1 or marsh$ or mountain$ or beach$ or wilderness or landscape$ or tree$ or copse$ or river$ or lake$ or canal$ or waterway or wetland$ or (open adj1 space$) or (protected adj1 area$) or green$ or planning$ or footpath$ or trail$ or coast$ or cliff$ or dune$ or (bio adj1 diversity) or (eco adj1 system) or (protected adj1 area$)).ti,ab. (2446116)

114 112 and 113 (159)

115 110 or 111 or 114 (1914)

116 (Green$ adj3 (space$ or gym or exercise or volunteer$ or voluntary or conservation or infrastructure or care or streets or communal or Guerrilla)).ti,ab. (1781)

117 greenspace.ti,ab. (174)

118 116 or 117 (1927)

119 (urban adj3 (green$ or park$1 or parkland or garden$ or horticultur$ or wood$ or forest$ or botanical or arboretum or allotment$ or (open adj1 space))).ti,ab. (2255)

120 ((work$ or renewal or volunteer$ or voluntary or practical or regenerat$ or restor$ or maintain$ or care or enhance or preserve or creat$) and (urban or city or metropolis or town$) and (garden$ or park$1 or parkland or allotment$)).ti,ab. (990)

121 *city/ and ((work$ or renewal or volunteer$ or voluntary or practical or regenerat$ or restor$ or maintain$ or care or enhance or preserve or creat$) and (garden$ or park$1 or parkland or allotment$)).ti,ab. (28)

122 *health/ and (environmental protection/ or voluntary worker/) (463)

123 119 or 120 or 121 or 122 (3523)

124 ((garden$ or horticulture or allotment$ or botanical or arboretum) adj3 (kitchen or school$ or college$ or university or campus or hospital$ or prison$ or penitentiary or institution or urban or green$ or communit$ or communal or group$ or guerrilla or (bio adj1 diver$) or eco or ((grow or pick) adj3 your own))).ti,ab. (1291)

125 ((garden$ or horticulture or allotment$ or botanical or arboretum) adj3 (maintain$ or creat$ or culivat$ or enhance$ or preserve or voluntary or volunteer or conservation$ or participat$)).ti,ab. (219)

126 *gardening/ and (environmental protection/ or voluntary worker/) (13)

127 *Gardening/ and (kitchen or school$ or college$ or university or campus or hospital$ or prison$ or penitentiary or institution or urban or green$ or communit$ or communal or group$ or guerrilla or (bio adj1 diver$) or eco or maintain$ or creat$ or culivat$ or voluntary or volunteer or conservation$ or participat$).ti,ab. (302)

128 124 or 125 or 126 or 127 (1578)

129 ((communit$ adj3 (group$ or team$ or association$ or organisation or organization or participa$ or stakeholder$ or steward$ or trust$ or ranger$ or activit$)) and (garden$ or allotment$ or forest or (natural and environment) or conservation$)).ti,ab. (727)

130 (communit$ and (work$ or renewal or volunteer$ or voluntary or practical or regenerat$ or restor$ or maintain$ or care or enhance$ or preserve or creat$ or activ$ or action$ or involve$) and ((natur$ adj3 environment$) or (environmental$ and conservation$))).ti,ab. (1474)

131 (((communit$ or local) adj3 (garden$ or park$1 or parkland$ or green$ or greenspace or outdoor$ or outside$ or pavement$ or sidewalk$ or wood$ or allotment$ or lake$ or canal$ or river$)) and (work$ or renewal or volunteer$ or voluntary or practical or participat$ or regenerat$ or restor$ or maintain$ or enhance or preserve or creat$)).ti,ab. (1432)

132 129 or 130 or 131 (3417)

133 109 or 115 or 118 or 123 or 128 or 132 (17285)

134 (clinical or surgery or surgical or cell or cells or laboratory or placebo or bladder or uterus or breast or gene or genes or genetic or bowel or liver or enzymes or viral or lymph or molecular).mp. (16208195)

135 (rat or rats or mouse or mice or swine or porcine or murine or sheep or lambs or pigs or piglets or rabbit or rabbits or cat or cats or dog or dogs or cattle or bovine or monkey or monkeys or trout or marmoset$1 or bird or birds or fish or fishes or insect or insects).ti. and animal experiment/ (667370)

136 Animal experiment/ not (human experiment/ or human/) (1383630)

137 134 or 135 or 136 (16547288)

138 133 not 137 (11169)

139 83 or 138 (11906)

140 limit 139 to (english language and embase and yr="2010 -Current") (4411)

**Greenfile Via Ebsco. Search date=7^th^ October**

**Search 1**

S23 S18 AND S22 (238)

S22 S19 OR S20

Limiters - Publication Date: 20100101-2019123 (9,587)

S21 S19 OR S20

S20 gardening

S19 TI ( “Physical Fitness” or “physical activit*” or recreation or leisure or exercise ) OR AB ( “Physical Fitness” or “physical activit*” or recreation or leisure or exercise ) OR TI ( physical N3 (fit* or train* or activ* or endur*) ) OR AB ( physical N3 (fit* or train* or activ* or endur*) ) OR TI ( exercis* N3 (fit* or train* or activ* or endur*) ) OR AB ( exercis* N3 (fit* or train* or activ* or endur*) ) OR TI ( ((promot* or uptak* or encourag* or increas* or start* or adher*) N3 (exercis* or sport* or fitness)) ) OR AB ( ((promot* or uptak* or encourag* or increas* or start* or adher*) N3 (exercis* or sport* or fitness)) ) OR TI ( (sport* or walk* or running or jogging or bicycling or biking or trekking or rambling or "forest bathing" or gardening) ) OR AB ( (sport* or walk* or running or jogging or bicycling or biking or trekking or rambling or "forest bathing" or gardening) ) OR TI ( (boating or canoeing or diving or kayaking or rowing or surfboarding or swimming or "water skiing" or "water sport*") ) OR AB ( (boating or canoeing or diving or kayaking or rowing or surfboarding or swimming or "water skiing" or "water sport*") )

S18 S16 OR S17

S17 ( ((greenspace* or "green space*" or greenery or greenness) N3 (health or wellbeing or stress or depress* or anxiety or mental or wellness)) ) OR ( ((allotment* or garden or gardens or park or parks or parkland*) N3 (health or wellbeing or stress or depress* or anxiety or mental or wellness)) ) OR ( ((bushland* or countryside or forest* or woodland*) N3 (health or wellbeing or stress or depress* or anxiety or mental or wellness)) ) OR ( ((bluespace* or "blue space*") N3 (health or wellbeing or stress or depress* or anxiety or mental or wellness)) ) OR ( ((beach or beaches or coastal or coastline* or seaside) N3 (health or wellbeing or stress or depress* or anxiety)) ) OR ( ("marine area*" N3 (health or wellbeing or stress or depress* or anxiety or mental or wellness)) ) OR ( ("marine environment*" N3 (health or wellbeing or stress or depress* or anxiety or mental or wellness)) ) OR ( ("aquatic area*" N3 (health or wellbeing or stress or depress* or anxiety or mental or wellness)) ) OR ( ("acquatic environment*" N3 (health or wellbeing or stress or depress* or anxiety or mental or wellness)) ) OR ( ((canal or fountain* or lake or lakes or lakeside or riparian or river or stream) N3 (health or wellbeing or stress or depress* or anxiety or mental or wellness)) ) OR ( (("water feature*" or waterfront* or waterscape* or waterway* or "inland water" or "inland surface water*" or "outdoor water environment*") N3 (health or wellbeing or stress or depress* or anxiety or mental or wellness)) )

S16 S12 OR S14 OR S15

S15 TI ( ((enhanc* or improv* or better) N1 sleep) ) OR AB ( ((enhanc* or improv* or better) N1 sleep) ) OR TI ( ((alleviat* or decreas* or lessen or manag* or reduc*) N2 pain) ) OR AB ( ((alleviat* or decreas* or lessen or manag* or reduc*) N2 pain) ) OR TI ( ((alleviat* or lessen or lower* or manag* or reduc*) N2 blood pressure) ) OR AB ( ((alleviat* or lessen or lower* or manag* or reduc*) N2 blood pressure) ) OR TI ( ((better or enhanc* or improv* or increas*) N1 "quality of life") ) OR AB ( ((better or enhanc* or improv* or increas*) N1 "quality of life") ) OR TI ( ((alleviat* or lessen or manag* or reduc*) N2 disabilit*) ) OR AB ( ((alleviat* or lessen or manag* or reduc*) N2 disabilit*) )

S14 S11 AND S13

S13 TI "Cardiovascular Disease" OR AB "cardiovascular disease" OR TI "heart disease" OR AB "heart disease" OR TI sleep OR AB sleep OR TI "quality of life" OR AB quality of life" OR TI pain OR AB pain OR TI disabilit* OR AB disabilit*

S12 S10 AND S11

S11 S5 OR S8 OR S9

S10 TI ( (mental* N2 (health or ill*)) ) OR AB ( (mental* N2 (health or ill*)) ) OR TI ( depression or depressive ) OR AB ( depression or depressive ) OR TI ( ((depressed or low) N2 mood) ) OR AB ( ((depressed or low) N2 mood) ) OR TI ( “mood disorder*” or “affective disorder*” or “anxiety or “psychological stress” or “stressful life vent*” or resilien* or wellbeing or well-being or “well being” ) OR AB ( “mood disorder*” or “affective disorder*” or “anxiety or “psychological stress” or “stressful life vent*” or resilien* or wellbeing or well-being or “well being” ) OR TI ( anxiety or "stressful life event*" or resilien* or wellbeing or well-being or "well being" ) OR AB ( anxiety or "stressful life event*" or resilien* or wellbeing or well-being or "well being" )

S9 TI ((natural or outdoor* or salutogenic) N2 environment*)) OR AB ( ((natural or outdoor* or salutogenic) N2 environment*) ) OR TI ( ((nature or natural) N2 space*) ) OR AB ( ((nature or natural) N2 space*) )

S8 S6 OR S7

S7 TI ( (bluespace* or "blue space*" or bluehealth or "blue water*" or "blue gym*" ) OR AB ( (bluespace* or "blue space*" or bluehealth or "blue water*" or "blue gym*" )

S6 ((DE "RIVERS") OR (DE "OCEAN")) OR (DE "COASTS")

S5 S1 OR S2 OR S3 OR S4

S4 TI ( (green N2 (area* or cover or environment* or gym* or neighbourhood* or neighborhood* or roadside* or space*)) ) OR AB ( (green N2 (area* or cover or environment* or gym* or neighbourhood* or neighborhood* or roadside* or space*)) ) OR TI ( ((city or cities or environment* or neighbourhood or neighborhood or urban) N2 greening) ) OR AB ( ((city or cities or environment* or neighbourhood or neighborhood or urban) N2 greening) ) OR TI ( ((ambient or city or cities or environment* or neighbourhood or neighborhood or residential or surrounding or urban) N2 greenness) ) OR AB ( ((ambient or city or cities or environment* or neighbourhood or neighborhood or residential or surrounding or urban) N2 greenness) ) OR TI ( (greenery or greenspace* OR garden* or allotment* or park* OR “sports field*” OR “wilderness area*” OR “public open space*” or “neighbourhood open space*” OR “neighborhood open space*” ) OR AB ( (greenery or greenspace* OR garden* or allotment* or park* OR “sports field*” OR “wilderness area*” OR “public open space*” or “neighbourhood open space*” OR “neighborhood open space*” )

S3 DE "NATIONAL parks & reserves"

S2 DE "FORESTS & forestry"

S1 DE "PARKS"

**Search 2**

S26

S24 NOT S25

Limiters - Publication Date: 20100101-20191231 (1,081)

S25 TI ( ( ( (clinical or surgery or surgical or cell or cells or laboratory or placebo or bladder or uterus or breast or gene or genes or genetic or bowel or liver or enzymes or viral or lymph or molecular) ) ) ) OR AB ( ( ( (clinical or surgery or surgical or cell or cells or laboratory or placebo or bladder or uterus or breast or gene or genes or genetic or bowel or liver or enzymes or viral or lymph or molecular) ) ) )

S24 S22 AND S23

S23 TI ( ( (Health* or (quality N3 life) or (well N3 being) or wellbeing or emotion*) ) ) OR AB ( ( (Health* or (quality N3 life) or (well N3 being) or wellbeing or emotion*) ) )

S22 S7 OR S10 OR S13 OR S17 OR S21

S21 S18 OR S19 OR S20

S20
TI ( ( ( (((communit* or local) N5 (garden* or park* or green* or greenspace or outdoor* or outside* or pavement* or sidewalk* or wood* or allotment* or lake* or canal* or river*)) and (work* or renewal or volunteer* or voluntary or practical or participat* or regenerat* or restor* or maintain* or enhance or preserve or creat*)) ) ) ) OR AB ( ( ( (((communit* or local) N5 (garden* or park* or green* or greenspace or outdoor* or outside* or pavement* or sidewalk* or wood* or allotment* or lake* or canal* or river*)) and (work* or renewal or volunteer* or voluntary or practical or participat* or regenerat* or restor* or maintain* or enhance or preserve or creat*)) ) )

S19 TI ( ( ( (communit* and (work* or renewal or volunteer* or voluntary or practical or regenerat* or restor* or maintain* or care or enhance* or preserve or creat* or activ* or action* or involve*) and ((natur* N3 environment*) or (environmental* and conservation*))) ) ) ) OR AB ( ( ( (communit* and (work* or renewal or volunteer* or voluntary or practical or regenerat* or restor* or maintain* or care or enhance* or preserve or creat* or activ* or action* or involve*) and ((natur* N3 environment*) or (environmental* and conservation*))) ) ) )

S18 TI ( ( ( ((communit* N5 (group* or team* or association* or organisation or organization or participa* or stakeholder* or steward* or trust* or ranger* or activit*)) and (garden* or allotment* or forest or (natural and environment) or conservation*)) ) ) ) OR AB ( ( ( ((communit* N5 (group* or team* or association* or organisation or organization or participa* or stakeholder* or steward* or trust* or ranger* or activit*)) and (garden* or allotment* or forest or (natural and environment) or conservation*)) ) ) )

S17 S14 OR S15 OR S16

S16 TI ( ( ( ((garden* or horticulture or allotment* or botanical or arboretum) N5 (maintain* or creat* or culivat* or enhance* or preserve or voluntary or volunteer or conservation* or participat*)) ) ) ) OR AB ( ( ( ((garden* or horticulture or allotment* or botanical or arboretum) N5 (maintain* or creat* or culivat* or enhance* or preserve or voluntary or volunteer or conservation* or participat*)) ) ) )

S15 TI ( ( ( ((garden* or horticulture or allotment* or botanical or arboretum) N5 (kitchen or school* or college* or university or campus or hospital* or prison* or penitentiary or institution or urban or green* or communit* or communal or group* or guerrilla or (bio N1 diver*) or eco or ((grow or pick) N3 your own))) ) ) ) OR AB ( ( ( ((garden* or horticulture or allotment* or botanical or arboretum) N5 (kitchen or school* or college* or university or campus or hospital* or prison* or penitentiary or institution or urban or green* or communit* or communal or group* or guerrilla or (bio N1 diver*) or eco or ((grow or pick) N3 your own))) ) ) )

S14 TI ( ( ( ((urban N3 (green* or park* or parkland or garden* or horticultur* or wood* or forest* or botanical or arboretum or allotment* or (open N1 space))) and (renewal or participate* or practical or regenerate* or restor* or maintain* or care or enhance* or preserve or great* or activ* or action* or involve* or engag*)) ) ) ) OR AB ( ( ( ((urban N3 (green* or park* or parkland or garden* or horticultur* or wood* or forest* or botanical or arboretum or allotment* or (open N1 space))) and (renewal or participate* or practical or regenerate* or restor* or maintain* or care or enhance* or preserve or great* or activ* or action* or involve* or engag*)) ) ) )

S13 S11 OR S12

S12 TI greenspace OR AB greenspace

S11 TI ( ( ( (Green* N3 (space* or gym or exercise or volunteer* or voluntary or conservation or infrastructure or care or streets or communal or Guerrilla)) ) ) ) OR AB ( ( ( (Green* N3 (space* or gym or exercise or volunteer* or voluntary or conservation or infrastructure or care or streets or communal or Guerrilla)) ) ) )

S10 S8 OR S9

S9 TI ( (((voluntary or volunteer*) N5 (group* or association or stakeholder* or steward* or ranger*)) and (environment* or nature or rural or countryside or outdoor* or outside or backcountry or hinterland or outback or wood* or park* or parkland or garden* or meadow* or horticultural or botanical or arboretum or allotment* or forest* or rainforest or moor* or dale* or marsh* or mountain* or beach* or wilderness or landscape* or tree* or copse* or river* or lake* or canal* or waterway or wetland* or (open N1 space*) or (protected N1 area*) or green* or planning* or footpath* or trail* or coast* or cliff* or dune* or (bio N1 diversity) or (eco N1 system) or (protected N1 area*))) ) OR AB ( (((voluntary or volunteer*) N5 (group* or association or stakeholder* or steward* or ranger*)) and (environment* or nature or rural or countryside or outdoor* or outside or backcountry or hinterland or outback or wood* or park* or parkland or garden* or meadow* or horticultural or botanical or arboretum or allotment* or forest* or rainforest or moor* or dale* or marsh* or mountain* or beach* or wilderness or landscape* or tree* or copse* or river* or lake* or canal* or waterway or wetland* or (open N1 space*) or (protected N1 area*) or green* or planning* or footpath* or trail* or coast* or cliff* or dune* or (bio N1 diversity) or (eco N1 system) or (protected N1 area*))) )

S8 TI ( ( ( (((Volunteer* or voluntary) N5 (environment* or nature or rural or countryside or outdoor* or outside or backcountry or hinterland or outback or wood* or park* or parkland or garden* or meadow* or horticultural or botanical or arboretum or allotment* or forest* or rainforest or moor* or dale* or marsh* or mountain* or beach* or wilderness or landscape* or tree* or copse* or river* or lake* or canal* or waterway or wetland* or (open N1 space*) or (protected N1 area*) or green* or planning* or footpath* or trail* or coast* or cliff* or dune* or (bio N1 diversity) or (eco N1 system) or (protected N1 area*))) and (renewal or participate* or practical or regenerate* or restor* or maintain* or care or enhance* or preserve or great* or activ* or action* or involve* or engag*)) ) ) ) OR AB ( ( ( (((Volunteer* or voluntary) N5 (environment* or nature or rural or countryside or outdoor* or outside or backcountry or hinterland or outback or wood* or park* or parkland or garden* or meadow* or horticultural or botanical or arboretum or allotment* or forest* or rainforest or moor* or dale* or marsh* or mountain* or beach* or wilderness or landscape* or tree* or copse* or river* or lake* or canal* or waterway or wetland* or (open N1 space*) or (protected N1 area*) or green* or planning* or footpath* or trail* or coast* or cliff* or dune* or (bio N1 diversity) or (eco N1 system) or (protected N1 area*))) and (renewal or participate* or practical or regenerate* or restor* or maintain* or care or enhance* or preserve or great* or activ* or action* or involve* or engag*)) ) ) )

S7 S1 OR S2 OR S3 OR S4 OR S5 OR S6

S6 TI ( ((activ* or practical or participat*) N3 (conservation*))) ) OR AB ( ((activ* or practical or participat*) N3 (conservation*))) )

S5 TI ( ( ( ((conservation* N5 (nature or rural or countryside or outdoor* or outside or backcountry or hinterland or outback or wood* or park* or parkland or garden* or meadow* or farm* or (farm N1 land) or horticultural or botanical or arboretum or allotment* or forest* or rainforest or moor* or dale* or marsh* or mountain* or beach* or wilderness or landscape* or tree* or copse* or river* or lake* or canal* or waterway or wetland* or (open N1 space*) or (protected N1 area*) or green* or planning* or footpath* or trail* or coast* or cliff* or dune* or (bio N1 diversity) or (eco N1 system) or (protected N1 area*))) and (renewal or volunteer* or voluntary or participate* or practical or regenerate* or restor* or maintain* or care or enhance* or preserve or great* or activ* or action* or involve*)) ) ) ) OR AB ( ( ( ((conservation* N5 (nature or rural or countryside or outdoor* or outside or backcountry or hinterland or outback or wood* or park* or parkland or garden* or meadow* or farm* or (farm N1 land) or horticultural or botanical or arboretum or allotment* or forest* or rainforest or moor* or dale* or marsh* or mountain* or beach* or wilderness or landscape* or tree* or copse* or river* or lake* or canal* or waterway or wetland* or (open N1 space*) or (protected N1 area*) or green* or planning* or footpath* or trail* or coast* or cliff* or dune* or (bio N1 diversity) or (eco N1 system) or (protected N1 area*))) and (renewal or volunteer* or voluntary or participate* or practical or regenerate* or restor* or maintain* or care or enhance* or preserve or great* or activ* or action* or involve*)) ) ) )

S4 TI ( ( ( ((conservation* N3 (group* or volunteer* or voluntary or association* or organisation* or organization* or participa* or stakeholder* or steward* or trust or ranger* or activit*)) and (renewal or volunteer* or voluntary or participate* or practical or regenerate* or restor* or maintain* or care or enhance* or preserve or great* or activ* or action* or involve*)) ) ) ) OR AB ( ( ( ((conservation* N3 (group* or volunteer* or voluntary or association* or organisation* or organization* or participa* or stakeholder* or steward* or trust or ranger* or activit*)) and (renewal or volunteer* or voluntary or participate* or practical or regenerate* or restor* or maintain* or care or enhance* or preserve or great* or activ* or action* or involve*)) ) ) )

S3 TI ( ( ( ((environmental* N3 (conservation* or volunteer* or steward*)) and (Regenerat* or restore or restoration or redevelop or maintain or enhance or preserve or preserving or create or creation or establish or establishing or founding or build* or cultivat* or cultivation or participate or participation)) ) ) ) OR AB ( ( ( ((environmental* N3 (conservation* or volunteer* or steward*)) and (Regenerat* or restore or restoration or redevelop or maintain or enhance or preserve or preserving or create or creation or establish or establishing or founding or build* or cultivat* or cultivation or participate or participation)) ) ) )

S2 TI (Conservation N3 interventions) OR AB (Conservation N3 interventions)

S1 TI ( ( ( (conservation* and natural and environment* and (renewal or volunteer* or voluntary or participate* or practical or regenerate* or restor* or maintain* or care or enhance* or preserve or great* or activ* or action* or involve*)) ) ) ) OR AB ( ( ( (conservation* and natural and environment* and (renewal or volunteer* or voluntary or participate* or practical or regenerate* or restor* or maintain* or care or enhance* or preserve or great* or activ* or action* or involve*)) ) ) )

**MEDLINE Via OVID; Search date = 7^th^ October 2019**

**Database: Ovid MEDLINE(R) ALL <1946 to October 04, 2019>**

1 *Environment/ or *Environment Design/ (28938)

2 *Residence Characteristics/ (12943)

3 *Parks, Recreational/ (431)

4 (green adj2 (area$ or cover or environment$ or gym$ or neighbourhood$ or neighborhood$ or roadside$ or space$)).ti,ab,kw. (2076)

5 ((city or cities or environment$ or neighbourhood or neighborhood or urban) adj2 greening).ti,ab,kw. (88)

6 ((ambient or city or cities or environment$ or neighbourhood or neighborhood or residential or surrounding or urban) adj2 greenness).ti,ab,kw. (139)

7 (greenery or greenspace$).ti,ab,kw. (338)

8 (garden$ or park$1 or parkland$).ti,ab,kw. (32081)

9 sports field$.ti,ab,kw. (87)

10 wilderness area$.ti,ab,kw. (169)

11 public open space$.ti,ab,kw. (88)

12 neighbourhood open space$.ti,ab,kw. (1)

13 neighborhood open space$.ti,ab,kw. (3)

14 1 or 2 or 3 or 4 or 5 or 6 or 7 or 8 or 9 or 10 or 11 or 12 or 13 (73994)

15 *Water/ (59999)

16 *Rivers/ (11743)

17 *"Oceans and Seas"/ (1034)

18 (bluespace$ or blue space$).ti,ab,kw. (90)

19 bluehealth.ti,ab,kw. (1)

20 blue water$.ti,ab,kw. (161)

21 blue gym$.ti,ab,kw. (3)

22 15 or 16 or 17 or 18 or 19 or 20 or 21 (72727)

23 *Nature/ (515)

24 (health promot$ adj2 environment$).ti,ab,kw. (295)

25 ((natural or outdoor$ or salutogenic) adj2 environment$).ti,ab,kw. (13532)

26 ((nature or natural) adj2 space$).ti,ab,kw. (352)

27 23 or 24 or 25 or 26 (14613)

28 14 or 22 or 27 (159355)

29 Mental Health/ (35112)

30 (mental$ adj2 (health or ill$)).ti,ab,kw. (155083)

31 exp Depressive Disorder/ (105338)

32 Depression/ (111943)

33 (depression or depressive).ti,ab,kw. (359958)

34 ((depressed or low) adj2 mood).ti,ab,kw. (5443)

35 Mood Disorders/ (13910)

36 affective disorder$.ti,ab,kw. (16285)

37 exp Anxiety Disorders/ (77261)

38 (anxiety or anxious).ti,ab,kw. (184605)

39 exp Stress, Psychological/ (125083)

40 (stressful life event$ or resilien$).ti,ab,kw. (32814)

41 (wellbeing or well-being or well being).ti,ab,kw. (82984)

42 29 or 30 or 31 or 32 or 33 or 34 or 35 or 36 or 37 or 38 or 39 or 40 or 41 (841232)

43 28 and 42 (5843)

44 exp animals/ not humans.sh. (4625045)

45 43 not 44 (5137)

46 Cardiovascular Disease/ (140902)

47 Sleep/ (50281)

48 "Quality of life"/ (182138)

49 "Activities of Daily Living"/ (62231)

50 Hypertension/ (228483)

51 Pain/ (130166)

52 ((enhanc$ or improv$ or better) adj sleep).ti,ab. (4090)

53 ((alleviat$ or decreas$ or lessen or manag$ or reduc$) adj2 pain).ti,ab. (65293)

54 ((alleviat$ or lessen or lower$ or manag$ or reduc$) adj2 blood pressure).ti,ab. (30631)

55 ((better or enhanc$ or improv$ or increas$) adj quality of life).ti,ab. (21415)

56 ((alleviat$ or lessen or manag$ or reduc$) adj2 disabilit$).ti,ab. (3327)

57 46 or 47 or 48 or 49 or 50 or 51 or 52 or 53 or 54 or 55 or 56 (829946)

58 28 and 57 (3019)

59 exp animals/ not humans.sh. (4625045)

60 58 not 59 (2875)

61 ((greenspace$ or green space$ or greenery or greenness) adj3 (health or wellbeing or stress or depress$ or anxiety or mental or wellness)).ti,ab,kw. (210)

62 ((allotment$ or garden or gardens or park or parks or parkland$) adj3 (health or wellbeing or stress or depress$ or anxiety or mental or wellness)).ti,ab,kw. (284)

63 ((bushland$ or countryside or forest$ or woodland$) adj3 (health or wellbeing or stress or depress$ or anxiety or mental or wellness)).ti,ab,kw. (558)

64 ((bluespace$ or blue space$) adj3 (health or wellbeing or stress or depress$ or anxiety or mental or wellness)).ti,ab,kw. (30)

65 ((beach or beaches or coastal or coastline$ or seaside) adj3 (health or wellbeing or stress or depress$ or anxiety)).ti,ab,kw. (379)

66 (marine area$ adj3 (health or wellbeing or stress or depress$ or anxiety or mental or wellness)).ti,ab,kw. (0)

67 (marine environment$ adj3 (health or wellbeing or stress or depress$ or anxiety or mental or wellness)).ti,ab,kw. (71)

68 (aquatic area$ adj3 (health or wellbeing or stress or depress$ or anxiety or mental or wellness)).ti,ab,kw. (0)

69 (acquatic environment$ adj3 (health or wellbeing or stress or depress$ or anxiety or mental or wellness)).ti,ab,kw. (0)

70 ((canal or fountain$ or lake or lakes or lakeside or riparian or river or stream) adj3 (health or wellbeing or stress or depress$ or anxiety or mental or wellness)).ti,ab,kw. (727)

71 ((water feature$ or waterfront$ or waterscape$ or waterway$ or inland water or inland surface water$ or outdoor water environment$) adj3 (health or wellbeing or stress or depress$ or anxiety or mental or wellness)).ti,ab,kw. (13)

72 61 or 62 or 63 or 64 or 65 or 66 or 67 or 68 or 69 or 70 or 71 (2239)

73 limit 72 to humans (1086)

74 45 or 60 or 73 (8358)

75 Physical Fitness/ (26570)

76 exp Recreation/ (199134)

77 Leisure Activities/ (8336)

78 exp Exercise/ (183450)

79 exp Exercise Therapy/ (47481)

80 (physical adj3 (fit$ or train$ or activ$ or endur$)).ti,ab. (120932)

81 (exercis$ adj3 (fit$ or train$ or activ$ or endur$)).ti,ab. (36875)

82 ((promot$ or uptak$ or encourag$ or increas$ or start$ or adher$) adj3 (exercis$ or sport$ or fitness)).ti,ab. (33155)

83 Running/ or Jogging/ or Walking/ (48975)

84 (sport$ or walk$ or running or jogging or bicycling or biking or trekking or rambling or forest bathing).ti,ab. (230284)

85 exp water sports/ or swimming/ or diving/ (23939)

86 (boating or canoeing or diving or kayaking or rowing or surfboarding or swimming or water skiing or water sport$).ti,ab,kw. (34989)

87 ((craft or creative or leisure) adj2 (activit$ or task$)).ti,ab. (5560)

88 Gardening/ or gardening.ti,ab,kw. (1904)

89 75 or 76 or 77 or 78 or 79 or 80 or 81 or 82 or 83 or 84 or 85 or 86 or 87 or 88 (576569)

90 74 and 89 (1530)

91 limit 90 to (english language and yr="2010 -Current") (1095)

92 (conservation$ and natural and environment$ and (renewal or volunteer$ or voluntary or participat$ or practical or regenerat$ or restor$ or maintain$ or care or enhance$ or preserve or creat$ or activ$ or action$ or involve$)).ti,ab. (1064)

93 ((Conservation adj3 interventions) or ecotherap$).ti,ab. (142)

94 ((environmental$ adj3 (conservation$ or volunteer$ or steward$)) and (Regenerat$ or restore or restoration or redevelop or maintain or enhance or preserve or preserving or create or creation or establish or establishing or founding or build$ or cultivat$ or cultivation or participate or participation)).ti,ab. (212)

95 (conservation$ adj3 (group$ or volunteer$ or voluntary or association$ or organisation$ or organization$ or participa$ or stakeholder$ or steward$ or trust or ranger$ or activit$)).ti,ab. (1425)

96 (conservation$ adj3 (nature or rural or countryside or outdoor$ or outside or backcountry or hinterland or outback or wood$ or park$1 or parkland or garden$ or meadow$ or farm$ or (farm adj1 land) or horticultural or floricultural or botanical or arboretum or allotment$ or forest$ or rainforest or moor$ or dale$1 or marsh$ or mountain$ or beach$ or wilderness or landscape$ or tree$ or copse$ or river$ or lake$ or canal$ or waterway or wetland$ or (open adj1 space$) or (protected adj1 area$) or green$ or planning$ or footpath$ or trail$ or coast$ or cliff$ or dune$ or (bio adj1 diversity) or (eco adj1 system) or (protected adj1 area$))).ti,ab. (3762)

97 (geoconservation or (geo adj3 conservation)).ti,ab. (7)

98 ((activ$ or practical or participat$) adj3 conservation$).ti,ab. (924)

99 exp "Conservation of Natural Resources"/ or *Environment/ or *Environment Design/ (123965)

100 (volunteer$ or voluntary).ti,ab. or *Voluntary Workers/ or *Consumer Participation/ or *Health Status/ (289942)

101 99 and 100 (1158)

102 92 or 93 or 94 or 95 or 96 or 97 or 98 or 101 (7552)

103 ((Volunteer$ or voluntary) adj3 (environment$ or nature or rural or countryside or outdoor$ or outside or backcountry or hinterland or outback or wood$ or park$1 or parkland or garden$ or meadow$ or horticultural or floricultural or botanical or arboretum or allotment$ or forest$ or rainforest or moor$ or dale$1 or marsh$ or mountain$ or beach$ or wilderness or landscape$ or tree$ or copse$ or river$ or lake$ or canal$ or waterway or wetland$ or (open adj1 space$) or (protected adj1 area$) or green$ or planning$ or footpath$ or trail$ or coast$ or cliff$ or dune$ or (bio adj1 diversity) or (eco adj1 system) or (protected adj1 area$))).ti,ab. (993)

104 (((voluntary or volunteer$) adj3 (group$ or association or stakeholder$ or steward$ or ranger$)) and (environment$ or nature or rural or countryside or outdoor$ or outside or backcountry or hinterland or outback or wood$ or park$1 or parkland or garden$ or meadow$ or horticultural or floricultural or botanical or arboretum or allotment$ or forest$ or rainforest or moor$ or dale$1 or marsh$ or mountain$ or beach$ or wilderness or landscape$ or tree$ or copse$ or river$ or lake$ or canal$ or waterway or wetland$ or (open adj1 space$) or (protected adj1 area$) or green$ or planning$ or footpath$ or trail$ or coast$ or cliff$ or dune$ or (bio adj1 diversity) or (eco adj1 system) or (protected adj1 area$))).ti,ab. (673)

105 *Voluntary Workers/ (5574)

106 (environment$ or nature or rural or countryside or outdoor$ or outside or backcountry or hinterland or outback or wood$ or park$1 or parkland or garden$ or meadow$ or horticultural or floricultural or botanical or arboretum or allotment$ or forest$ or rainforest or moor$ or dale$1 or marsh$ or mountain$ or beach$ or wilderness or landscape$ or tree$ or copse$ or river$ or lake$ or canal$ or waterway or wetland$ or (open adj1 space$) or (protected adj1 area$) or green$ or planning$ or footpath$ or trail$ or coast$ or cliff$ or dune$ or (bio adj1 diversity) or (eco adj1 system) or (protected adj1 area$)).ti,ab. (2352164)

107 105 and 106 (578)

108 103 or 104 or 107 (2090)

109 (Green$ adj3 (space$ or gym or exercise or volunteer$ or voluntary or conservation or infrastructure or care or streets or communal or Guerrilla)).ti,ab. (1697)

110 greenspace.ti,ab. (160)

111 109 or 110 (1835)

112 (urban adj3 (green$ or park$1 or parkland or garden$ or horticultur$ or wood$ or forest$ or botanical or arboretum or allotment$ or (open adj1 space))).ti,ab. (2140)

113 ((work$ or renewal or volunteer$ or voluntary or practical or regenerat$ or restor$ or maintain$ or care or enhance or preserve or creat$) and (urban or city or metropolis or town$) and (garden$ or park$1 or parkland or allotment$)).ti,ab. (860)

114 *Cities/ and ((work$ or renewal or volunteer$ or voluntary or practical or regenerat$ or restor$ or maintain$ or care or enhance or preserve or creat$) and (garden$ or park$1 or parkland or allotment$)).ti,ab. (34)

115 *Urban Health/ and (*Conservation of Natural Resources/ or *Voluntary Workers/) (25)

116 112 or 113 or 114 or 115 (2845)

117 ((garden$ or horticulture or allotment$ or botanical or arboretum) adj3 (kitchen or school$ or college$ or university or campus or hospital$ or prison$ or penitentiary or institution or urban or green$ or communit$ or communal or group$ or guerrilla or (bio adj1 diver$) or eco or ((grow or pick) adj3 your own))).ti,ab. (1152)

118 ((garden$ or horticulture or allotment$ or botanical or arboretum) adj3 (maintain$ or creat$ or culivat$ or enhance$ or preserve or voluntary or volunteer or conservation$ or participat$)).ti,ab. (204)

119 Gardening/ and (*Conservation of Natural Resources/ or *Voluntary Workers/) (31)

120 *Gardening/ and (kitchen or school$ or college$ or university or campus or hospital$ or prison$ or penitentiary or institution or urban or green$ or communit$ or communal or group$ or guerrilla or (bio adj1 diver$) or eco or maintain$ or creat$ or culivat$ or voluntary or volunteer or conservation$ or participat$).ti,ab. (324)

121 117 or 118 or 119 or 120 (1469)

122 ((communit$ adj3 (group$ or team$ or association$ or organisation or organization or participa$ or stakeholder$ or steward$ or trust$ or ranger$ or activit$)) and (garden$ or allotment$ or forest or (natural and environment) or conservation$)).ti,ab. (650)

123 (communit$ and (work$ or renewal or volunteer$ or voluntary or practical or regenerat$ or restor$ or maintain$ or care or enhance$ or preserve or creat$ or activ$ or action$ or involve$) and ((natur$ adj3 environment$) or (environmental$ and conservation$))).ti,ab. (1391)

124 (((communit$ or local) adj3 (garden$ or park$1 or parkland$ or green$ or greenspace or outdoor$ or outside$ or pavement$ or sidewalk$ or wood$ or allotment$ or lake$ or canal$ or river$)) and (work$ or renewal or volunteer$ or voluntary or practical or participat$ or regenerat$ or restor$ or maintain$ or enhance or preserve or creat$)).ti,ab. (1209)

125 122 or 123 or 124 (3067)

126 102 or 108 or 111 or 116 or 121 or 125 (17087)

127 126 and (42 or 57) (1676)

128 limit 127 to (english language and yr="2010 -Current") (1284)

129 91 or 128 (2073)

**PsycINFO, Via OVID; Search date=7^th^ October 2019**

Database: PsycINFO <2002 to September Week 5 2019>

1 "Nature (Environment)"/ or Built Environment/ (2063)

2 Rural Environment/ or Urban Environment/ (24881)

3 Neighborhoods/ (6610)

4 Recreation Areas/ (611)

5 (green adj2 (area$ or cover or environment$ or gym$ or neighbourhood$ or neighborhood$ or roadside$ or space$)).ti,ab,id. (495)

6 ((city or cities or environment$ or neighbourhood or neighborhood or urban) adj2 greening).ti,ab,id. (15)

7 ((ambient or city or cities or environment$ or neighbourhood or neighborhood or residential or surrounding or urban) adj2 greenness).ti,ab,id. (13)

8 (greenery or greenspace$).ti,ab,id. (100)

9 (garden$ or park$1 or parkland$).ti,ab,id. (6617)

10 sports field$.ti,ab,id. (41)

11 wilderness area$.ti,ab,id. (27)

12 public open space$.ti,ab,id. (40)

13 neighbourhood open space$.ti,ab,id. (0)

14 neighborhood open space$.ti,ab,id. (3)

15 1 or 2 or 3 or 4 or 5 or 6 or 7 or 8 or 9 or 10 or 11 or 12 or 13 or 14 (38683)

16 (bluespace$ or blue space$).ti,ab,id. (26)

17 bluehealth.ti,ab,id. (0)

18 blue water$.ti,ab,id. (3)

19 blue gym$.ti,ab,id. (1)

20 16 or 17 or 18 or 19 (30)

21 (health promot$ adj2 environment$).ti,ab,id. (89)

22 ((natural or outdoor$ or salutogenic) adj2 environment$).ti,ab,id. (3385)

23 ((nature or natural) adj2 space$).ti,ab,id. (122)

24 21 or 22 or 23 (3578)

25 15 or 20 or 24 (41765)

26 *Mental Health/ (35874)

27 (mental$ adj2 (health or ill$)).ti,ab,id. (150896)

28 *Affective Disorders/ (5782)

29 *Major Depression/ (68293)

30 *"Depression (Emotion)"/ (5334)

31 (depression or depressive).ti,ab,id. (190500)

32 ((depressed or low) adj2 mood).ti,ab,id. (3871)

33 *Mood Disorders/ (5782)

34 affective disorder$.ti,ab,id. (8056)

35 *Anxiety Disorders/ (9561)

36 (anxiety or anxious).ti,ab,id. (127445)

37 *Stress/ (26659)

38 (stressful life event$ or resilien$).ti,ab,id. (29882)

39 (wellbeing or well-being or well being).ti,ab,id. (73916)

40 26 or 27 or 28 or 29 or 30 or 31 or 32 or 33 or 34 or 35 or 36 or 37 or 38 or 39 (468731)

41 25 and 40 (7315)

42 (animal not human).po. (195784)

43 41 not 42 (7264)

44 Cardiovascular Disorders/ (6583)

45 Sleep/ (15077)

46 "Quality of life"/ (33426)

47 "Activities of Daily Living"/ (4185)

48 Daily Activities/ (2838)

49 Hypertension/ (4305)

50 Pain/ (18084)

51 ((enhanc$ or improv$ or better) adj sleep).ti,ab. (1684)

52 ((alleviat$ or decreas$ or lessen or manag$ or reduc$) adj2 pain).ti,ab. (10420)

53 ((alleviat$ or lessen or lower$ or manag$ or reduc$) adj2 blood pressure).ti,ab. (939)

54 ((better or enhanc$ or improv$ or increas$) adj quality of life).ti,ab. (4250)

55 ((alleviat$ or lessen or manag$ or reduc$) adj2 disabilit$).ti,ab. (1035)

56 44 or 45 or 46 or 47 or 48 or 49 or 50 or 51 or 52 or 53 or 54 or 55 (92427)

57 25 and 56 (1128)

58 (animal not human).po. (195784)

59 57 not 58 (1112)

60 ((greenspace$ or green space$ or greenery or greenness) adj3 (health or wellbeing or stress or depress$ or anxiety or mental or wellness)).ti,ab,id. (48)

61 ((allotment$ or garden or gardens or park or parks or parkland$) adj3 (health or wellbeing or stress or depress$ or anxiety or mental or wellness)).ti,ab,id. (83)

62 ((bushland$ or countryside or forest$ or woodland$) adj3 (health or wellbeing or stress or depress$ or anxiety or mental or wellness)).ti,ab,id. (42)

63 ((bluespace$ or blue space$) adj3 (health or wellbeing or stress or depress$ or anxiety or mental or wellness)).ti,ab,id. (5)

64 ((beach or beaches or coastal or coastline$ or seaside) adj3 (health or wellbeing or stress or depress$ or anxiety)).ti,ab,id. (46)

65 (marine area$ adj3 (health or wellbeing or stress or depress$ or anxiety or mental or wellness)).ti,ab,id. (0)

66 (marine environment$ adj3 (health or wellbeing or stress or depress$ or anxiety or mental or wellness)).ti,ab,id. (4)

67 (aquatic area$ adj3 (health or wellbeing or stress or depress$ or anxiety or mental or wellness)).ti,ab,id. (0)

68 (acquatic environment$ adj3 (health or wellbeing or stress or depress$ or anxiety or mental or wellness)).ti,ab,id. (0)

69 ((canal or fountain$ or lake or lakes or lakeside or riparian or river or stream) adj3 (health or wellbeing or stress or depress$ or anxiety or mental or wellness)).ti,ab,id. (67)

70 ((water feature$ or waterfront$ or waterscape$ or waterway$ or inland water or inland surface water$ or outdoor water environment$) adj3 (health or wellbeing or stress or depress$ or anxiety or mental or wellness)).ti,ab,id. (1)

71 60 or 61 or 62 or 63 or 64 or 65 or 66 or 67 or 68 or 69 or 70 (292)

72 (animal not human).po. (195784)

73 71 not 72 (284)

74 43 or 59 or 73 (8223)

75 *Health Behavior/ (16930)

76 Physical Fitness/ (2732)

77 Physical Activity/ (17595)

78 exp Recreation/ (43359)

79 Leisure Time/ (3862)

80 exp Exercise/ (19592)

81 Aerobic Exercise/ (1232)

82 (physical adj3 (fit$ or train$ or activ$ or endur$)).ti,ab. (32133)

83 (exercis$ adj3 (fit$ or train$ or activ$ or endur$)).ti,ab. (4810)

84 ((promot$ or uptak$ or encourag$ or increas$ or start$ or adher$) adj3 (exercis$ or sport$ or fitness)).ti,ab. (5066)

85 Running/ or Walking/ (5418)

86 (sport$ or walk$ or running or jogging or bicycling or biking or trekking or rambling or forest bathing).ti,ab. (53787)

87 exp water sports/ or swimming/ or diving/ (990)

88 (boating or canoeing or diving or kayaking or rowing or surfboarding or swimming or water skiing or water sport$).ti,ab. (4216)

89 ((craft or creative or leisure) adj2 (activit$ or task$)).ti,ab. (4044)

90 Horticulture therapy/ or gardening.ti,ab,id. (517)

91 75 or 76 or 77 or 78 or 79 or 80 or 81 or 82 or 83 or 84 or 85 or 86 or 87 or 88 or 89 or 90 (135190)

92 74 and 91 (984)

93 limit 92 to (english language and yr="2010 -Current") (732)

94 (conservation$ and natural and environment$ and (renewal or volunteer$ or voluntary or participat$ or practical or regenerat$ or restor$ or maintain$ or care or enhance$ or preserve or creat$ or activ$ or action$ or involve$)).ti,ab. (173)

95 ((Conservation adj3 interventions) or ecotherap$).ti,ab. (56)

96 ((environmental$ adj3 (conservation$ or volunteer$ or steward$)) and (Regenerat$ or restore or restoration or redevelop or maintain or enhance or preserve or preserving or create or creation or establish or establishing or founding or build$ or cultivat$ or cultivation or participate or participation)).ti,ab. (99)

97 (conservation$ adj3 (group$ or volunteer$ or voluntary or association$ or organisation$ or organization$ or participa$ or stakeholder$ or steward$ or trust or ranger$ or activit$)).ti,ab. (197)

98 (conservation$ adj3 (nature or rural or countryside or outdoor$ or outside or backcountry or hinterland or outback or wood$ or park$1 or parkland or garden$ or meadow$ or farm$ or (farm adj1 land) or horticultural or floricultural or botanical or arboretum or allotment$ or forest$ or rainforest or moor$ or dale$1 or marsh$ or mountain$ or beach$ or wilderness or landscape$ or tree$ or copse$ or river$ or lake$ or canal$ or waterway or wetland$ or (open adj1 space$) or (protected adj1 area$) or green$ or planning$ or footpath$ or trail$ or coast$ or cliff$ or dune$ or (bio adj1 diversity) or (eco adj1 system) or (protected adj1 area$))).ti,ab. (237)

99 (geoconservation or (geo adj3 conservation)).ti,ab. (0)

100 ((activ$ or practical or participat$) adj3 conservation$).ti,ab. (97)

101 *environment/ (5337)

102 (volunteer$ or voluntary).ti,ab. or *Volunteers/ or *Consumer Behavior/ or *Health Status/ (60994)

103 101 and 102 (137)

104 94 or 95 or 96 or 97 or 98 or 99 or 100 or 103 (812)

105 ((Volunteer$ or voluntary) adj3 (environment$ or nature or rural or countryside or outdoor$ or outside or backcountry or hinterland or outback or wood$ or park$1 or parkland or garden$ or meadow$ or horticultural or botanical or arboretum or allotment$ or forest$ or rainforest or moor$ or dale$1 or marsh$ or mountain$ or beach$ or wilderness or landscape$ or tree$ or copse$ or river$ or lake$ or canal$ or waterway or wetland$ or (open adj1 space$) or (protected adj1 area$) or green$ or planning$ or footpath$ or trail$ or coast$ or cliff$ or dune$ or (bio adj1 diversity) or (eco adj1 system) or (protected adj1 area$))).ti,ab. (507)

106 (((voluntary or volunteer$) adj3 (group$ or association or stakeholder$ or steward$ or ranger$)) and (environment$ or nature or rural or countryside or outdoor$ or outside or backcountry or hinterland or outback or wood$ or park$1 or parkland or garden$ or meadow$ or horticultural or botanical or arboretum or allotment$ or forest$ or rainforest or moor$ or dale$1 or marsh$ or mountain$ or beach$ or wilderness or landscape$ or tree$ or copse$ or river$ or lake$ or canal$ or waterway or wetland$ or (open adj1 space$) or (protected adj1 area$) or green$ or planning$ or footpath$ or trail$ or coast$ or cliff$ or dune$ or (bio adj1 diversity) or (eco adj1 system) or (protected adj1 area$))).ti,ab. (246)

107 *Volunteers/ (2098)

108 (environment$ or nature or rural or countryside or outdoor$ or outside or backcountry or hinterland or outback or wood$ or park$1 or parkland or garden$ or meadow$ or horticultural or botanical or arboretum or allotment$ or forest$ or rainforest or moor$ or dale$1 or marsh$ or mountain$ or beach$ or wilderness or landscape$ or tree$ or copse$ or river$ or lake$ or canal$ or waterway or wetland$ or (open adj1 space$) or (protected adj1 area$) or green$ or planning$ or footpath$ or trail$ or coast$ or cliff$ or dune$ or (bio adj1 diversity) or (eco adj1 system) or (protected adj1 area$)).ti,ab. (462946)

109 107 and 108 (388)

110 105 or 106 or 109 (1020)

111 (Green$ adj3 (space$ or gym or exercise or volunteer$ or voluntary or conservation or infrastructure or care or streets or communal or Guerrilla)).ti,ab. (414)

112 greenspace.ti,ab. (42)

113 111 or 112 (448)

114 (urban adj3 (green$ or park$1 or parkland or garden$ or horticultur$ or wood$ or forest$ or botanical or arboretum or allotment$ or (open adj1 space))).ti,ab. (318)

115 ((work$ or renewal or volunteer$ or voluntary or practical or regenerat$ or restor$ or maintain$ or care or enhance or preserve or creat$) and (urban or city or metropolis or town$) and (garden$ or park$1 or parkland or allotment$)).ti,ab. (433)

116 (city or cities).ti. (6261)

117 (work$ or renewal or volunteer$ or voluntary or practical or regenerat$ or restor$ or maintain$ or care or enhance or preserve or creat$).ti,ab. (974129)

118 (garden$ or park$1 or parkland or allotment$).ti,ab. (6668)

119 116 and 117 and 118 (52)

120 114 or 115 or 119 (674)

121 ((garden$ or horticulture or allotment$ or botanical or arboretum) adj3 (kitchen or school$ or college$ or university or campus or hospital$ or prison$ or penitentiary or institution or urban or green$ or communit$ or communal or group$ or guerrilla or (bio adj1 diver$) or eco or ((grow or pick) adj3 your own))).ti,ab. (361)

122 ((garden$ or horticulture or allotment$ or botanical or arboretum) adj3 (maintain$ or creat$ or culivat$ or enhance$ or preserve or voluntary or volunteer or conservation$ or participat$)).ti,ab. (101)

123 Horticulture Therapy/ and Volunteers/ (0)

124 gardening.ti. (112)

125 (kitchen or school$ or college$ or university or campus or hospital$ or prison$ or penitentiary or institution or urban or green$ or communit$ or communal or group$ or guerrilla or (bio adj1 diver$) or eco or maintain$ or creat$ or culivat$ or voluntary or volunteer or conservation$ or participat$).ti,ab. (1252748)

126 124 and 125 (86)

127 121 or 122 or 123 or 126 (443)

128 ((communit$ adj3 (group$ or team$ or association$ or organisation or organization or participa$ or stakeholder$ or steward$ or trust$ or ranger$ or activit$)) and (garden$ or allotment$ or forest or (natural and environment) or conservation$)).ti,ab. (215)

129 (communit$ and (work$ or renewal or volunteer$ or voluntary or practical or regenerat$ or restor$ or maintain$ or care or enhance$ or preserve or creat$ or activ$ or action$ or involve$) and ((natur$ adj3 environment$) or (environmental$ and conservation$))).ti,ab. (454)

130 (((communit$ or local) adj3 (garden$ or park$1 or parkland$ or green$ or greenspace or outdoor$ or outside$ or pavement$ or sidewalk$ or wood$ or allotment$ or lake$ or canal$ or river$)) and (work$ or renewal or volunteer$ or voluntary or practical or participat$ or regenerat$ or restor$ or maintain$ or enhance or preserve or creat$)).ti,ab. (729)

131 128 or 129 or 130 (1302)

132 104 or 110 or 113 or 120 or 127 or 131 (4082)

133 (clinical or surgery or surgical or cell or cells or laboratory or placebo or bladder or uterus or breast or gene or genes or genetic or bowel or liver or enzymes or viral or lymph or molecular).mp. (637437)

134 132 not 133 (3745)

135 limit 134 to (english language and yr="2010 -Current") (2557)

136 (animal not human).po. (195784)

137 135 not 136 (2483)

138 93 or 137 (3048)

**Science Citation Index via Web of Science; Search date=7^th^ October 2019**

**Search 1**

# 41 2,534

(#39 not #40) AND LANGUAGE: (English)

Indexes=SCI-EXPANDED, SSCI Timespan=2010-2019

# 40 601,649

(TI=(rat or rats or mouse or mice or bird or birds or cow or cattle or bovine or sheep or goat* or ovine or horse or equine or pig or pigs or porcine or fish or fishes)) AND LANGUAGE: (English)

Indexes=SCI-EXPANDED, SSCI Timespan=2010-2019

# 39 2,587

#38 AND #32

Indexes=SCI-EXPANDED, SSCI Timespan=2010-2019

# 38 627,705

#37 OR #36 OR #35 OR #34 OR #33

Indexes=SCI-EXPANDED, SSCI Timespan=2010-2019

# 37 6,809

(TS=(((craft or creative or leisure) NEAR/2 (activit* or task*)))) AND LANGUAGE: (English)

Indexes=SCI-EXPANDED, SSCI Timespan=2010-2019

# 36 402,960

(TS=(((Running or Jogging or Walking OR sport* or bicycling or biking or trekking or rambling or "forest bathing" or gardening or "water sport*" OR swimming or diving OR boating or canoeing or Kayaking or rowing or surfboarding or "water skiing")))) AND LANGUAGE: (English)

Indexes=SCI-EXPANDED, SSCI Timespan=2010-2019

# 35 19,617

(TS=(((promot* or uptak* or encourag* or increas* or start* or adher*) NEAR/3 (exercis* or sport* or fitness)))) AND LANGUAGE: (English)

Indexes=SCI-EXPANDED, SSCI Timespan=2010-2019

# 34 134,149

(TS=(((physical Or exercise) NEAR/3 (fit* or train* or activ* or endur*)))) AND LANGUAGE: (English)

Indexes=SCI-EXPANDED, SSCI Timespan=2010-2019

# 33 265,977

(TS=("Physical Fitness" OR "physical activity" OR Recreation or "leisure activit*" or Exercise or "exercise therap*" )) AND LANGUAGE: (English)

Indexes=SCI-EXPANDED, SSCI Timespan=2010-2019

# 32 10,321

#31 OR #21

Indexes=SCI-EXPANDED, SSCI Timespan=2010-2019

# 31 5,265

(#30 OR #29 OR #28 OR #27 OR #26 OR #25 OR #24 OR #23 OR #22) AND LANGUAGE: (English)

Indexes=SCI-EXPANDED, SSCI Timespan=2010-2019

# 30 37

(TS=((("water feature*" or waterfront* or waterscape* or waterway* or "inland water" or "inland surface water*" or "outdoor water environment*") NEAR/3 (health or wellbeing or stress or depress* or anxiety or mental or wellness)))) AND LANGUAGE: (English)

Indexes=SCI-EXPANDED, SSCI Timespan=2010-2019

# 29 2,178

(TS=(((canal or fountain* or lake or lakes or lakeside or riparian or river or stream) NEAR/3 (health or wellbeing or stress or depress* or anxiety or mental or wellness)))) AND LANGUAGE: (English)

Indexes=SCI-EXPANDED, SSCI Timespan=2010-2019

# 28 101

(TS=(("marine environment*" NEAR/3 (health or wellbeing or stress or depress* or anxiety or mental or wellness)))) AND LANGUAGE: (English)

Indexes=SCI-EXPANDED, SSCI Timespan=2010-2019

# 27 5

(TS=(("marine area*" NEAR/3 (health or wellbeing or stress or depress* or anxiety or mental or wellness)))) AND LANGUAGE: (English)

Indexes=SCI-EXPANDED, SSCI Timespan=2010-2019

# 26 723

(TS=(((beach or beaches or coastal or coastline* or seaside) NEAR/3 (health or wellbeing or stress or depress* or anxiety)))) AND LANGUAGE: (English)

Indexes=SCI-EXPANDED, SSCI Timespan=2010-2019

# 25 34

(TS=(((bluespace* or "blue space*") NEAR/3 (health or wellbeing or stress or depress* or anxiety or mental or wellness)))) AND LANGUAGE: (English)

Indexes=SCI-EXPANDED, SSCI Timespan=2010-2019

# 24 1,568

(TS=(((bushland* or countryside or forest* or woodland*) NEAR/3 (health or wellbeing or stress or depress* or anxiety or mental or wellness)))) AND LANGUAGE: (English)

Indexes=SCI-EXPANDED, SSCI Timespan=2010-2019

# 23 391

(TS=(((allotment* or garden or gardens or park or parks or parkland*) NEAR/3 (health or wellbeing or stress or depress* or anxiety or mental or wellness)))) AND LANGUAGE: (English)

Indexes=SCI-EXPANDED, SSCI Timespan=2010-2019

# 22 304

(TS=(((greenspace* or "green space*" or greenery or greenness) NEAR/3 (health or wellbeing or stress or depress* or anxiety or mental or wellness)))) AND LANGUAGE: (English)

Indexes=SCI-EXPANDED, SSCI Timespan=2010-2019

# 21 5,452

#20 OR #12

Indexes=SCI-EXPANDED, SSCI Timespan=2010-2019

# 20 1,740

#19 AND #7

Indexes=SCI-EXPANDED, SSCI Timespan=2010-2019

# 19 817,991

#18 OR #17 OR #16 OR #15 OR #14 OR #13

Indexes=SCI-EXPANDED, SSCI Timespan=2010-2019

# 18 2,474

(TS=(((alleviat* or lessen or manag* or reduc*) NEAR/2 disabilit*))) AND LANGUAGE: (English)

Indexes=SCI-EXPANDED, SSCI Timespan=2010-2019

# 17 23,171

(TS=(((better or enhanc* or improv* or increas*) NEAR/1 "quality of life"))) AND LANGUAGE: (English)

Indexes=SCI-EXPANDED, SSCI Timespan=2010-2019

# 16 10,539

(TS=(((alleviat* or lessen or lower* or manag* or reduc*) NEAR/1 "blood pressure"))) AND LANGUAGE: (English)

Indexes=SCI-EXPANDED, SSCI Timespan=2010-2019

# 15 33,601 (TS=(((alleviat* or decreas* or lessen or manag* or reduc*) NEAR/1 pain))) AND LANGUAGE: (English)

Indexes=SCI-EXPANDED, SSCI Timespan=2010-2019

# 14 5,044

(TS=(((enhanc* or improv* or better) NEAR/1 sleep))) AND LANGUAGE: (English)

Indexes=SCI-EXPANDED, SSCI Timespan=2010-2019

# 13 813,052

(TS=("Cardiovascular Disease" or sleep or "Quality of life" or "Activities of Daily Living" or Hypertension or Pain)) AND LANGUAGE: (English)

Indexes=SCI-EXPANDED, SSCI Timespan=2010-2019

# 12 4,066

#11 AND #7

Indexes=SCI-EXPANDED, SSCI Timespan=2010-2019

# 11 443,253

(#10 OR #9 OR #8) AND LANGUAGE: (English)

Indexes=SCI-EXPANDED, SSCI Timespan=2010-2019

# 10 151,446

(TS=( "anxiety disorders" OR "psychological stress" or "stressful life event*" or resilien* or wellbeing or well-being or "well being")) AND LANGUAGE: (English)

Indexes=SCI-EXPANDED, SSCI Timespan=2010-2019

# 9 242,927

(TS=(depression OR (depressed NEAR/2 mood*) OR "depressive disorder*" OR (low NEAR/2 mood*) OR "Mood Disorders" OR "affective disorder*" )) AND LANGUAGE: (English)

Indexes=SCI-EXPANDED, SSCI Timespan=2010-2019

# 8 123,650

(TS=((mental* NEAR/2 (health or illness)) )) AND LANGUAGE: (English)

Indexes=SCI-EXPANDED, SSCI Timespan=2010-2019

# 7 89,080

#6 OR #5 OR #4 OR #3 OR #2 OR #1

Indexes=SCI-EXPANDED, SSCI Timespan=2010-2019

# 6 22,221

(TS=(((health promot* NEAR/2 environment*) OR ((natural or outdoor* or salutogenic) NEAR/2 environment*) OR (nature or natural) NEAR/2 space*))) AND LANGUAGE: (English)

Indexes=SCI-EXPANDED, SSCI Timespan=2010-2019

# 5 780

(TS=(bluespace* or "blue space*" OR bluehealth OR "blue water*" OR "blue gym*")) AND LANGUAGE: (English)

Indexes=SCI-EXPANDED, SSCI Timespan=2010-2019

# 4 59,901

(TS=(greenery OR greenspace* OR garden* OR park or parks or parkland* OR "sports field*" OR "wilderness area*" OR "public open space*" OR "neighbourhood open space*" OR "neighborhood open space*")) AND LANGUAGE: (English)

Indexes=SCI-EXPANDED, SSCI Timespan=2010-2019

# 3 264

(TS=((ambient or city or cities or environment* or neighbourhood or neighborhood or residential or surrounding or urban) NEAR/2 greenness)) AND LANGUAGE: (English)

Indexes=SCI-EXPANDED, SSCI Timespan=2010-2019

# 2 5,279

(TS=((city or cities or environment* or neighbourhood or neighborhood or urban) NEAR/2 greening)) AND LANGUAGE: (English)

Indexes=SCI-EXPANDED, SSCI Timespan=2010-2019

# 1 8,631

(TS=(green NEAR/2 (area* or cover or environment* or gym* or neighbourhood* or neighborhood* or roadside* or space*))) AND LANGUAGE: (English)

Indexes=SCI-EXPANDED, SSCI Timespan=2010-2019

**Science Citation Index Search 2**

# 33 4,960

(#31 not #32) AND LANGUAGE: (English)

Indexes=SCI-EXPANDED, SSCI Timespan=2010-2019

# 32 624,515

(TI=(rat or rats or mouse or mice or bird or birds or cow or cattle or bovine or sheep or goat* or ovine or horse or equine or pig or pigs or porcine or fish or fishes or bird or birds or avian or insect or insects)) AND LANGUAGE: (English)

Indexes=SCI-EXPANDED, SSCI Timespan=2010-2019

# 31 5,029

(#29 not #30) AND LANGUAGE: (English)

Indexes=SCI-EXPANDED, SSCI Timespan=2010-2019

# 30 2,461,088

(TI=((( (clinical or surgery or surgical or cell or cells or laboratory or placebo or bladder or uterus or breast or gene or genes or genetic or bowel or liver or enzymes or viral or lymph or molecular) )))) AND LANGUAGE: (English)

Indexes=SCI-EXPANDED, SSCI Timespan=2010-2019

# 29 5,095

#28 AND #27

Indexes=SCI-EXPANDED, SSCI Timespan=2010-2019

# 28 1,294,582

(TS=(((Health or (quality NEAR/2 life) or (well NEAR/1 being) or wellbeing)))) AND LANGUAGE: (English)

Indexes=SCI-EXPANDED, SSCI Timespan=2010-2019

# 27 23,667

#26 OR #22 OR #17 OR #14 OR #11

Indexes=SCI-EXPANDED, SSCI Timespan=2010-2019

# 26 9,032

#25 OR #24 OR #23

Indexes=SCI-EXPANDED, SSCI Timespan=2010-2019

# 25 2,793

(TS=(((((communit* or local) NEAR/2 (garden* or park or parks or parkland* or green* or greenspace or outdoor* or outside* or pavement* or sidewalk* or wood* or allotment* or lake* or canal* or river*)) and (work* or renewal or volunteer* or voluntary or practical or participat* or regenerat* or restor* or maintain* or enhance or preserve or creat*))))) AND LANGUAGE: (English)

Indexes=SCI-EXPANDED, SSCI Timespan=2010-2019

# 24 4,786

(TS=((((communit* and (work* or renewal or volunteer* or voluntary or practical or regenerat* or restor* or maintain* or care or enhance* or preserve or creat* or activ* or action* or involve*) and (("natur* environment*") or (environmental* and conservation*))) and (Regenerat* or restore or restoration or redevelop or maintain or enhance or preserve or preserving or create or creation or establish or establishing or founding or build* or cultivat* or cultivation or participati* or practical or creat* or activ* or action* or involve*))))) AND LANGUAGE: (English)

Indexes=SCI-EXPANDED, SSCI Timespan=2010-2019

# 23 2,006

(TS=(((((communit* NEAR/2 (group* or team* or association* or organisation or organization or participa* or stakeholder* or steward* or trust* or ranger* or activit*)) and (garden* or allotment* or forest or (natural and environment) or conservation*)) and (Regenerat* or restore or restoration or redevelop or maintain or enhance or preserve or preserving or create or creation or establish or establishing or founding or build* or cultivat* or cultivation or participati* or practical or creat* or activ* or action* or involve*))))) AND LANGUAGE: (English)

Indexes=SCI-EXPANDED, SSCI Timespan=2010-2019

# 22 2,837

#21 OR #20 OR #19 OR #18

Indexes=SCI-EXPANDED, SSCI Timespan=2010-2019

# 21 841

(TS=((((garden* or horticulture or allotment* or botanical or arboretum) NEAR/2 (renew* or maintain* or creat* or culivat* or enhance* or restore or regenerat* or activ* or preserve or voluntary or volunteer or conservation* or participat*))))) AND LANGUAGE: (English)

Indexes=SCI-EXPANDED, SSCI Timespan=2010-2019

# 20 1

(TS=((((garden* or horticulture or allotment* or botanical or arboretum) and (pick and (your own)))))) AND LANGUAGE: (English)

Indexes=SCI-EXPANDED, SSCI Timespan=2010-2019

# 19 7

(TS=((((garden* or horticulture or allotment* or botanical or arboretum) and (grow and (your own)))))) AND LANGUAGE: (English)

Indexes=SCI-EXPANDED, SSCI Timespan=2010-2019

# 18 2,198

(TS=((((garden* or horticulture or allotment* or botanical or arboretum) NEAR/2 (kitchen or school* or college* or university or campus or hospital* or prison* or penitentiary or institution or urban or green* or communit* or communal or group* or guerrilla or (bio NEAR/1 diver*) or eco))))) AND LANGUAGE: (English)

Indexes=SCI-EXPANDED, SSCI Timespan=2010-2019

# 17 8,183

#16 OR #15

Indexes=SCI-EXPANDED, SSCI Timespan=2010-2019

# 16 3,696

(TS=((((work* or renewal or volunteer* or voluntary or practical or regenerat* or restor* or maintain* or care or enhance or preserve or creat*) and (urban or city or metropolis or town*) and (garden* or park* or parkland or allotment*))))) AND LANGUAGE: (English)

Indexes=SCI-EXPANDED, SSCI Timespan=2010-2019

# 15 5,565

Indexes= Timespan=2010-2019

# 14 6,292

#13 OR #12

Indexes=SCI-EXPANDED, SSCI Timespan=2010-2019

# 13 482

(TS=((greenspace))) AND LANGUAGE: (English)

Indexes=SCI-EXPANDED, SSCI Timespan=2010-2019

# 12 5,992

(TS=(((Green* NEAR/2 (space* or gym or exercise or volunteer* or voluntary or conservation or infrastructure or care or streets or communal or Guerrilla))))) AND LANGUAGE: (English)

Indexes=SCI-EXPANDED, SSCI Timespan=2010-2019

# 11 1,204

#10 OR #9

Indexes=SCI-EXPANDED, SSCI Timespan=2010-2019

# 10 390

(TS=((((((voluntary or volunteer*) NEAR/2 (group* or association or stakeholder* or steward* or ranger*)) and (environment* or nature or rural or countryside or outdoor* or outside or backcountry or hinterland or outback or wood* or park or parks or parkland* or garden* or meadow* or farm* or ("farm land") or horticultural or floricultural or botanical or arboretum or allotment* or forest* or rainforest or moor* or dale* or marsh* or mountain* or beach* or wilderness or landscape* or tree* or copse* or river* or lake* or canal* or waterway or wetland* or ("open space*") or ("protected area*") or green* or planning* or footpath* or trail* or coast* or cliff* or dune* or ("bio diversity") or ("eco system") or ("protected area*"))) and (Regenerat* or restore or restoration or redevelop or maintain or enhance or preserve or preserving or create or creation or establish or establishing or founding or build* or cultivat* or cultivation or participati* or practical or creat* or activ* or action* or involve*))))) AND LANGUAGE: (English)

Indexes=SCI-EXPANDED, SSCI Timespan=2010-2019

# 9 856

(TS=(((((volunteer* or voluntary) NEAR/2 (environment* or nature or rural or countryside or outdoor* or outside or backcountry or hinterland or outback or wood* or park or parks or parkland* or garden* or meadow* or farm* or ("farm land") or horticultural or floricultural or botanical or arboretum or allotment* or forest* or rainforest or moor* or dale* or marsh* or mountain* or beach* or wilderness or landscape* or tree* or copse* or river* or lake* or canal* or waterway or wetland* or ("open space*") or ("protected area*") or green* or planning* or footpath* or trail* or coast* or cliff* or dune* or ("bio diversity") or ("eco system") or ("protected area*"))) and (Regenerat* or restore or restoration or redevelop or maintain or enhance or preserve or preserving or create or creation or establish or establishing or founding or build* or cultivat* or cultivation or participati* or practical or creat* or activ* or action* or involve*))))) AND LANGUAGE: (English)

Indexes=SCI-EXPANDED, SSCI Timespan=2010-2019

# 8 18,008

#7 OR #6 OR #5 OR #4 OR #3 OR #2 OR #1

Indexes=SCI-EXPANDED, SSCI Timespan=2010-2019

# 7 1,768

(TS=((((activ* or practical or participat*) NEAR/2 conservation*)))) AND LANGUAGE: (English)

Indexes=SCI-EXPANDED, SSCI Timespan=2010-2019

# 6 241

(TS=(((geoconservation or (geo NEAR/2 conservation))))) AND LANGUAGE: (English)

Indexes=SCI-EXPANDED, SSCI Timespan=2010-2019

# 5 9,998

(TS=((((conservation* NEAR/2 (nature or rural or countryside or outdoor* or outside or backcountry or hinterland or outback or wood* or park or parks or parkland or garden* or meadow* or farm* or ("farm land") or horticultural or floricultural or botanical or arboretum or allotment* or forest* or rainforest or moor* or dale* or marsh* or mountain* or beach* or wilderness or landscape* or tree* or copse* or river* or lake* or canal* or waterway or wetland* or ("open space*") or ("protected area*") or green* or planning* or footpath* or trail* or coast* or cliff* or dune* or ("bio diversity") or ("eco system") or ("protected area*"))) and (Regenerat* or restore or restoration or redevelop or maintain or enhance or preserve or preserving or create or creation or establish or establishing or founding or build* or cultivat* or cultivation or participati* or practical or creat* or activ* or action* or involve*))))) AND LANGUAGE: (English)

Indexes=SCI-EXPANDED, SSCI Timespan=2010-2019

# 4 2,216

(TS=((((conservation* NEAR/2 (group* or volunteer* or voluntary or association* or organisation* or organization* or participa* or stakeholder* or steward* or trust or ranger* or activit*)) AND (Regenerat* or restore or restoration or redevelop or maintain or enhance or preserve or preserving or create or creation or establish or establishing or founding or build* or cultivat* or cultivation or participati* or practical or creat* or activ* or action* or involve*))))) AND LANGUAGE: (English)

Indexes=SCI-EXPANDED, SSCI Timespan=2010-2019

# 3 1,682

(TS=((((environmental* NEAR/2 (conservation* or volunteer* or steward*)) AND (Regenerat* or restore or restoration or redevelop or maintain or enhance or preserve or preserving or create or creation or establish or establishing or founding or build* or cultivat* or cultivation or participati* or practical or creat* or activ* or action* or involve*))))) AND LANGUAGE: (English)

Indexes=SCI-EXPANDED, SSCI Timespan=2010-2019

# 2 593

(TS=(((Conservation NEAR/2 interventions)))) AND LANGUAGE: (English)

Indexes=SCI-EXPANDED, SSCI Timespan=2010-2019

# 1 5,060

(TS=(((conservation* and natural and environment* and (renewal or volunteer* or voluntary or participat* or practical or regenerat* or restor* or maintain* or care or enhance* or preserve or creat* or activ* or action* or involve*))))) AND LANGUAGE: (English)

Indexes=SCI-EXPANDED, SSCI Timespan=2010-2019
