# Supplementary material for: Nature-based outdoor activities for mental and physical health: Systematic review and meta-analysis
Source: SSM Popul Health. 2021 Oct 1;16:100934. doi: 10.1016/j.ssmph.2021.100934 (PMC8498096; doi:10.1016/j.ssmph.2021.100934)
Supplement: Multimedia component 2 [file mmc2.docx]

**Table B.1. Characteristics of included studies**

| **Author (year)** | **Country** | **Design** | **Population** | **Mean age (years, SD)** | **% Female** | **Sample size (n)** | **Risk of bias (overall)** |
| --- | --- | --- | --- | --- | --- | --- | --- |
| Bail et al. (2018) | USA | RCT | Physical health problems/LTC | 60.5 (9.4) | 100 | 82 | Moderate |
| Bang et al. (2017) | South Korea | Controlled study | University student volunteers | 24.3 (4.19) | 52 | 118 | Moderate |
| Barton et al. (2010) | UK | Cross over Controlled study | Common mental health problems | 53.0 (15.4) | 62 | 93 | Moderate |
| Bay-Richter et al. (2012) | Sweden | RCT | Common mental health problems | NR | NR | NR | Moderate |
| Berman et al. (2012) | USA | Cross-over Controlled study | Common mental health problems | 26.0 | 60 | 40 | Moderate |
| Bettmann et al. (2017) | USA | Single group | Common mental health problems and SMI | 20.2 years (2.3) | 35.3 | 157 | Moderate |
| Bielinis et al. (2021) | Poland | RCT | Healthy adult volunteers | 21.4 | 41.9 | 62 | Moderate |
| Brown et al. (2014) | UK | RCT | Healthy adult volunteers | 42.0 (10.6) | 21.3 | 46 | Moderate |
| Brown et al. (2020) | USA | RCT | Physical health problems/LTC | NR | 80 | 18 | Moderate |
| Calogiuri et al. (2016) | Norway | Cross over RCT | Healthy adult volunteers | 49 (8.0) | 50 | 14 | Low |
| Coventry et al. (2019) | UK | Single group | Healthy adult volunteers/CMD/SMI | 43.8 (2.83) | 41 | 45 | Moderate |
| de Brito et al. (2019) | USA | Controlled study | Healthy adult volunteers | 49.3 (6.7) | 83 | 23 | Moderate |
| Furuyashiki et al. (2019) | Japan | Single group | Healthy adult volunteers | 44.0 (9.6) | 55.5 | 155 | Moderate |
| Gerber et al. (2017) | USA | Controlled study | Healthy adult volunteers | 44.6 (15.0) | 62 | 50 | Moderate |
| Gidlow et al. (2016) | UK | RCT | Healthy adult volunteers | 40.9 (17.6) | 39.5 | 57 | Low |
| Gonzalez et al. (2011) | Norway | Single group | CMD/SMI | 46.3 (11.6) | 78.3 | 28 | Moderate |
| Hall et al. (2018) | Canada | Single group | Physical health problems/LTC | 84.0 | 28.6 | 25 | High |
| Han et al. (2018) | Taiwan | RCT | University student volunteers | 20.8 (1.1) | 55.7 | 116 | Low |
| Hawkins et al. (2015) | Wales | Controlled study | Healthy adult volunteers | 67.0 (8.5) | 68.1 | 94 | Moderate |
| Holt et al. (2019) | USA | Controlled study | University student volunteers | NR | 69.6 | 207 | Moderate |
| Iwata et al. (2016) | Ireland | Single group | SMI | 47.0 | 80 | 13 | High |
| Johansson et al. (2011) | Sweden | Controlled study | University student volunteers | 23.3 (2.92) | 50 | 40 | Moderate |
| Kling et al. (2018) | USA | Single group | Healthy adult volunteers | NR | 50.9 | 106 | Moderate |
| Korpela et al. (2016) | Finland | Single group | CMD | 48.0 | 56 | 16 | Moderate |
| Lanki et al. (2017) | Finland | Controlled study | Healthy adult volunteers | 46.0 (8.7) | 100 | 60 | Moderate |
| Lee et al. (2011) | Japan | Controlled study | University student volunteers | 21.1 (1.2) | 0 | 24 | High |
| Lee et al. (2014) | Japan | Controlled study | Healthy adult volunteers | 21.0 (0.9) | 0 | 96 | Moderate |
| Lucke et al. (2019) | Germany | Controlled study | Healthy adult volunteers | 46.0 | 75 | 64 | High |
| Mackay et al. (2010) | Australia | Single group | Healthy adult volunteers | 43.8 (16.6) | 41 | 16 | High |
| Marselle et al. (2013) | UK | Controlled study | Healthy adult volunteers | NR | 62 | 260 | Moderate |
| Marselle et al. (2016) | UK | Single group | Healthy adult volunteers | NR | 55.5 | 127 | Moderate |
| Martens et al. (2011) | Switzerland | RCT | Healthy adult volunteers/university students | 37.0 | 55 | 96 | Low |
| McCaffrey et al. (2016) | USA | Single group | Healthy adult volunteers | NR | 57 | 195 | High |
| Mourão et al. (2019) | Portugal | Single group | Healthy adult volunteers | NR | 43 | 65 | High |
| Müller-Riemenschneider et al. (2020) | Singapore | RCT | Healthy adult volunteers | 51.1 (6.3) | 79 | 160 | Low |
| Ng et al. (2018) | Singapore | RCT | Healthy adult volunteers | 67.1 (4.3) | 78 | 59 | Low |
| Oh et al. (2018) | South Korea | Controlled study | SMI | 37.7 (11.2) | 28 | 28 | High |
| Olafsdottir et al. (2020) | Iceland | RCT | University student volunteers | 24.39 (2.61) | 68 | 60 | Low |
| Sin-Ae et al. (2016) | South Korea | Controlled study | Healthy adult volunteers | 81.9 (5.1) | 100 | 50 | High |
| Sin-Ae et al. (2017) | South Korea | Controlled study | Healthy adult volunteers | 80.6 (5.1) | 100 | 21 | Moderate |
| Song et al. (2015) | Japan | Cross over RCT | Physical health problems/LTC | 21.7 (1.6) | 0 | 20 | Low |
| Song et al. (2018) | Japan | Cross over RCT | Healthy adult volunteers | 58.0 (10.6) | 0 | 585 | Moderate |
| Van Den Berg et al. (2011) | The Netherlands | RCT | Healthy adult volunteers | 57.6 | 73 | 30 | Low |
| Vujcic et al. (2017) | Serbia | RCT | Common mental health problems | 45.3 (0.16) | 70 | 30 | Low |
| Warber et al. (2015) | USA | Single group | Healthy adult volunteers | 19 | 67 | 36 | High |
| Wilson et al. (2011) | UK | Single group | CMD/SMI | 42.6 (10.6) | 18.2 | 77 | High |
| Wood et al. (2016) | UK | Controlled study | Healthy adult volunteers | 55.6 (13.6) | 44 | 269 | Moderate |
| Wyles et al. (2017) | UK | Single group | University student volunteers | 22 (6.18) | 75.6 | 30 | Moderate |
| Yao et al. (2017) | Taiwan | Controlled study | Physical health problems/LTC | 79.9 (8.03) | 51 | 85 | Moderate |
| Zeng et al. (2020)* | China | Controlled study | University student volunteers | 21.8 (0.8) | 50 | 120 | Moderate |

CMD = common mental disorders; LTC = long-term condition; RCT = randomised controlled trial; SMI = serious mental illness.

*Linked paper: Lyu et al. (2019)

**Table B.2 Characteristics of interventions**

| **Author (year)** | **Intervention components** | **Intervention category** | **Control** | **Green/blue space** | **Individual/Group** | **Facilitated** | **Duration (weeks)** | **Frequency and dose** |
| --- | --- | --- | --- | --- | --- | --- | --- | --- |
| Bail et al. (2018) | Mentored vegetable gardening | Gardening | Wait-list | Green | Individual | Yes | 52 | NR |
| Bang et al. (2017) | Campus forest walks | Green exercise | Daily routine | Green | Group | No | 6 | 1 hour per week |
| Barton et al. (2010) | Walks in public green space | Green exercise | Indoor swimming | Green | Group | Yes | 6 | 45 minutes per week |
| Bay-Richter et al. (2012) | Garden therapeutic activities | Gardening | TAU | Green | Group | NR | 8 | NR |
| Berman et al. (2012) | Walk in public green space | Green exercise | Urban walk | Green | Individual | No | 1 | 55 minutes |
| Bettmann et al. (2017) | Wilderness therapy | Nature-based therapy | None | Green | Individual | Yes | 7 | Every day |
| Bielinis et al. (2021) | Forest bathing | Nature-based therapy | Urban control | Green | Group | No | 1 | NR |
| Brown et al. (2014) | Walk in treelined and grass maintained footpaths and country lanes | Green exercise | Wait-list | Green | Group | No | 8 | 20 minutes, twice a week |
| Brown et al. (2020) | Community based gardening | Gardening | Inactive control | Green | Group | Yes | 20 | 90 minutes, twice a month |
| Calogiuri et al. (2016) | Outdoor exercise in green space | Green exercise | Indoor exercise | Green | Group | Yes | 2 | 45 minutes per week |
| Coventry et al. (2019) | a) Guided walks in a nature reserve;  b) Conservation volunteering | Green exercise/conservation | None | Green | Group | Yes | 8 | 20-30 minutes per week |
| de Brito et al. (2019) | Walk along unpaved woodland trail | Green exercise | Urban walk | Green | Individual | No | 3 | Once a week |
| Furuyashiki et al. (2019) | Forest bathing | Nature-based therapy | None | Green | Group | Yes | 1 | 4 hours |
| Gerber et al. (2017) | Community based gardening | Gardening | Non-gardening group | Green | Group | NR | NR | NR |
| Gidlow et al. (2016) | a) Walk in country park  b) Walk along canal footpath | Green exercise | Urban walk | Green/Blue | Individual | No | 3 | 30 minutes per week |
| Gonzalez et al. (2011) | Therapeutic horticulture | Gardening | None | Green | Group | Yes | 12 | 3 hours, twice a week |
| Hall et al. (2018) | Therapeutic horticulture | Gardening | None | Green | Group | Yes | 10 | 2 hours per week |
| Han et al. (2018) | Walk along vegetation lined road | Green exercise | Urban exercise | Green | Individual | No | 1 | 15 minutes |
| Hawkins et al. (2015) | Allotment gardening | Gardening | Indoor exercise | Green | Group | NR | NR | NR |
| Holt et al. (2019) | Physical activity in campus green space | Green exercise | Inactive control | Green/Blue | Individual | NR | NR | NR |
| Iwata et al. (2016) | Woodland walk | Green exercise | None | Green | Group | No | 13 | 2 hours per week |
| Johansson et al. (2011) | Walk in public green space | Green exercise | a) Street walk -alone  b) Street walk - friend | Green | Individual and group | No | 1 | 40 minutes |
| Kling et al. (2018) | Park based fitness classes | Green exercise | None | Green | Group | Yes | 21 | 1 hour, 2 to 3 times per week |
| Korpela et al. (2016) | Urban woodland walk | Green exercise | None | Green | Group | Yes | 8 | 1 hour per week |
| Lanki et al. (2017) | a) Urban forest walks  b) Urban park walk | Green exercise | Urban walk | Green | Individual | NR | NR | 45 minutes per week |
| Lee et al. (2011) | Forest bathing | Nature-based therapy | Urban walk | Green | Individual | No | 1 | NR |
| Lee et al. (2014) | Forest walk | Green exercise | Urban walk | Green | Individual | NR | 1 | NR |
| Lucke et al. (2019) | Outdoor mindfulness | Nature-based therapy | Wait-list | Green | Group | Yes | 16 | One half day plus two evenings per week |
| Mackay et al. (2010) | a) Run in suburban bushland  b) Orienteering walking in suburban bushland | Green exercise | None | Green/Blue | Group | No | NR |  |
| Marselle et al. (2013) | Public and urban green space walks; coastal walks | Green exercise | Urban walk | Green/Blue | Group | NR | 13 | NR |
| Marselle et al. (2016) | Public and urban green space walks; coastal walks | Green exercise | None | Green/Blue | Group | No | 13 | NR |
| Martens et al. (2011) | Tended urban forest walk | Green exercise | Non-modified wild forest | Green | Individual | No | 13 | NR |
| McCaffrey et al. (2016) | Guided garden walk | Green exercise | None | Green | Individual | No | 6 | 30 minutes |
| Mourão et al. (2019) | Allotment gardening | Gardening | None | Green | Individual | No | 6 | NR |
| Müller-Riemenschneider et al. (2020) | Structured physical exercise in public park | Green exercise | Standard physical activity materials | Green | Group | Yes | 24 | Weekly |
| Ng et al. (2018) | Horticultural therapy | Gardening | Wait-list | Green | Group | NR | 24 | Weekly |
| Oh et al. (2018) | Horticultural therapy | Gardening | TAU | Green | Group | NR | 24 | 1 hour per weekly for 3 months; 1 hour per month for next 3 months |
| Olafsdottir et al. (2020) | Woodland walk | Green exercise | a) Indoor exercise  b) Viewing nature on television | Green | Group | Yes | 12 | Once a week |
| Sin-Ae et al. (2016) | Seasonal plant growing | Gardening | TAU | Green | Group | NR | 7.5 | 15 sessions, 50 minutes per session, twice a week |
| Sin-Ae et al. (2017) | Seasonal plant growing | Gardening | TAU | Green | Group | Yes | 7.5 | 15 sessions, 50 minutes, twice a week |
| Song et al. (2015) | Forest walk | Green exercise | Urban walk | Green | Individual | Yes | 12 | 50 minutes, twice a week |
| Song et al. (2018) | Forest walk | Green exercise | Urban walk | Green | Individual | NR | 1 | 17 minutes |
| Van Den Berg et al. (2011) | Light gardening activities | Gardening | Indoor reading | Green | Individual | NR | 1 | 15 minutes |
| Vujcic et al. (2017) | Horticultural therapy in botanical gardens | Gardening | Occupational art therapy | Green | Group | No | 2 | 30 minutes |
| Warber et al. (2015) | Wilderness therapy | Nature-based therapy | None | Green | Group | Yes | 4 | NR |
| Wilson et al. (2011) | Ecotherapy (conservation; bushcraft; environmental art) | Nature-based therapy | None | Green | Group | Yes | 4 | NR |
| Wood et al. (2016) | Allotment gardening | Gardening | None | Green | Group | Yes | 12 | 3 hours per week |
| Wyles et al. (2017) | Volunteer beach clean | Conservation | None | Blue | Group | NR | NR | NR |
| Yao et al. (2017) | Horticultural therapy | Gardening | Inactive control | Green | Group | NR | 2 | 90 minutes |
| Zeng et al. (2020)* | Bamboo forest therapy | Nature-based therapy | City site | Green | Group | Yes | 8 | 60 minutes, once a week |

NR = not reported; TAU = treatment as usual.

*Linked paper: Lyu et al. (2019)

**Table B.3 Risk of bias of randomised controlled trials**

| **Author (year)** | **Random sequence generation** | **Allocation concealment** | **Blinding of outcome assessors** | **Incomplete outcome data** | **Selective reporting** |
| --- | --- | --- | --- | --- | --- |
| Bail et al. (2018) | Low | Unclear | High | Low | Low |
| Bay-Richter et al. (2012) | Unclear | Unclear | Unclear | Unclear | Low |
| Bielinis et al. (2021) | Unclear | Unclear | High | Low | Low |
| Brown et al. (2014) | Low | Unclear | Low | Low | Low |
| Brown et al. (2020) | Unclear | Unclear | Low | Unclear | Low |
| Calogiuri et al. (2016) | Unclear | Unclear | Low | Low | Low |
| Gidlow et al. (2016) | Low | Low | Unclear | Low | Low |
| Han et al. (2018) | Unclear | Unclear | Unclear | Low | Low |
| Martens et al. (2011) | Unclear | Unclear | Unclear | Low | Low |
| Müller-Riemenschneider et al. (2020) | Low | Low risk | Unclear | Low | Low |
| Ng et al. (2018) | Unclear | Unclear | Low | Low | Low |
| Olafsdottir et al. (2020) | Unclear | Unclear | Low | Low | Low |
| Song et al. (2015) | Unclear | Unclear | Unclear | Low | Low |
| Song et al. (2018) | Unclear | Unclear | Unclear | Low | Unclear |
| Van Den Berg et al. (2011) | Unclear | Unclear | Unclear | Low | Low |
| Vujcic et al. (2017) | Unclear | Low risk | Unclear | Unclear | Low |

**Table B.4 Risk of bias of non-randomised controlled trial**

|  | Bang et al. (2017) | Barton et al. (2010) | Berman et al. (2012) | Bettmann et al. (2017) | Coventry et al. (2019) | de Brito et al. (2019) | Furuyashiki et al. (2019) | Gerber et al. (2017) | Gonzalez et al. (2011) | Hall et al. (2018) | Hawkins et al. (2015) | Holt et al. (2019) | Iwata et al. (2016) | Johansson et al. (2011) | Kling et al. (2018) | Korpela et al. (2016) | Lanki et al. (2017) |
| --- | --- | --- | --- | --- | --- | --- | --- | --- | --- | --- | --- | --- | --- | --- | --- | --- | --- |
| **Section 1: Population** |  |  |  |  |  |  |  |  |  |  |  |  |  |  |  |  |  |
| 1.1 Is the source population or source area well described? | + | ++ | ++ | + | ++ | ++ | ++ | ++ | + | + | + | + | + | - | - | + | ++ |
| 1.2 Is the eligible population or area representative of the source population or area? | + | + | ++ | + | + | + | + | + | + | + | + | + | - | - | - | + | + |
| 1.3 Do the selected participants or areas represent the eligible population or area? | ++ | + | + | - | + | + | - | + | - | - | + | + | - | - | - | + | + |
| **Section 2: Method of selection of exposure (or comparison) group** |  |  |  |  |  |  |  |  |  |  |  |  |  |  |  |  |  |
| 2.1 Selection of exposure (and comparison) group. How was selection bias minimised? | - | - | + | - | - | - | - | - | - | - | - | + | - | - | - | - | + |
| 2.2 Was the selection of explanatory variables based on a sound theoretical basis? | ++ | - | ++ | + | ++ | ++ | + | ++ | + | - | - | + | - | - | - | ++ | + |
| 2.3 Was the contamination acceptably low? | NR | NA | NA | NA | NR | NR | NA | NR | NA | NA | NR | NA | NA | NA | NA | NA | + |
| 2.4 How well were likely confounding factors identified and controlled? | - | - | - | - | - | + | - | - | - | - | - | - | - | - | + | - | + |
| 2.5 Is the setting applicable to the UK? | Yes | Yes | Yes | Yes | Yes | Yes | Yes | Yes | Yes | No | Yes | Yes | Yes | Yes | Yes | Yes | Yes |
| **Section 3: Outcomes** |  |  |  |  |  |  |  |  |  |  |  |  |  |  |  |  |  |
| 3.1 Were the outcome measures and procedures reliable? | ++ | ++ | ++ | ++ | ++ | ++ | ++ | ++ | ++ | - | + | ++ | ++ | + | ++ | ++ | ++ |
| 3.2 Were the outcome measurements complete? | NR | ++ | NR | + | NR | + | ++ | NR | NR | NR | + | + | NR | NR | ++ | + | + |
| 3.3 Were all the important outcomes assessed? | ++ | + | + | + | + | + | + | + | + | - | + | + | + | + | + | + | + |
| 3.4 Was there a similar follow-up time in exposure and comparison groups? | ++ | NA | NA | NA | + | + | NA | + | NA | NA | + | + | NA | NA | NA | NA | + |
| 3.5 Was follow-up time meaningful? | ++ | + | + | + | + | + | NR | ++ | + | - | - | + | + | NR | ++ |  | + |
| **Section 4: Analyses** |  |  |  |  |  |  |  |  |  |  |  |  |  |  |  |  |  |
| 4.1 Was the study sufficiently powered to detect an intervention effect (if one exists)? | NA | NA | NA | NA | NA | NA | NA | NA | NA | NA | NA | NA | NA | NA | NA | NA | NA |
| 4.2 Were multiple explanatory variables considered in the analyses? | + | + | + | + | + | + | + | - | + | - | - | + | - | - | ++ | - | ++ |
| 4.3 Were the analytical methods appropriate? | + | + | + | + | + | + | + | - | + | - | + | + | - | + | ++ | + | ++ |
| 4.4 Was the precision of association given or calculable? Is association meaningful? | + | + | + | ++ | ++ | ++ | + | - | - | - | + | + | - | + | ++ | ++ | + |
| **Section 5: Summary** |  |  |  |  |  |  |  |  |  |  |  |  |  |  |  |  |  |
| 5.1 Are the study results internally valid (i.e. unbiased)? | + | + | + | + | + | + | + | - | + | - | + | + | - | - | + | + | + |
| 5.2 Are the findings generalisable to the source population (i.e. externally valid)? | + | + | + | + | + | + | + | - | + | - | + | + | - | - | + | + | + |

|  | Lee et al. (2011) | Lee et al. (2014) | Lucke et al. (2019) | Mackay et al. (2010) | Marselle et al. (2013) | Marselle et al. (2016) | McCaffrey et al. (2016) | Mourão et al. (2019) | Oh et al. (2018) | Sin-Ae et al. (2016) | Sin-Ae et al. (2017) | Warber et al. (2015) | Wilson et al. (2011) | Wood et al. (2016) | Wyles et al. (2017) | Yao et al. (2017) | Zeng et al. (2020)* |
| --- | --- | --- | --- | --- | --- | --- | --- | --- | --- | --- | --- | --- | --- | --- | --- | --- | --- |
| **Section 1: Population** |  |  |  |  |  |  |  |  |  |  |  |  |  |  |  |  |  |
| 1.1 Is the source population or source area well described? | - | - | - | + | + | + | - | - | - | + | + | + | - | + | + | + | + |
| 1.2 Is the eligible population or area representative of the source population or area? | - | - | - | - | + | + | - | - | - | - | - | + | - | + | - | - | - |
| 1.3 Do the selected participants or areas represent the eligible population or area? | - | - | - | - | - | - | - | - | - | - | - | - | - | + | - | - | - |
| **Section 2: Method of selection of exposure (or comparison) group** |  |  |  |  |  |  |  |  |  |  |  |  |  |  |  |  |  |
| 2.1 Selection of exposure (and comparison) group. How was selection bias minimised? | - | - | - | - | - | - | - | - | - | - | - | - | - | + | - | - | - |
| 2.2 Was the selection of explanatory variables based on a sound theoretical basis? | + | + | + | + | ++ | ++ | - | - | + | + | + | + | + | + | + | + | + |
| 2.3 Was the contamination acceptably low? | NR | NR | NR | NR | NR | NA | NA | NA | NR | NR | NR | NA | NA | NR | NR | + | NR |
| 2.4 How well were likely confounding factors identified and controlled? | - | - | - | - | ++ | - | - | - | - | - | - | - | - | + | - | - | + |
| 2.5 Is the setting applicable to the UK? | Yes | Yes | Yes | Yes | Yes | Yes | Yes | Yes | Yes | Yes | Yes | Yes | Yes | Yes | Yes | Yes | No |
| **Section 3: Outcomes** |  |  |  |  |  |  |  |  |  |  |  |  |  |  |  |  |  |
| 3.1 Were the outcome measures and procedures reliable? | + | + | + | ++ | ++ | ++ | + | + | ++ | ++ | ++ | + | + | ++ | + | + | + |
| 3.2 Were the outcome measurements complete? | NR | NR | - | NR | + | + | + | - | + | + | + | + | + | + | + | + | + |
| 3.3 Were all the important outcomes assessed? | - | - | + | + | + | + | + | - | + | + | + | + | + | + | + | + | + |
| 3.4 Was there a similar follow-up time in exposure and comparison groups? | + | + | - | NR | + | NA | NA | - | + | + | + | NA | NA | NR | + | + | NR |
| 3.5 Was follow-up time meaningful? | - | - | + | - | + | + | - | - | + | + | + | NR | + | NR | + | + | + |
| **Section 4: Analyses** |  |  |  |  |  |  |  |  |  |  |  |  |  |  |  |  |  |
| 4.1 Was the study sufficiently powered to detect an intervention effect (if one exists)? | NA | NA | NA | NA | NA | NA | NA | NA | NA | NA | NA | NA | + | NR | NA | + | NA |
| 4.2 Were multiple explanatory variables considered in the analyses? | - | - | - | - | ++ | ++ | - | - | - | - | - | - | - | + | - | - | - |
| 4.3 Were the analytical methods appropriate? | + | + | + | + | ++ | ++ | - | - | - | - | - | - | - | + | + | + | + |
| 4.4 Was the precision of association given or calculable? Is association meaningful? | ++ | + | - | + | + | + | + | - | - | + | + |  | + | ++ | + | + | + |
| **Section 5: Summary** |  |  |  |  |  |  |  |  |  |  |  |  |  |  |  |  |  |
| 5.1 Are the study results internally valid (i.e. unbiased)? | - | + | - | - | + | + | - | - | - | - | - | - | - | + | + | + | + |
| 5.2 Are the findings generalisable to the source population (i.e. externally valid)? | - | + | - | - | + | + | - | - | - | - | - | - | - | + | + | + | + |

++ Minimised sources of bias; + Either unclear or not all potential sources of bias addressed; - Significant sources of bias; NA= Not applicable; NR = Not reported; *Linked study: Lyu et al. (2019)s

**References**

Bail, J. R., Fruge, A. D., Cases, M. G., et al. (2018). A home-based mentored vegetable gardening intervention demonstrates feasibility and improvements in physical activity and performance among breast cancer survivors. *Cancer, 124*(16), 3427-3435. doi:10.1002/cncr.31559

Bang, K. S., Lee, I., Kim, S., et al. (2017). The Effects of a Campus Forest-Walking Program on Undergraduate and Graduate Students' Physical and Psychological Health. *Int J Environ Res Public Health, 14*(7). doi:10.3390/ijerph14070728

Barton, J., & Pretty, J. (2010). What is the best dose of nature and green exercise for improving mental health? A multi-study analysis. *Environ Sci Technol, 44*(10), 3947-3955. doi:10.1021/es903183r

Bay-Richter, C., Träskman-Bendz, L., Grahn, P., & Brundin, L. (2012). Garden rehabilitation stabilises INF-gamma and IL-2 levels but does not relieve depressive-symptoms. *Neurology Psychiatry and Brain Research, 18*, 37.

Berman, M. G., Kross, E., Krpan, K. M., et al. (2012). Interacting with nature improves cognition and affect for individuals with depression. *J Affect Disord, 140*(3), 300-305. doi:10.1016/j.jad.2012.03.012

Bettmann, J. E., Tucker, A., Behrens, E., & Vanderloo, M. (2017). Changes in Late Adolescents and Young Adults’ Attachment, Separation, and Mental Health During Wilderness Therapy. *Journal of Child and Family Studies, 26*(2), 511-522. doi:10.1007/s10826-016-0577-4

Bielinis, E., Janeczko, E., Takayama, N., et al. (2021). The effects of viewing a winter forest landscape with the ground and trees covered in snow on the psychological relaxation of young Finnish adults: A pilot study. *PLoS One, 16*(1), e0244799. doi:10.1371/journal.pone.0244799

Brown, B., Dybdal, L., Noonan, C., et al. (2020). Group Gardening in a Native American Community: A Collaborative Approach. *Health Promotion Practice, 21*(4), 611-623. doi:10.1177/1524839919830930

Brown, D. K., Barton, J. L., Pretty, J., & Gladwell, V. F. (2014). Walks4Work: assessing the role of the natural environment in a workplace physical activity intervention. *Scand J Work Environ Health, 40*(4), 390-399. doi:10.5271/sjweh.3421

Calogiuri, G., Evensen, K., Weydahl, A., et al. (2016). Green exercise as a workplace intervention to reduce job stress. Results from a pilot study. *Work, 53*, 99-111. doi:10.3233/WOR-152219

Coventry, P. A., Neale, C., Dyke, A., Pateman, R., & Cinderby, S. (2019). The Mental Health Benefits of Purposeful Activities in Public Green Spaces in Urban and Semi-Urban Neighbourhoods: A Mixed-Methods Pilot and Proof of Concept Study. *Int J Environ Res Public Health, 16*(15). doi:10.3390/ijerph16152712

de Brito, J. N., Pope, Z. C., Mitchell, N. R., et al. (2019). Changes in Psychological and Cognitive Outcomes after Green versus Suburban Walking: A Pilot Crossover Study. *Int J Environ Res Public Health, 16*(16). doi:10.3390/ijerph16162894

Furuyashiki, A., Tabuchi, K., Norikoshi, K., Kobayashi, T., & Oriyama, S. (2019). A comparative study of the physiological and psychological effects of forest bathing (Shinrin-yoku) on working age people with and without depressive tendencies. *Environmental Health and Preventive Medicine, 24*(1), 46. doi:10.1186/s12199-019-0800-1

Gerber, M. M., Callahan, J. L., Moyer, D. N., et al. (2017). Nepali Bhutanese refugees reap support through community gardening. *International Perspectives in Psychology: Research, Practice, Consultation, 6*(1), 17-31. doi:10.1037/ipp0000061

Gidlow, C. J., Jones, M. V., Hurst, G., et al. (2016). Where to put your best foot forward: Psycho-physiological responses to walking in natural and urban environments. *Journal of Environmental Psychology, 45*, 22-29. doi:10.1016/j.jenvp.2015.11.003

Gonzalez, M. T., Hartig, T., Patil, G. G., Martinsen, E. W., & Kirkevold, M. (2011). A prospective study of group cohesiveness in therapeutic horticulture for clinical depression. *Int J Ment Health Nurs, 20*(2), 119-129. doi:10.1111/j.1447-0349.2010.00689.x

Hall, J., Mitchell, G., Webber, C., & Johnson, K. (2018). Effect of horticultural therapy on wellbeing among dementia day care programme participants: A mixed-methods study (Innovative Practice). *Dementia (London), 17*(5), 611-620. doi:10.1177/1471301216643847

Han, K. T., & Wang, P. C. (2018). Empirical Examinations of Effects of Three-Level Green Exercise on Engagement with Nature and Physical Activity. *Int J Environ Res Public Health, 15*(2). doi:10.3390/ijerph15020375

Hawkins, J. L., Smith, A., Backx, K., & Clayton, D. A. (2015). Exercise intensities of gardening tasks within older adult allotment gardeners in Wales. *J Aging Phys Act, 23*(2), 161-168. doi:10.1123/japa.2013-0171

Holt, E. W., Lombard, Q. K., Best, N., Smiley-Smith, S., & Quinn, J. E. (2019). Active and Passive Use of Green Space, Health, and Well-Being amongst University Students. *Int J Environ Res Public Health, 16*(3). doi:10.3390/ijerph16030424

Iwata, Y., Dhubhain, A. N., Brophy, J., et al. (2016). Benefits of Group Walking in Forests for People with Significant Mental Ill-Health. *Ecopsychology, 8*(1), 16-26. doi:10.1089/eco.2015.0045

Johansson, M., Hartig, T., & Staats, H. (2011). Psychological Benefits of Walking: Moderation by Company and Outdoor Environment. *Applied Psychology: Health and Well-Being, 3*(3), 261-280. doi:<https://doi.org/10.1111/j.1758-0854.2011.01051.x>

Kling, H. E., D'Agostino, E. M., Booth, J. V., et al. (2018). The Effect of a Park-Based Physical Activity Program on Cardiovascular, Strength, and Mobility Outcomes Among a Sample of Racially/Ethnically Diverse Adults Aged 55 or Older. *Prev Chronic Dis, 15*, E166. doi:10.5888/pcd15.180326

Korpela, K., Stengård, E., & Jussila, P. (2016). Nature Walks as a Part of Therapeutic Intervention for Depression. *Ecopsychology, 8*(1), 8-15. doi:10.1089/eco.2015.0070

Lanki, T., Siponen, T., Ojala, A., et al. (2017). Acute effects of visits to urban green environments on cardiovascular physiology in women: A field experiment. *Environ Res, 159*, 176-185. doi:10.1016/j.envres.2017.07.039

Lee, J., Park, B. J., Tsunetsugu, Y., et al. (2011). Effect of forest bathing on physiological and psychological responses in young Japanese male subjects. *Public Health, 125*(2), 93-100. doi:10.1016/j.puhe.2010.09.005

Lee, J., Tsunetsugu, Y., Takayama, N., et al. (2014). Influence of forest therapy on cardiovascular relaxation in young adults. *Evid Based Complement Alternat Med, 2014*, 834360. doi:10.1155/2014/834360

Lucke, C., Braumandl, S., Becker, B., et al. (2019). Effects of nature-based mindfulness training on resilience/symptom load in professionals with high work-related stress-levels: findings from the WIN-Study. *Ment Illn, 11*(2), 20-24. doi:10.1108/MIJ-10-2019-0001

Lyu, B., Zeng, C., Deng, S., et al. (2019). Bamboo forest therapy contributes to the regulation of psychological responses. *Journal of Forest Research, 24*(1), 61-70. doi:10.1080/13416979.2018.1538492

Mackay, G. J., & Neill, J. T. (2010). The effect of “green exercise” on state anxiety and the role of exercise duration, intensity, and greenness: A quasi-experimental study. *Psychology of Sport and Exercise, 11*(3), 238-245. doi:10.1016/j.psychsport.2010.01.002

Marselle, M. R., Irvine, K. N., Lorenzo-Arribas, A., & Warber, S. L. (2016). Does perceived restorativeness mediate the effects of perceived biodiversity and perceived naturalness on emotional well-being following group walks in nature? *Journal of Environmental Psychology, 46*, 217-232. doi:<https://doi.org/10.1016/j.jenvp.2016.04.008>

Marselle, M. R., Irvine, K. N., & Warber, S. L. (2013). Walking for well-being: are group walks in certain types of natural environments better for well-being than group walks in urban environments? *Int J Environ Res Public Health, 10*(11), 5603-5628. doi:10.3390/ijerph10115603

Martens, D., Gutscher, H., & Bauer, N. (2011). Walking in “wild” and “tended” urban forests: The impact on psychological well-being. *Journal of Environmental Psychology, 31*(1), 36-44. doi:<https://doi.org/10.1016/j.jenvp.2010.11.001>

McCaffrey, R., & Liehr, P. (2016). The Effect of Reflective Garden Walking on Adults With Increased Levels of Psychological Stress. *Journal of Holistic Nursing, 34*(2), 177-184. doi:10.1177/0898010115594934

Mourão, I., Moreira, M. C., Almeida, T. C., & Brito, L. M. (2019). Perceived changes in well-being and happiness with gardening in urban organic allotments in Portugal. *International Journal of Sustainable Development & World Ecology, 26*(1), 79-89. doi:10.1080/13504509.2018.1469550

Müller-Riemenschneider, F., Petrunoff, N., Yao, J., et al. (2020). Effectiveness of prescribing physical activity in parks to improve health and wellbeing - the park prescription randomized controlled trial. *International Journal of Behavioral Nutrition and Physical Activity, 17*(1), 42. doi:10.1186/s12966-020-00941-8

Ng, K. S. T., Sia, A., Ng, M. K. W., et al. (2018). Effects of Horticultural Therapy on Asian Older Adults: A Randomized Controlled Trial. *Int J Environ Res Public Health, 15*(8). doi:10.3390/ijerph15081705

Oh, Y.-A., Park, S.-A., & Ahn, B.-E. (2018). Assessment of the psychopathological effects of a horticultural therapy program in patients with schizophrenia. *Complementary Therapies in Medicine, 36*, 54-58. doi:<https://doi.org/10.1016/j.ctim.2017.11.019>

Olafsdottir, G., Cloke, P., Schulz, A., et al. (2020). Health Benefits of Walking in Nature: A Randomized Controlled Study Under Conditions of Real-Life Stress. *Environment and Behavior, 52*(3), 248-274. doi:10.1177/0013916518800798

Sin-Ae, P., Lee, A. Y., Hee-Geun, P., et al. (2017). Gardening Intervention as a Low- to Moderate-Intensity Physical Activity for Improving Blood Lipid Profiles, Blood Pressure, Inflammation, and Oxidative Stress in Women over the Age of 70: A Pilot Study. *HortScience horts, 52*(1), 200-205. doi:10.21273/HORTSCI11232-16

Sin-Ae, P., Lee, A. Y., Ki-Cheol, S., Wang-Lok, L., & Dae-Sik, K. (2016). Gardening Intervention for Physical and Psychological Health Benefits in Elderly Women at Community Centers. *HortTechnology hortte, 26*(4), 474-483. doi:10.21273/HORTTECH.26.4.474

Song, C., Ikei, H., Kobayashi, M., et al. (2015). Effect of forest walking on autonomic nervous system activity in middle-aged hypertensive individuals: a pilot study. *Int J Environ Res Public Health, 12*(3), 2687-2699. doi:10.3390/ijerph120302687

Song, C., Ikei, H., Park, B. J., et al. (2018). Psychological Benefits of Walking through Forest Areas. *Int J Environ Res Public Health, 15*(12). doi:10.3390/ijerph15122804

Van Den Berg, A. E., & Custers, M. H. G. (2011). Gardening Promotes Neuroendocrine and Affective Restoration from Stress. *Journal of Health Psychology, 16*(1), 3-11. doi:10.1177/1359105310365577

Vujcic, M., Tomicevic-Dubljevic, J., Grbic, M., et al. (2017). Nature based solution for improving mental health and well-being in urban areas. *Environmental Research, 158*, 385-392. doi:<https://doi.org/10.1016/j.envres.2017.06.030>

Warber, S. L., DeHudy, A. A., Bialko, M. F., Marselle, M. R., & Irvine, K. N. (2015). Addressing “Nature-Deficit Disorder”: A Mixed Methods Pilot Study of Young Adults Attending a Wilderness Camp. *Evidence-Based Complementary and Alternative Medicine, 2015*, 651827. doi:10.1155/2015/651827

Wilson, N., Jones, R., Fleming, S., et al. (2011). Branching Out: The Impact of a Mental Health Ecotherapy Program. *Ecopsychology, 3*(1), 51-57. doi:10.1089/eco.2010.0049

Wood, C. J., Pretty, J., & Griffin, M. (2016). A case–control study of the health and well-being benefits of allotment gardening. *Journal of Public Health, 38*(3), e336-e344. doi:10.1093/pubmed/fdv146

Wyles, K. J., Pahl, S., Holland, M., & Thompson, R. C. (2017). Can Beach Cleans Do More Than Clean-Up Litter? Comparing Beach Cleans to Other Coastal Activities. *Environment and Behavior, 49*(5), 509-535. doi:10.1177/0013916516649412

Yao, Y.-F., & Chen, K.-M. (2017). Effects of horticulture therapy on nursing home older adults in southern Taiwan. *Quality of Life Research, 26*(4), 1007-1014. doi:10.1007/s11136-016-1425-0

Zeng, C., Lyu, B., Deng, S., et al. (2020). Benefits of a Three-Day Bamboo Forest Therapy Session on the Physiological Responses of University Students. *International Journal of Environmental Research and Public Health, 17*(9), 3238. Retrieved from <https://www.mdpi.com/1660-4601/17/9/3238>
