# Supplementary material for: Nature-based outdoor activities for mental and physical health: Systematic review and meta-analysis
Source: SSM Popul Health. 2021 Oct 1;16:100934. doi: 10.1016/j.ssmph.2021.100934 (PMC8498096; doi:10.1016/j.ssmph.2021.100934)
Supplement: Multimedia component 3 [file mmc3.docx]

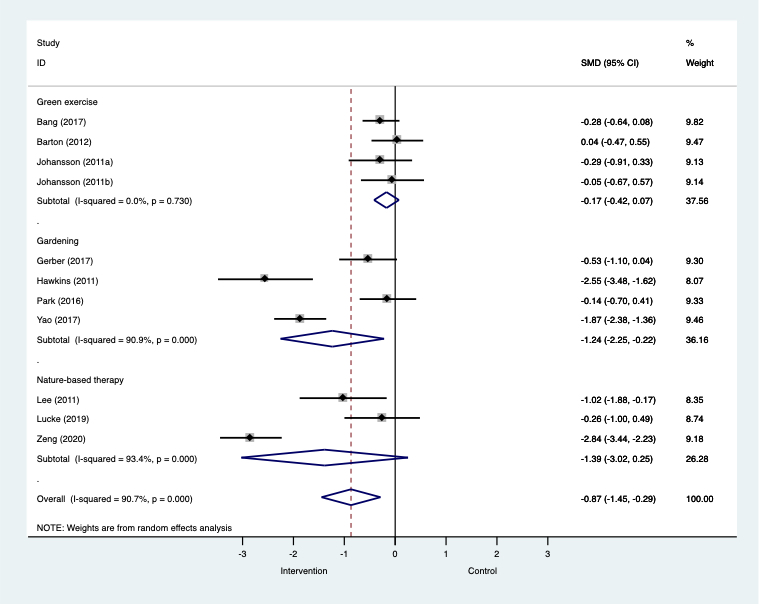


**Figure C.1 Meta-analysis of controlled studies of nature-based interventions for depressive mood versus control at post-intervention across all populations**

The size of the grey box reflects how much weight each study received in the meta-analysis (i.e., the larger the box the more this study contributed to the pooled effect represented by the blue diamond). Black bars represent the 95% CI for the SMD in each study.

CI = confidence interval; SMD = standardised mean difference


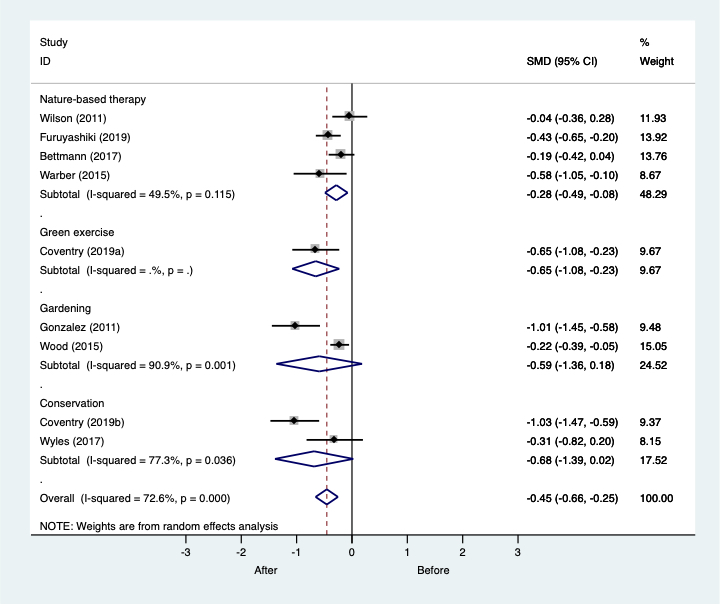


**Figure C.2 Meta-analysis of single group before and after studies of nature-based interventions for depressive mood across all populations**

The size of the grey box reflects how much weight each study received in the meta-analysis (i.e., the larger the box the more this study contributed to the pooled effect represented by the blue diamond). Black bars represent the 95% CI for the SMD in each study.

CI = confidence interval; SMD = standardised mean difference


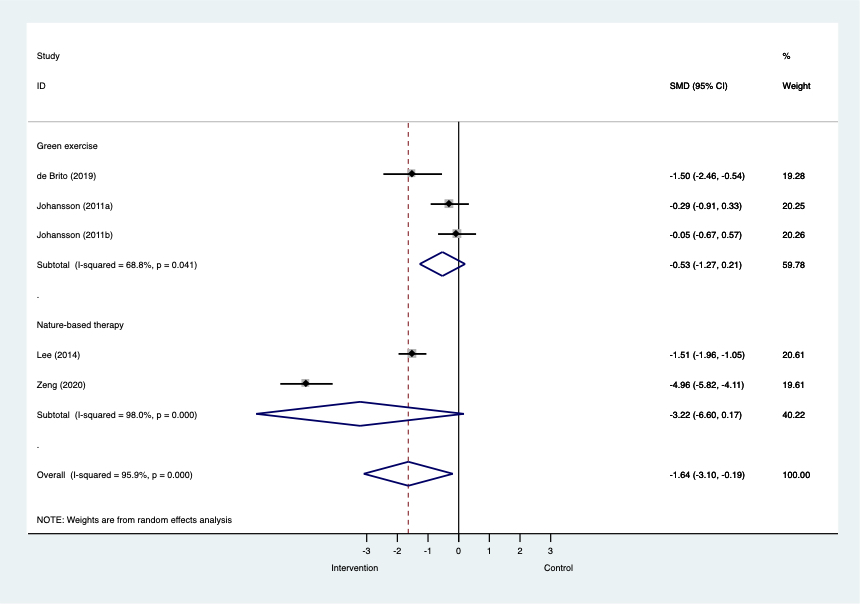


**Figure C.3 Meta-analysis of controlled studies of nature-based interventions for anxiety versus control at post-intervention across all populations**

The size of the grey box reflects how much weight each study received in the meta-analysis (i.e., the larger the box the more this study contributed to the pooled effect represented by the blue diamond). Black bars represent the 95% CI for the SMD in each study.

CI = confidence interval; SMD = standardised mean difference


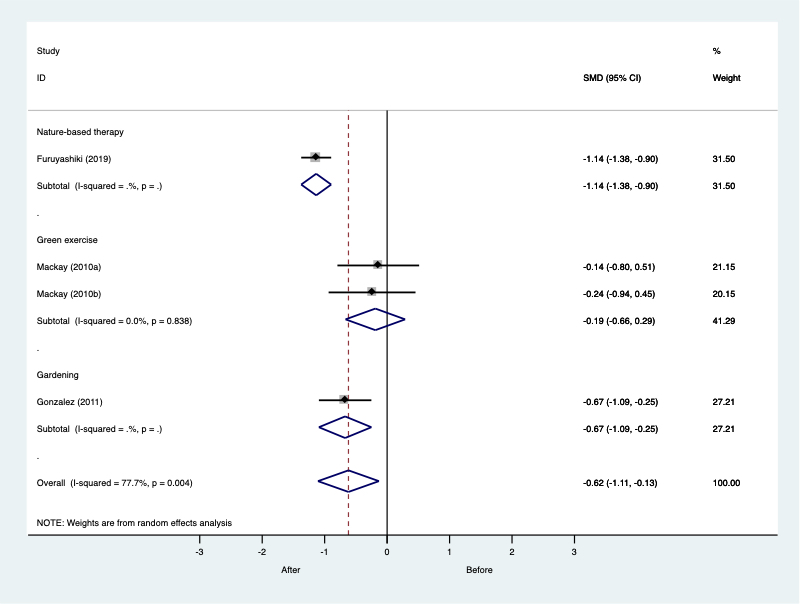


**Figure C.4** **Meta-analysis of single group before and after studies of nature-based interventions for anxiety across all populations**

The size of the grey box reflects how much weight each study received in the meta-analysis (i.e., the larger the box the more this study contributed to the pooled effect represented by the blue diamond). Black bars represent the 95% CI for the SMD in each study.

CI = confidence interval; SMD = standardised mean difference


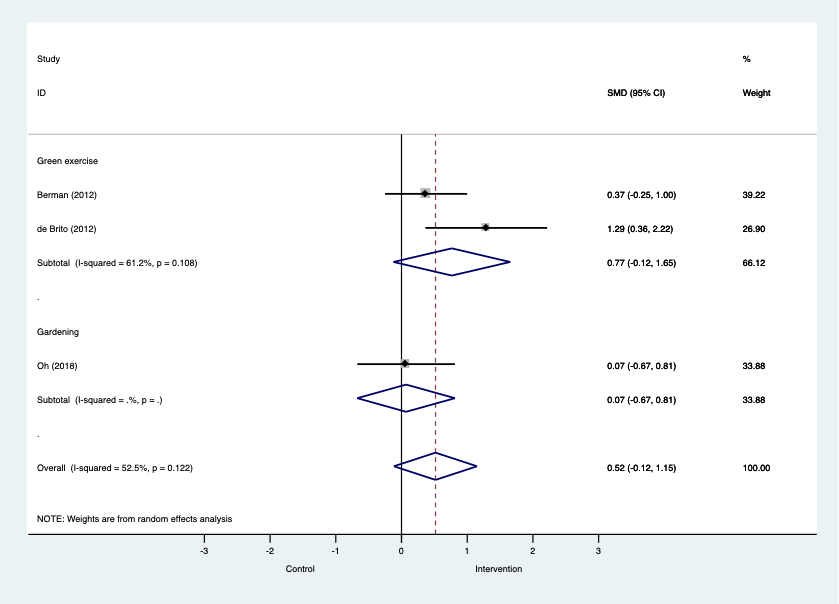


**Figure C.5** **Meta-analysis of controlled studies of nature-based interventions for positive affect versus control at post-intervention across all populations**

The size of the grey box reflects how much weight each study received in the meta-analysis (i.e., the larger the box the more this study contributed to the pooled effect represented by the blue diamond). Black bars represent the 95% CI for the SMD in each study.

CI = confidence interval; SMD = standardised mean difference


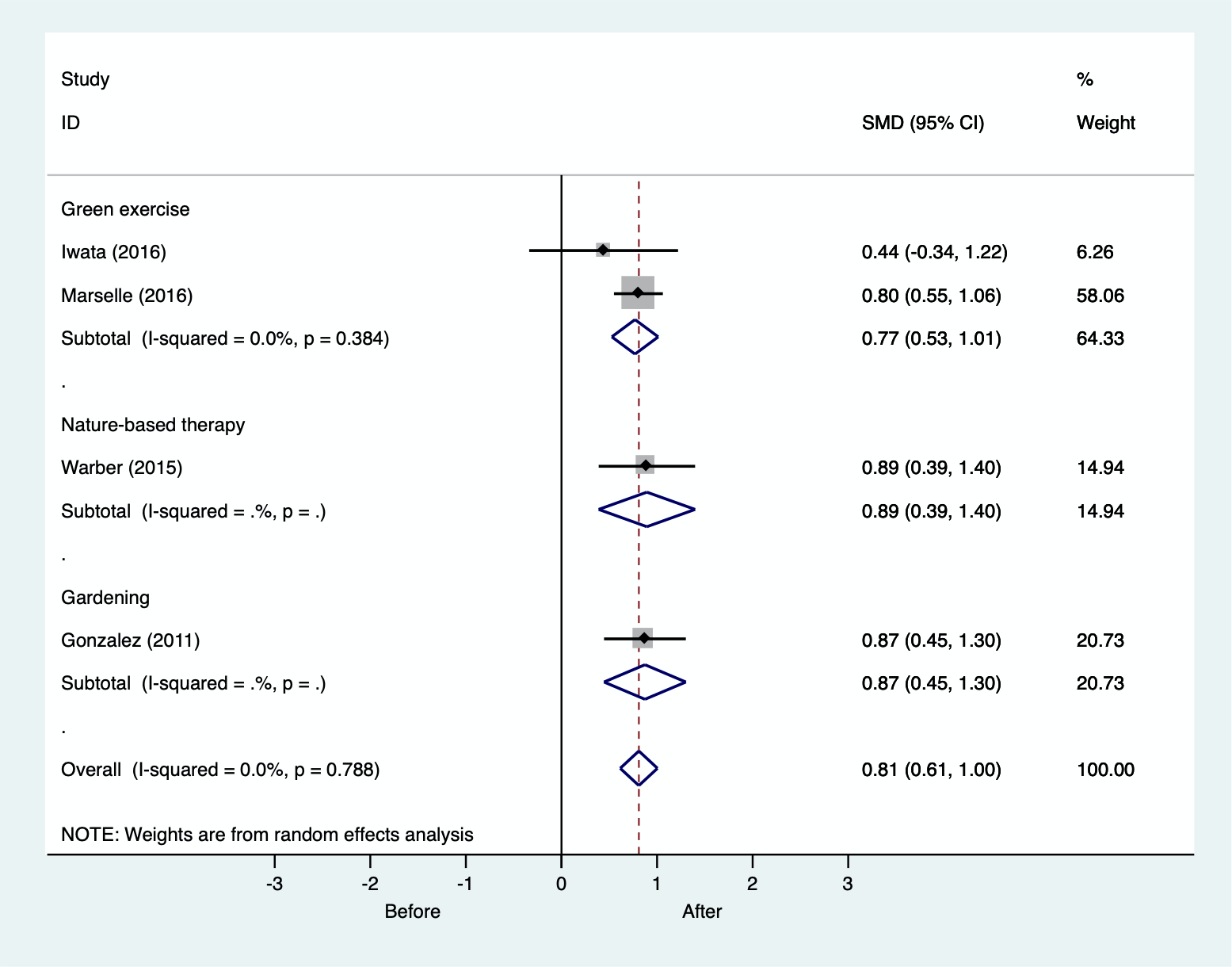


**Figure C.6** **Meta-analysis of single group before and after studies of nature-based interventions for positive affect across all populations**

The size of the grey box reflects how much weight each study received in the meta-analysis (i.e., the larger the box the more this study contributed to the pooled effect represented by the blue diamond). Black bars represent the 95% CI for the SMD in each study.

CI = confidence interval; SMD = standardised mean difference


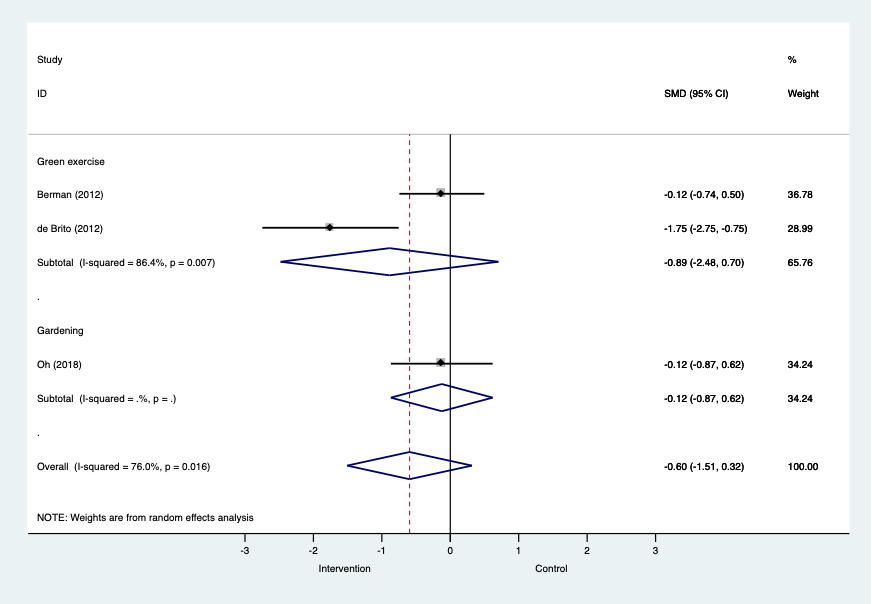


**Figure C.7** **Meta-analysis of controlled studies of nature-based interventions for negative affect versus control at post-intervention across all populations**

The size of the grey box reflects how much weight each study received in the meta-analysis (i.e., the larger the box the more this study contributed to the pooled effect represented by the blue diamond). Black bars represent the 95% CI for the SMD in each study.

CI = confidence interval; SMD = standardised mean difference


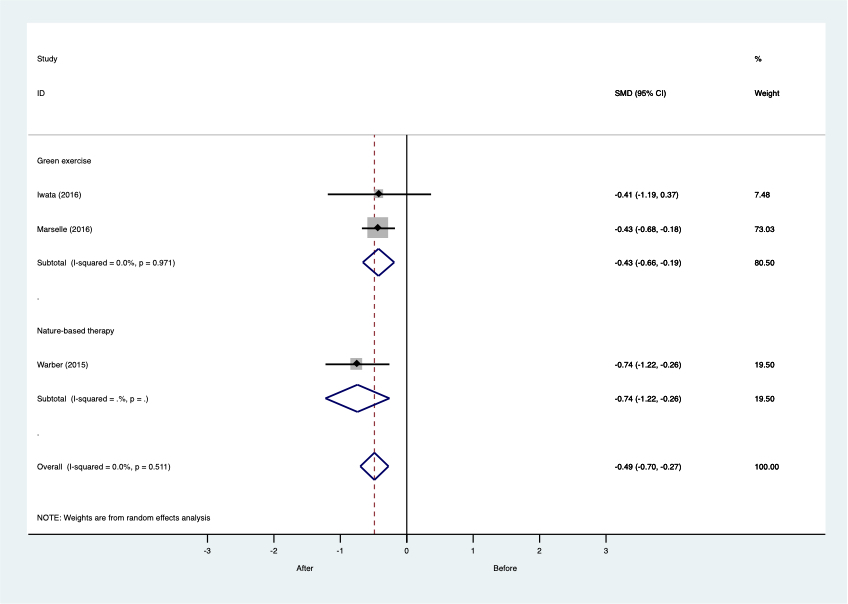


**Figure C.8** **Meta-analysis of single group before and after studies of nature-based interventions for negative affect across all populations**

The size of the grey box reflects how much weight each study received in the meta-analysis (i.e., the larger the box the more this study contributed to the pooled effect represented by the blue diamond). Black bars represent the 95% CI for the SMD in each study.

CI = confidence interval; SMD = standardised mean difference


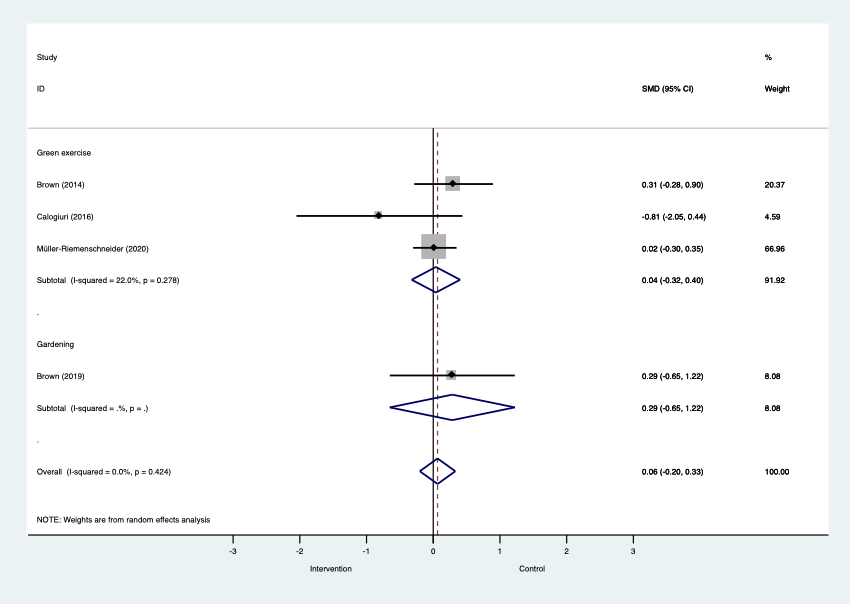


**Figure C.9 Meta-analysis of RCTs of nature-based interventions for systolic blood pressure versus control at post-intervention across all populations**

The size of the grey box reflects how much weight each study received in the meta-analysis (i.e., the larger the box the more this study contributed to the pooled effect represented by the blue diamond). Black bars represent the 95% CI for the SMD in each study.

CI = confidence interval; RCT = randomised controlled trial; SMD = standardised mean difference


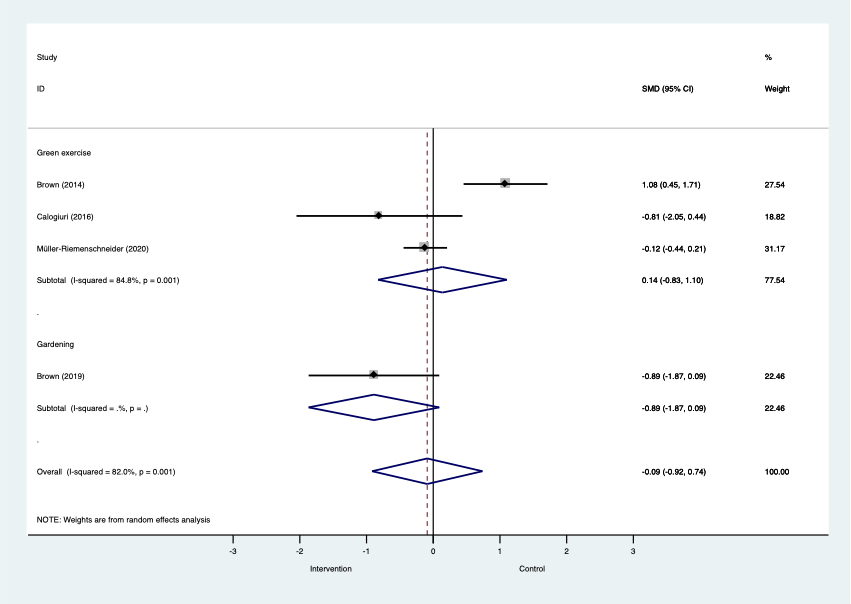


**Figure C.10 Meta-analysis of RCTs of nature-based interventions for diastolic blood pressure versus control at post-intervention across all populations**

The size of the grey box reflects how much weight each study received in the meta-analysis (i.e., the larger the box the more this study contributed to the pooled effect represented by the blue diamond). Black bars represent the 95% CI for the SMD in each study.

CI = confidence interval; RCT = randomised controlled trial; SMD = standardised mean difference


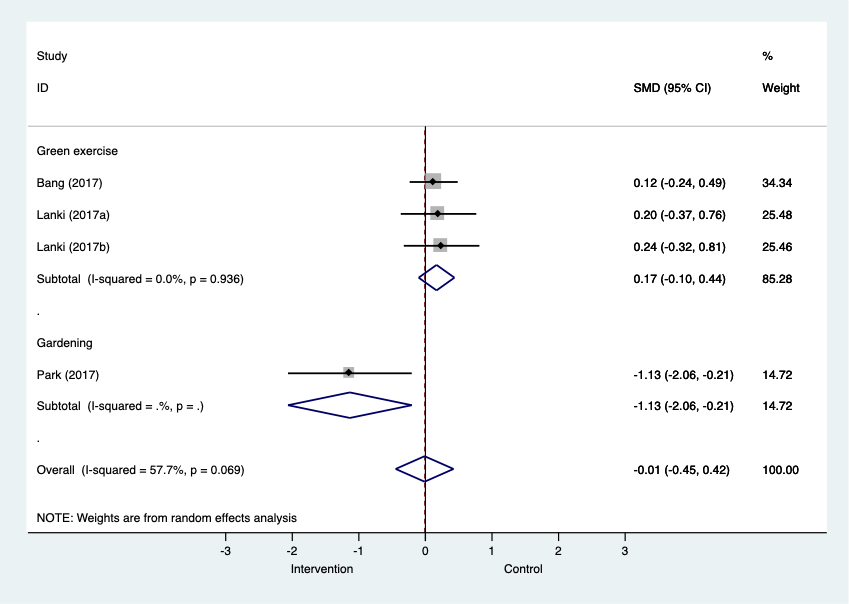


**Figure C.11 Meta-analysis of controlled studies of nature-based interventions for systolic blood pressure versus control at post-intervention across all populations**

The size of the grey box reflects how much weight each study received in the meta-analysis (i.e., the larger the box the more this study contributed to the pooled effect represented by the blue diamond). Black bars represent the 95% CI for the SMD in each study.

CI = confidence interval; SMD = standardised mean difference


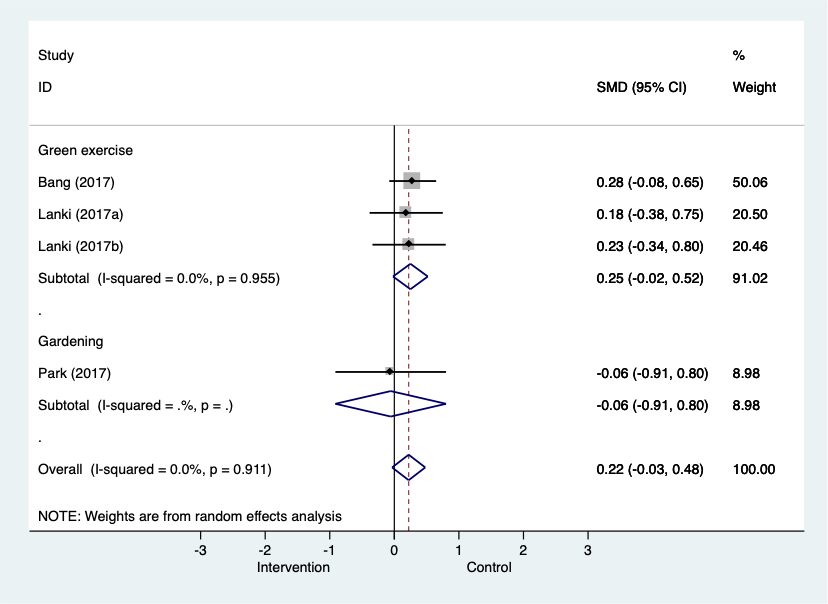


**Figure C.12 Meta-analysis of controlled studies of nature-based interventions for diastolic blood pressure versus control at post-intervention across all populations**

The size of the grey box reflects how much weight each study received in the meta-analysis (i.e., the larger the box the more this study contributed to the pooled effect represented by the blue diamond). Black bars represent the 95% CI for the SMD in each study.

CI = confidence interval; SMD = standardised mean difference
